# An ethnobotanical survey of medicinal and edible plants of Yalo Woreda in Afar regional state, Ethiopia

**DOI:** 10.1186/s13002-017-0166-7

**Published:** 2017-07-05

**Authors:** Tilahun Teklehaymanot

**Affiliations:** Endod, and Other Medicinal Plants Research Unit, Aklilu Lemma Institute of Pathobiology, Addis Ababa University, P. O. Box, 56478 Addis Ababa, Ethiopia

**Keywords:** Afar people, Yalo Woreda, Ethnobotanical study, Traditional knowledge, Medicinal plants, Edible plants, Ethiopia

## Abstract

**Background:**

The Afar people inhabit the sub-arid and arid part of Ethiopia. Recurrent drought and invasive encroaching plants are taking out plants that have cultural importance, and threaten the biodiversity and the associated traditional knowledge. Thus, the aim of the current study is to conduct an ethnobotanical survey and document medicinal and edible plants in Yalo Woreda in Afar regional state.

**Methods:**

A cross-sectional ethnobotanical study was carried out in eight kebeles of Yalo Woreda from October 2015 to December 2016. One hundred sixty informants were selected using purposive sampling. The data on diseases, medicinal and edible plants were collected using semi-structure interview and group discussion. The statistical methods, informant consensus factor, fidelity level, and preference ranking were conducted to analyze the data.

**Results:**

One hundred and six plants were reported; gender and age differences had implication on the number of plants reported by informants. The knowledge of medicinal plants among informants of each kebele was not different (p < 0.5) and was not associated in particular with the religious establishment in the kebeles (informant*kebeles, Eta square = 0.19). Family Fabaceae was the major plant species, and shrubs (44%) were dominant plants reported. Leaf (52.94%) and oral (68%) were primary plant part used for remedy preparation and route of application, respectively. The plants with low fidelity values *Indigofera articulata* (0.25), *Cadaba farinosa* (0.22), *Cadaba rotundifolia* (0.19), and *Acalypha fruticosa* (0.15) were used to treat the category of diseases with high informant consensus value (0.69). Sixteen edible plants were identified that were consumed during wet and dry seasons. *Balanites aegyptiaca, Balanites rotundifolia,* and *Dobera glabra* were ‘famine food’ that were collected and stored for years.

**Conclusion:**

People in Yalo Woreda are more dependent on natural resources of the area for their livelihood. The threat of climatic change and encroaching invasive plants on medicinal and edible plants affects the traditional use of plants in the Yalo Woreda. The conservation of the plants in the home garden and natural habitat and integration of edible plants into agroforestry development programs in sub-arid and arid regions has to be encouraged to conserve plants of medical and economic importance.

**Electronic supplementary material:**

The online version of this article (doi:10.1186/s13002-017-0166-7) contains supplementary material, which is available to authorized users.

## Background

Afar people are pastoralists that live in the Great Rift Valley of Ethiopia. Afar Regional State is located in the eastern part of Ethiopia, bordering the State of Eritrea in the northeast, Tigray in the northwest, Amhara in the southwest, Oromia in the south, the State of Somalia in the southeast and the Republic of Djibouti in the east [1]. The Erta Ale active volcano in the Danakil depression (120 m below sea level), the lowest point below sea level in Ethiopia is found in the northern part of the region. The Afar Regional state is subdivided into five administrative zones. The population of the area based on the 2007 census is 1,390,273 consisting of 775,117 men and 615,156 women with an estimated density of 14.38 people per square kilometer [2]. The estimated area of the region is 96,707 Km^2^ and lies between 8° 40′13″ to 14° 27′ 29″ N latitude and 39° 51′13″ to 42° 23′03″ E longitude. The climate of the region is semi-arid to arid with erratic rainfall, and altitude ranges from 120 m below sea level up to 1500 masl. The vegetation of the area is *Acacia-Commiphora* (Small-Leaved Deciduous) Woodland; 31.5% shrubland, 14.8% grassland, 1.75% woodland and 0.11% forestland with a large area (49.6%) of rocky, sandy and exposed soil [3–5].

The people in the Afar region have the lowest health and education coverage in the country with the highest food insecurity [6]. They are a traditional society that has native and unique information exchange system by word of mouth called ‘*Dagu,’* which their livelihood is very much dependent on the information transferred through *Dagu* system. The information ranges from weather to availability of grazing lands for their animals, and peace and security of the region [4]. Nevertheless, the cultural transformation, expansion of modern education and development in the area could detach the younger generation from such cultural values and pastoral systems that lead to loss of traditional knowledge in general, and knowledge of medicinal and edible plants in particular [7, 8].

The Afar people mostly depend on their animals and their products, and vegetation of the area as a resource for their livelihood. The animal products milk, meat, and butter are used as the primary diet, and live animals, hide, and skin generates the pastoral’s economy. The vegetation of the area is associated with significant uses such as medicine, food for human and livestock, firewood, charcoal, building materials and for making household goods [1, 9, 10]. They often move from one place to another in search of food and water for their livestock [6]. The Afar people’s livestock proportion varies accordingly with the vegetation cover of the locality. The dominant animal in the reverine forest and with better grassland are cattle and sheep, in the drier parts of the region camel and goat are dominant, and in the arid zones camels are widely dominant animal [1].

The vegetation of the area is severely affected by increased overexploitation for charcoal production and clearing forests for settlement and agriculture [3]. Some of the woody and grass species are declining such as *Acacia nilotica* (L.) Willd. ex Del.*, Acacia senegal* (L.) Willd., *Acacia tortilis* (Forssk.) Schweinf., *Balanites aegyptiaca* (van Tieghem) Blatter*, Cordia gharaf* (Forsk.)*, Ziziphus spina-christi* (L.) Desf. *Cenchrus* species and *Cynodon* species are the most affected and young seedlings are not usually seen growing [1, 10–14]. The recent incidents in the Afar region is the invasive encroaching plants; *Prosopis juliflora* (Sw.) DC. (Woyane), *Parthenium hysterophorus* L. (white top weed), and *Cryptostegia grandiflora* Roxb. ex R. Br*.* (rubber vine) are taking out multipurpose trees, grassland, and bushes and transforming the region to the mono-species thick forest. *Prosopis juliflora* has an effect on the total biodiversity of the area by reducing their abundance, distribution, and ecological function and replacing grassland and natural forests. It is a cause for the fast disappearing of plants used by the people as medicine and food supplements in normal time and during a food shortage. Also, *P. hysterophorus* and *C. grandiflora* are a threat to grassland and livelihood of the people in the region. The vast destruction of the natural habitats leads to a gradual disappearance of the associated traditional knowledge of medicinal and edible plants [1, 6, 14–20].

The Afar people, in the past, depends on milk and its’ products as main diet and edible plants of the area as a source of food in harsh times. The intensification and severity of drought caused by the climatic change, in the pastoral area, complicated and disrupted the relationship between the society and natural environment [1]. At present, because of recurrent severe drought, massive loss of livestock and dependence on relief food, the Afar People has shifted their feeding habit, and it is a cause for loss of traditional knowledge of edible plants by the younger generation [8]. According to Alemu [21], the Afar elders were aware that their traditional way of life is changing in several respects including effects of cultivation, overexploitation, and bush encroachment that would result in a declining trend in all natural resources. Moreover, Atanga et al. [6] reported, based on the interview with the older livestock herders, that 63% of grazing plant species has disappeared within 25 years from the rangelands. Hence, there is a gradual erosion of knowledge of medicinal and edible plants in the society, which requires formal ethnobotanical documentation [1, 4, 19, 20]. On the other hand, the ethnobotanical studies conducted in the Afar Region are few, and most of these studies are carried out in Awash Park where the majority of the inhabitants are Oromo People [9, 10, 14–23]. Thus, the aim of this study is to survey the traditional knowledge of medicinal and edible plants of Afar People in Yalo Woreda (District), Zone 4, Afar Regional state. The study may be used as a foundation for pharmacological and nutritional studies and identification of useful plants of the region for conservation.

## Methods

### Study area

**Fig. 1:**
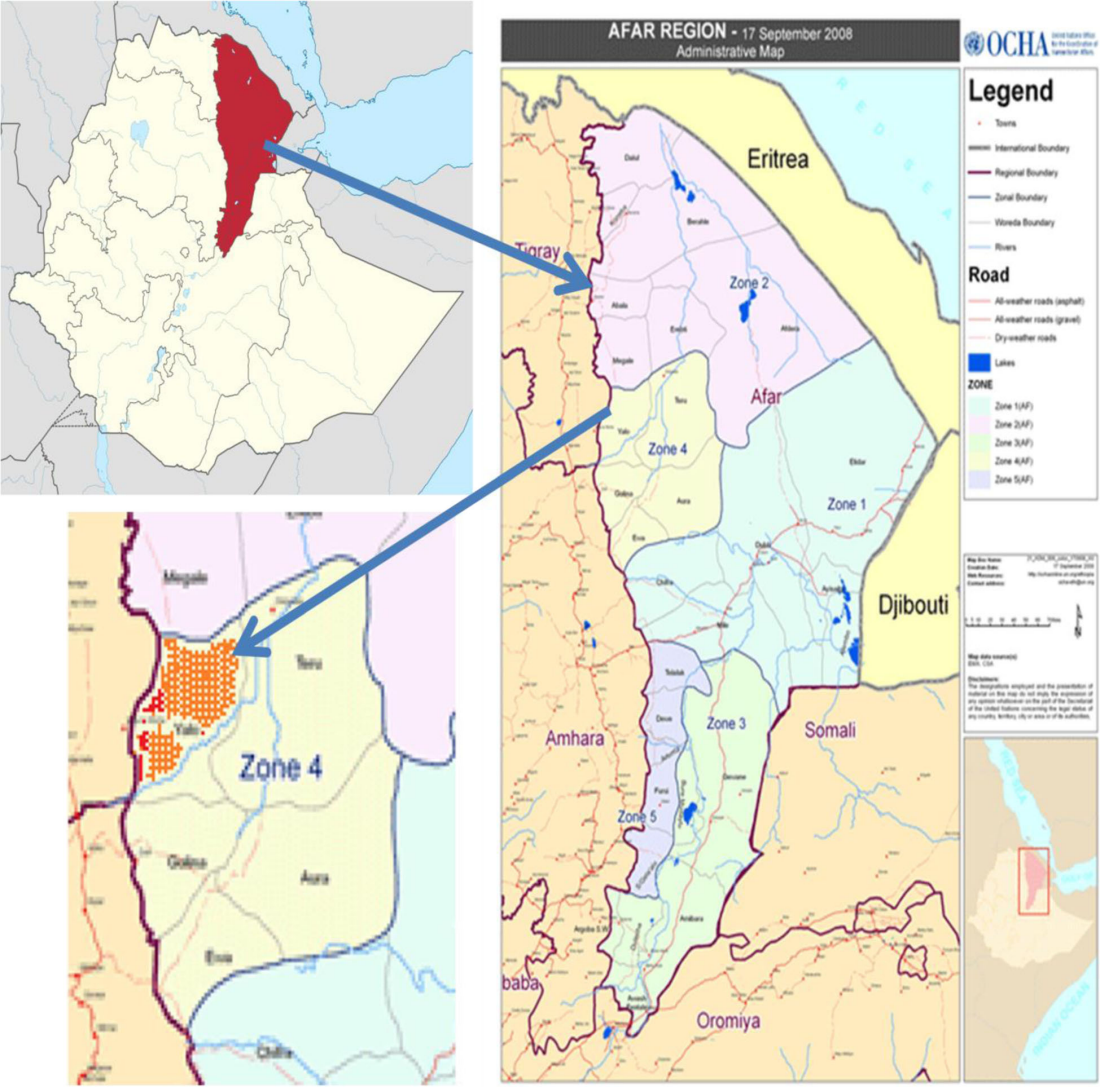
Study area. Orange tinted part shows Yalo Woreda in Afar Regional State

A cross-sectional ethnobotanical study was conducted in eight kebeles (smallest administrative division) of Yalo Woreda from October 2015 to December 2016. Yalo Woreda is located 732 km from Addis Ababa in Zone 4, the western part of Afar Regional State; bordering Megalea Woreda in the north, Gelina Woreda in the south, Teru Woreda in the east, and Alamata Woreda in the west (Fig. 1). The landscape varies from undulating hills to flat land, and the area of the Woreda is 822.75 Km^2^. The climate of the Woreda is kola (Lowland) with average minimum temperature of 21 °C and maximum 38 °C and with 500 mm annual average rainfall [24]. The kebeles are Dibina (12°23′45″ N, 39°52′58″ E, 890 masl); Gidi Elea (12°16′46″ N, 39°54′31″ E, 974 masl); Kolina Gabulea (12°23′24″ N, 39°56′57″ E, 811 masl); Mesgid (12° 21′45″ N, 39°52′44″ E, 865 masl); Rekrek (12°23′9″ N, 39°52′30″ E, 893 masl); Reku Dora (12°16′46″ N, 39°54′31″ E, 974 masl); Waleae (12°17′36″ N, 39°24′11″ E, 890 masl) and Wudayili (12° 19′26″ N, 39°44′21″ E, 874 masl).

The population of Yalo Woreda, based on 2006 National census, was 54,263 in which 24,418 were female, 29,845 were males, 46,511 were rural, and 7752 were urban dwellers. The majority of the population (95%) is pastoralists, and 5% are semi-pastoralists [24]. The top ten causes of morbidity in Yalo Woreda are malaria, non-bloody diarrhea, pneumonia, lung and acute upper respiratory tract infection, acute febrile illness, urinary tract infection, bloody diarrhea, infection of the skin and subcutaneous tissue, dyspepsia and severe malnutrition [25]. In Yalo Woreda, there are 2220 cattle, 20,190 sheep, 73,389 goats, 14,819 camels and 2733 equine. The top ten veterinary important diseases are PPR, pox, pasteurellosis, CCPP, external parasite, internal parasite, ORF, foot rot, salmonellosis, and brucellosis [26].

### Selection of informants and collection of data

The informants were selected with the assistance of elders, and Kebele Administrative Officers. One hundred and sixty informants, 20 from each kebele, were chosen from eight kebeles using purposive sampling. Thirty-seven were females, and 123 were males. The purpose of the study was briefed to the informant, elders and Kebele Administrators to get their consent before collection of data. After receiving their informed consent, ethnobotanical data were collected using semi-structured interview, observations, field visits, and group discussion with the assistance of a native translator. The data collected were the type of diseases treated, the name of plants used for treatment, parts used, methods of preparation and dosage. The information gathered on edible plants were the name of edible plants, parts used as a food source, and seasons or availability of an edible part, and time of consumption [27].

### Collections of plant specimens and identifications

The voucher plant specimens were collected from Yalo Woreda during field walks with the informants, and initial identification was conducted on the site. The specimens of plants were pressed and taken to Aklilu Lemma Institute of Pathology for identification. The specimens were further identified by an expert at National Herbarium, Addis Ababa University by using Flora of Eretria and Ethiopia and comparing with herbaria samples and deposited in National Herbarium of Addis Ababa University.

### Data analysis

A descriptive statistics, percentages, and frequency were used to analyze ethnobotanical data with Microsoft Excel 2007. Statistical test; one-way analysis of variance was performed with SPSS Advanced Statistics 20.0 to compare knowledge of male and female; among age groups, and kebeles.

### Informant consensus factor (ICF), fidelity level (FL) and preference ranking

The diseases and remedies reported were grouped into ten categories based on the top ten diseases in the Woreda. The categories were acute febrile illness and malaria; external injury, eye, ear nose, and mouth infections; gastrointestinal disease; impotence; internal parasites; liver infection; respiratory and lung infection; skin and subcutaneous tissue infection; swellings and cancer; and urinary tract infection. The informant consensus factor (ICF) was calculated to determine the agreements of the informants on each remedy using the formula $ICF=\frac{n_{ur}-{n}_t}{n_{ur}-1}$. Where number of use citations in each category (n_ur_) minus the number of species used (n_t_), divided by the number of use citations in each category minus one where n_ur_ is the number of use citations and n_t_ is the number of species used [28].

Fidelity Level (FL) was calculated to determine the percentage of informants reported the uses of a medicinal plant as a remedy for the same major ailment using the formula $\mathrm{FL}\left(\%\right)=\frac{Ip}{Iu}\times 100$. Where *I*p is the number of informants who independently indicated the use of a species for the same major ailment and *Iu* the total number of informants who mentioned the plant for any major ailment [29].

Preference ranking on plants that were reported by 15 and above informants that were used as a treatment for multiple diseases was conducted. Eight informants, one from each Kebele based on the number of medicinal plants reported by each informant, were selected to rank the plants according to their preference [27]. The informants were briefed on the marking of the plants that the most preferred was give the highest points (10) and least preferred was given the lowest point (1). Ten small plots labeled with one to ten was made, and each respondent was asked to put the plants in each plot. The mark given for each plant was recorded accordingly.

### Ethical consideration

Institute Review Board of Aklilu Lemma Institute of Pathobiology, Addis Ababa University, reviewed and ethically approved the study. The Yalo Woreda Administrators were enlightened about the importance of the documentation of medicinal and edible plants in the Woreda before getting their permission to conduct an ethnobotanical survey in each Kebele. Likewise, Kebele Officers, elders, and informants were briefed about the primary objective of the study to enable then to decide whether to participate in the study or not before receiving their consents.

## Results

### Traditional knowledge

**Fig. 2:**
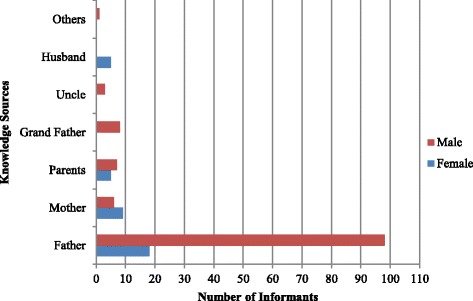
Traditional knowledge sources of medicinal and edible plants

The study revealed the rich knowledge of medicinal plants in Yalo woreda that was indicated by the number and diversity of medicinal plants reported. Informants reported 106 medicinal and edible plants. The age of female informants was from 18 to 70 with a mean age of 39.38 ± 2.31 years and males from 20 to 80 with a mean age of 42.30 ± 1.10 years. The number of plants reported by females was ranging from one to six and males from one to 22. The average number of medicinal plants identified by females (2.03 ± 0.17) was less than male (3.89 ± 0.17), and the difference was significant (*p* < 0.05). Male parents were the primary source of traditional knowledge (TK) in the study area (Fig. 2). Male informants reported forty-two veterinary importance plants of which females reported only two, *Acacia oerfota* (Forssk.) Schweinf. as a treatment for goat sickness and *B. aegyptiaca* as a remedy for cow skin infection and itching. The average number of plants reported by young informants (18 ≤ 39) was 2.31 ± 0.20, old informants (40 ≤ 70) was 3.72 ± 0.18, and the difference was significant (*p* < 05). The knowledge of informants was no associated with kebeles (informant*kebeles, Eta square = 0.19). The difference among the kebeles in the number of medicinal plants reported by each informant was not significant (*p* > 0.05).

### Medicinal plants of public health importance

The informants reported 102 medicinal plants with public health importance distributed into 46 family and 79 genera. Family Fabaceae had 16 Species, followed by Lamiaceae and Solanaceae with six species each, Capparidaceae with five species, Boraginaceae and Loranthaceae with four species each, Acanthaceae, Amaranthaceae, Apocynaceae, Asteraceae, Cucurbitaceae, Euphorbiaceae, Tiliaceae, and Vitaceae with three species each (Table 1; Additional file 1). All the medicinal plants were harvested from natural vegetation.

**Table 1:** Medicinal plants used for treatment of human and animal illness in Yalow Woreda, 2016

Family	Scientific Name [References of other studies]	Disease	Parts	Application (Animal type)	FL	Voucher No.
Acanthaceae	*Barleria homoiotrichia* C. B. Clarke	Mumps	F	Topical	0.60	YA 046
		Herpes Zoster	B	Oral, nasal, body wash		
	*Justicia schimperiana* (Hochst.ex Nees) T. Anders	Somnambulism	F	Oral, body wash	0.33	YA 022
		Diphtheria	B	Oral		
		Retained placenta	R	Oral (Camel)		
	*Ruellia patula* Jacq.	Swelling	L	Oral	0.67	YA 021
		Meningitis	WP	Nasal		
Aizoaceae	*Aizoon canariensis* L.	Head injury	L	Topical	1.00	YA 089
		Schizophrenia	WP	Inhaling		
	*Trianthema portulacastrum* L.*	Hand swelling	L	Topical	0.67	YA 014
		Snake Bite	WP	Nasal		
		Herpes Zoster	WP	Nasal		
Alliaceae	*Allium sativum* L.	Somnambulism	Fr	Oral, body wash	0.50	YA 004
		Skin infection	Fr	Topical		
		PPR	Fr	Oral (Cattle)		
Aloaceae	*Aloe trichosantha* A. Berger *	Diphtheria	L,S	Oral, body wash	0.30	YA 098
		Eye sickness	La	Eye-drop		
		Snake bite	R	Oral, nasal, body wash		
		Malaria	L	Oral		
		Breast infection	L,S	Oral, nasal, body wash		
		Delayed placenta	L,R	Oral		
		Black leg/ joint illness	R	Topical (Cow oxen)		
		Brucellosis	L, S	Oral, nasal, ear, body wash (Camel, cow, goat)		
Amaranthaceae	*Aerva javanica* (Burm.f.) Schultes	Breast infection	L	Nasal, body wash	0.31	YA 077
		Epistaxis	R	Oral, nasal		
		Wound	L	Topical		
		STIs	R	Oral, body wash		
		Mastitis and contagious agalactia	L	Oral, nasal, body wash (Goat, cow)		
		PPR	R	Nasal (Goat)		
	*Celosia polystachia* (Forssk.) C.C. Towns.*	Breast cancer	L	Oral, nasal, topical	0.47	YA 103
		Epilepsy	L	Oral		
		Dyspepsia	L	Oral		
		Typhoid	WP	Oral, wash body		
		Jaundice	L	Oral		
		EPTB	L	Oral		
		Lung infection	YP	Oral		
		Mumps	WP	Oral		
		Blackleg	R	Oral, nasal (Cattle)		
		Mastitis and contagious agalactia	L	Oral, nasal, ear, body wash (Camel, cow, goat)		
		PPR	L	Oral (Cattle)		
	*Sericocomopsis pallida* (S. Moore) Schinz*	Head wound	L	Topical	1.00	YA 002
Anacardiaceae	*Rhus natalensis* Bernh. ex C. Krauss	Swelling on body	L	Oral	0.33	YA 012
		Snake bite	R	Oral, tying		
		Topical wound, External body infection	L	Topical insertion (Camel, Cattle)		
Apiaceae	*Ferula communis* L.	Somnambulism	F	Oral, body wash	0.67	YA 094
		Dyspepsia	F	Oral		
		Schizophrenia	L	Body wash		
		PPR	Fr	Oral (Cattle)		
Apocynaceae	*Acokanthera schimperi* (A. DC.) Schwein	Kidney infection	R	Oral, topical	0.50	YA 027
		Eye Infection	L/La	Eye-drop		
		PPR	L	Oral, nasal (Cattle Camel)		
	*Pergularia tomentosa* L.	Evil Eye	R	Body wash	0.50	YA 024
		Snake Bite	L	Oral, nasal, ear		
Aristolochiaceae	*Aristolochia bracteolata* Lam.	Diphtheria	WP	Topical	0.71	YA 087
		Jaundice	WP	Oral		
		Swelling on body	L	Oral, body wash		
		Snake Bite	R	Oral		
		Eye infection	L	Eye-drop		
Asclepiadaceae	*Calotropis procera* (Ait.) Ait.f.	Typhoid	FB, L	Oral, Nasal	0.29	YA 043
		Dyspepsia	F	Oral		
		Breast swelling	L	Nasal, Topical		
		Herpes Zoster	L	Nasal		
		Mouth infection	B	Oral, mouth wash		
		Black leg	YP	Oral, nasal (Cattle)		
		Anthrax	S, L	Oral (Cattle)		
		Mastitis and contagious agalactia	L	Oral, nasal, body wash (Camel, cow)		
	*Kanahia laniflora* (Forssk.) R.Br.*	Flue	R	Sniffing, nasal	0.40	YA 073
		Asthma	L	Inhaling		
		Angina	R	Oral		
		Schizophrenia	WP	Oral, nasal		
Asteraceae	*Kleinia squarrosa* Cufod.*	Somnambulism	L	Body wash	0.50	YA 020
		Eye infection	L	Eye-drop		
	*Parthenium hysterophorus* L.*	Somnambulism	F	Oral	1.00	YA 093
	*Xanthium strumarium* L.	Dyspepsia	WP	Oral, nasal	1.00	YA 030
Balanitaceae	*Balanites aegyptiaca* (van Tieghem) Blatter	Infant sickness	R	Oral	0.43	YA 095
		Breast cancer	L	Oral, nasal, body wash		
		Lung Infection	R,B	Oral, nasal		
		Kola kusil,	L	Oral, topical		
		Mumps	L	Oral, nasal, body wash		
		Dysentery	L.B	Oral		
		Herpes Zoster	B /R	Oral, nasal		
		Devil Disease	B	Oral		
		Brucellosis	L	Oral (Cow, goat)		
		Blackleg	R	Oral (Cow, goat)		
		Trypanosomiasis	R	Oral (Cow, goat)		
		CCPP	R	Oral (Cow, goat)		
		Pastuerollosis	L	Oral (Cattle)		
		Bovine TB	B	Oral, nasal (Cattle)		
	*Balanites rotundifolia* (van Tiegn.) Blatter	Malaria	L	Oral	0.43	YA 008
		Infant sickness	UP	Oral, nasal, ear, washing		
		Mumps	R	Oral, nasal, body wash		
		Flue, cough	L	Nasal, oral		
		Breast cancer	L	Oral, topical		
		ETPB	L	Oral, nasal, topical		
		Jaundice	L,B	Oral		
		Dyspepsia	L,R	Oral		
		Eye injury	R	Eye-drop		
		Bullet injury	B,S	Tying/ topical		
		Herpes Zoster	L	Nasal, body wash		
		Blackleg	R	Nasal (Goat)		
		Mastitis and contagious agalactia	L	Oral (Cow, goat, Camel)		
		Bovine pastuerollosis	L	Oral (Cattle)		
		PPR	R	Nasal (Goat, sheep)		
		Bovine TB	YP	Oral, nasal (Cattle)		
Boraginaceae	*Bourreria orbicularis* (Hutch. & E.A. Bruce) Thulin	Breast cancer	L	Oral	1.00	YA 065
		Brucellosis	L	Oral (Cow, goat)		
		Bovine TB	Fr	Oral, nasal (Cattle)		
	*Cordia sinensis* Lam.	Arthritis	WP	Topical insertion	0.67	YA 072
		Topical wound infection	B	Topical (Cow, goat, Camel)		
	*Heliotropium cinerascens* Steud. ex A. DC.	Head injury	L	Topical	0.50	YA 009
		Arthritis	S	Topical insertion		
		Leprosy	L	Topical		
		Skin infection	L,R	Topical (Cow, goat, Camel)		
	*Heliotropium longiflorum* (A. DC.) Jaub. & Spach	White on eye	B	Topical	1.00	YA 049
Brassicaceae	*Lepidium sativum* L.	Somnambulism	F	Oral, body wash	0.67	YA 084
		Devil Disease	F	Oral		
		Dysentery	F	Oral, nasal		
Capparidaceae	*Boscia coriacea* Pax.	Retained Placenta	R	Tying	0.55	YA 016
		Leprosy	L	Topical		
		Lung Infection	L	Oral		
		Snake Bite	L,F	Oral, topical		
	*Cadaba farinosa* Forssk.	Eye sickness	L	Fumigation	0.22	YA 078
		Lung Infection	L	Sniffing, nasal		
		Mitch/Flue	L	Sniffing, nasal		
		Swelling on body	L	Topical, nasal		
		Head Injury	L	Topical		
		Breast cancer	L	Oral		
		Dyspepsia	L	Oral, nasal		
		Typhoid	L	Oral, nasal		
		Angina Pectoris	L	Oral		
		ETPB	S	Oral		
		Anthrax	R	Oral, nasal		
		Arthritis	R	Tying, topical		
		Impotence	R	Oral, tying		
		Infant sickness	L	Oral, nasal, body wash		
		Mastitis and contagious agalactia	L	Oral, nasal, ear, body wash (Cow, goat)		
		Anthrax	YP	Oral (Oxen)		
		Bovine pastuerollosis	L	Oral (Cattle)		
	*Cadaba glandulosa* Forssk.	Lung infection	L,S	Oral	0.33	YA 034
		Meningitis	L	Oral, body wash		
		Dyspepsia	L	Oral		
		Breast cancer	L	Oral, nasal, topical		
		Snake bite	L,R	Oral, nasal, topical		
		Jaundice	L	Oral, body wash		
		Tonsillitis	L	Oral, body wash		
		Lung infection	L,R	Oral		
		Typhoid	L	Oral, nasal		
		Swelling on skin	L	Oral, topical		
		Bovine pastuerollosis	L	Oral (Cattle)		
	*Cadaba rotundifolia* Forssk	Arthritis	S	Topical	0.19	YA 003
		Eye sickness	L	Fumigation		
		Arthritis	S	Topical insertion		
		Tonsillitis	L	Oral, nasal		
		Flue/Mitch	L	Inhaling		
		Retained Placenta	R	Oral		
		Broken head	L	Topical insertion		
		Snake Bite	L	Oral		
		Retained Placenta	R	Oral, nasal (Camel, Cattle)		
		External parasite	R	Topical (Cow, goat, sheep)		
		Brucellosis	WP	Oral (Goat, camel, cow)		
		ORF	UP	Oral (Goat, sheep)		
		CCPP	WP	Oral (Goat)		
		Bloating	R	Oral (Cattle)		
		Bovine TB	L	Oral, nasal (Cattle)		
	*Capparis decidua* (Forssk.) Edgew.	Anthrax	S	Oral (Cattle)	1.00	YA 085
Caryophyllaceae	*Silene macrosolen* Steud. ex A. Rich.*	Breast cancer	L	Oral	0.50	YA 104
		Diphtheria	L	Nasal		
		Brucellosis	L	Oral (Cow, goat)		
Convolvulaceae	*Seddera bagshawei* Rendle	Impotence	R	Nasal	0.40	YA 050
		Swelling	WP	Oral		
		Snake bite	L	Oral, nasal, body wash		
	*Seddera hirsute* Dammer ex Hall. f.	Malaria	L	Oral, nasal, body wash	0.40	YA 055
		Snake bite	S	Oral		
		Malaria	WP	Oral		
		Asthma	WP	Oral		
		Impotence	WP	Oral		
		Somnambulism	WP	Inhaling		
Cucurbitaceae	*Citrullus colocynthis* (L.) Schrad.*	Schizophrenia	L	Oral	0.50	YA 026
		Wart	S	Topical		
	*Citrullus lanatus* (Thunb.) Matsum. & Nakai *	Mastitis and contagious agalactia	L	Nasal, body wash (Goat, camel, cow)	1.00	YA 025
	*Cucumis prophetarum* L.	Swelling on body	WP	Oral	0.38	YA 028
		Unable to Urinate	WP	Oral		
		Eye injury	UP	Eye-drop		
		Devil Disease	YB	Oral, nasal, wash body		
		Skin infection	L	Oral, nasal (Cattle)		
Euphorbiaceae	*Acalypha fruticosa* Forssk.	Eye sickness	L	Fumigation	0.15	YA 086
		Tonsillitis	L	Oral, nasal		
		Lung Infection	L	Inhaling		
		Infant sickness	L	Oral, nasal, ear		
		Breast cancer	L	Oral, nasal, topical		
		Kola kusil	L	Oral, topical		
		Epilepsy	L	Oral		
		ETPB	L	Oral, wash body		
		Bone breakage	L	Tying/topical		
		Dyspepsia	L	Oral, nasal		
		Herpes Zoster	L	Oral, nasal		
		Schizophrenia	L	Oral		
		Blackleg	L	Oral, nasal (Camel, cattle, goat)		
		Bovine pasteurellosis	L	Nasal, oral (Cattle)		
		Anthrax	L	Nasal, oral (Camel, cattle, goat)		
		CCPP	L	Oral, nasal (Camel, cattle, goat)		
		Bone breakage/ fractures	L	Topical (cattle, goat)		
		Brucellosis	L	Oral (Cow, goat)		
		Pastuerollosis	L	Oral (Cattle)		
		PPR	L	Oral (Cattle)		
	*Acalypha indica* L.	Malaria, chill	WP	Oral	0.50	YA 019
		Snake bite	WP	Oral, nasal, body wash		
		Jaundice	L	Oral		
		Dyspepsia	L	Oral, nasal		
		Breast infection	L	Oral, nasal, ear		
		Lung Infection	L	Oral		
		Herpes Zoster	L	Oral, nasal		
		Snake Bite	L	Oral, nasal (Cow, camel, goat)		
		Brucellosis	L	Oral, nasal, ear, body wash (Cow, camel, goat)		
		PPR	L	Oral (cattle)		
	*Euphorbia triaculeata* Forssk.	Jaundice	L	Nasal, wash body	0.67	YA 064
		STIs	SR	Oral		
		Dyspepsia	L, La	Oral		
		Dysentery	L	Nasal, oral (Goat)		
		Orf	L	Oral, nasal (Goat)		
Fabaceae	*Acacia ehrenbergiana* Hayne	Dyspepsia	B	Oral	1.00	YA 074
	*Acacia mellifera* (M. Vahl) Benth.	Flue	L/B	Sniffing, Oral	0.47	YA 075
		Fire-burn	S	Topical		
		Head/ bullet Injury	B,S	Tying, topical		
		Eye injury	L	Eye-drop		
		EPTB	L	Oral		
		Birth labour	YB	Oral, body wash		
		External infection	S	Topical (camel)		
		PPR	L	Eye-drop (cattle)		
	*Acacia nilotica* (L.)	Retained placenta	L,R	Oral, nasal	0.75	YA 041
		Broken head	Br	Topical		
	*Acacia oerfota* (Forssk.) Schweinf.	Arthritis	S,F	Topical	0.35	YA 045
		Diphtheria	L	Oral, nasal, body wash		
		STIs	R, B	Oral, body wash		
		Breast cancer	L	Eye, nasal, topical		
		Dyspepsia	L	Nasal, body wash		
		Flue, coughing	B	Oral		
		Devil Disease	R,L	Oral, nasal, body wash		
		Scabies	L	Topical		
		Sheep and Goat pox	L	Oral (Sheep, goat)		
		Mastitis and contagious agalactia	L	Nasal, body wash (cow, goat, camel)		
		Bovine TB	R	Oral, nasal (cattle)		
	*Acacia senegal* (L.) Wild	Mumps	L	Topical, nasal	0.67	YA 091
		Eye injury	B	Eye-drop		
		Impotence	R	Oral, topical		
	*Acacia seyal* Del.	Intestinal parasite	R	Oral	0.67	YA 039
		Jaundice	L	Oral		
	*Acacia tortilis* (Forssk.) Hayne.	External injury	L	Topical	0.50	YA 035
		Infant sickness	L	Body wash		
		Breast cancer	L	Oral. nasal, topical		
		Brucellosis	L	Oral (Cow, goat)		
	*Dichrostachys cinerea* (L.) Wight et Am.	Skin bleaching (cancer)	R	Oral	0.67	YA 037
		Foot and mouth	B	Topical insertion (Cattle, goat, sheep)		
	*Indigofera articulata* Gouan	Jaundice	R	Oral	0.25	YA 015
		Epilepsy	L,R	Oral		
		Fire burn	L	Topical		
		Dyspepsia	L,S,R	Oral		
		Epistaxis	L,R	Nasal, oral		
		Snake Bite	R	Oral		
		Swelling on body	L	Oral		
		Brucellosis	L	Oral (Cow, goat)		
	*Indigofera oblongifolia* Forsk.	Diphtheria	UP	Oral, nasal, ear, body wash	0.45	YA 013
		Typhoid	L,S	Oral		
		Herpes Zoster	R	Oral, ear, mouth wash		
		Scorpion bite	Root	Oral, tying		
		Devil illness	WP	Body wash		
		Lung infection	L,R	Oral		
		Dysentery	L,R	Oral		
		Breast cancer	Leaf	Oral and body wash		
		Angina	R	Oral		
		Foot and mouth	GP	Topical insertion (Cattle)		
		Pastuerollosis	L	Oral (Cattle)		
		PPR	L	Oral (Cattle)		
	*Indigofera spicata* Forsk.	Jaundice	R	Nasal, body wash	1.00	YA 070
	*Parkinsonia scioana* (Chiov.) Brenan	Broken bone	B,R	Tying, topical	0.67	YA 081
		Dyspepsia	B	Oral		
	*Senna alexandrina* Mill.	Circumcise infection	La	Topical	0.30	YA 082
		Dyspepsia	WP	Oral		
		Infant sickness	L	Nasal		
		Snake bite	L	Oral, nasal, topical		
		Devil illness	L	Body wash		
		Jaundice	B	Oral		
		External injury	L	Topical		
		Swelling on body	L	Topical		
	*Senna italica* Mill.	Breast cancer	L	Oral, nasal, body wash	0.50	YA 083
		Devil illness	L	Body wash		
		Mumps	R,L	Oral, nasal		
		Dyspepsia	L	Oral, nasal		
		Retained placenta	S	Oral (Camel)		
	*Tamarindus indica* L.	Lung infection	B	Oral, nasal	0.50	YA 063
		Typhoid	B	Oral		
	*Trigonella foenum-graecum* L.*	Epistaxis	R	Nasal	0.50	YA 017
		Infant Sickness	S	Body wash		
Lamiaceae	*Becium filamentosum* (Forssk.)	Malaria	L	Oral	0.55	YA 033
		Lung Infection	L,S	Oral		
		Dyspepsia	L	Oral		
		Somnambulism	L	Oral, wash body		
		Syphilis	F	Oral, nasal		
		Birth labour	Br	Wash body		
		Eye infection	L	Topical		
		PPR	L	Oral (Cattle)		
	*Ocimum basilicum* L.*	Internal parasites	L	Oral	0.50	YA 106
		Swelling on skin	WP	Topical		
	*Ocimum spicatum* Defl.*	Schizophrenia	R	Oral, nasal	0.67	YA 018
		Snake Bite	R	Oral		
	*Ocimum urticifolium* Roth.	Infant dysentery	L	Oral, nasal, body wash	0.50	YA 001
		Diphtheria	WP	Oral, nasal, body wash		
		Malaria	WP	Oral, nasal, body wash		
		Dyspepsia	UP	Oral		
		Herpes Zoster	L	Nasal		
	*Orthosiphon pallidus* Royle ex Benth.*	Diphtheria	L	Oral, nasal, body wash	1.00	YA 101
	*Thymus schimperi* Ronniger*	Snake bite	WP	Oral, body wash	0.50	YA 054
		Devil Disease	S	Oral, body wash		
		Internal parasites	R	Oral		
Loranthaceae	*Oncocalyx glabratus* (Engl.) M. Gilbert*	Bullet injury	S,L	Topical	1.00	YA 105
	*Oncocalyx schimperi* (A. Rich.) M. G. Gilbert	Jaundice	L	Oral	0.50	YA 029
	*Plicosepalus robustus* Wiens & Polhill*	Herpes Zoster	WP	Oral, nasal, body wash	0.75	YA 076
	*Tapinanthus globiferus* (A. Rich.) Tieghem*	Dyspepsia	L	Oral, ear, mouth wash	0.50	YA 079
		Impotence	L	Oral		
Lythraceae	*Lawsonia inermis* L.*	Elephantiasis	L	Tying, topical	1.00	YA 059
Malvaceae	*Abutilon figarianum* Guill. & Perro	Eye infection	R	Fumigation	0.50	YA 052
		Fire, swelling	L	Topical, oral		
		Flue	L	Chewing, sniffing		
	*Hibiscus vitifolius* L.	Angina Pectoris	R	Tying, topical	1.00	YA 047
Menispermaceae	*Cocculus pendulus* (J. R. & G. Forst) Diels*	Somnambulism	L	Oral, wash body	0.29	YA 053
		Retained Placenta	L	Oral		
		Elephantiasis	S	Topical insertion		
		Snake Bite	R	Oral		
		Breast infection	R	Topical		
Moraceae	*Dorstenia barnimiana* Schweinf.*	Schizophrenia	WP	Inhaling	1.00	YA 088
Moringaceae	*Moringa oleifera* Lam.	Snake Bite	R,B	Oral, tying	1.00	YA 032
Nyctaginaceae	*Commicarpus helenae* (J.A. Schultes) Meikle	Breast infection	L	Nasal, body	0.50	YA 066
		Vomiting	L	Oral		
		Elephantiasis	L	Topical		
		Typhoid	WP	Oral, nasal		
		Herpes Zoster	R	Nasal		
		Mastitis and contagious agalactia	L	Nasal, body wash (Cow, goat, Camel)		
	*Commicarpus squarrosus* (Heimerl) Standl.*	Trypanosomiasis	S	Nasal, ear, body wash (Camel)	1.00	YA 056
Oleaceae	*Olea europaea L. subsp. cuspidata* (Wall.ex G. Don) Cif.	Snake Bite	Stem	Oral, topical	1.00	YA 102
Plumbaginaceae	*Plumbago zeylanica* L.	Meningitis	L	Oral, nasal	0.75	YA 006
		Scabies	L,R	Topical		
		Skin infection	L	Oral, topical		
Poaceae	*Cymbopogon commutatus* (Steud.) Stapf	Jaundice	Tu	Oral, wash body	0.50	YA 005
		Eye Infection	L	Eye-drop		
Polygalacea	*Polygala obtusissima* Hochst. Ex Chod.	Flue	WP	Inhaling	0.50	YA 051
		Dyspepsia	R	Oral		
		Asthma	R	Oral		
Ranunculaceae	*Nigella sativa* L.*	Somnambulism	S	Oral, body wash	1.00	YA 096
Rhamnaceae	*Ziziphus mauritiana* Lam.	Retained placenta	L	Oral	0.50	YA 068
		Breast cancer	L	Oral		
		Brucellosis	L	Oral (Cow, goat)		
	*Ziziphus spina-christi* (L.) Desf.	Retained placenta	L	Oral	0.43	YA 069
		Snake bite	YB	Oral		
		Angina Pectoris	Root	Oral		
		Dyspepsia	YB	Oral		
Rutaceae	*Citrus lemon* (L.) Bunn.f.	Somnambulism	F	Oral, wash body	1.00	YA 071
Salvadoraceae	*Dobera glabra* (Forssk.) Poir.	Head Wound	B,L	Topical	0.43	YA 042
		ETPB	L	Oral		
		Anthrax	L	Oral (Cow, oxen)		
		Bloating	R	Oral (Cattle)		
		Blackleg	R,L	Oral (Cattle)		
		Skin infection	L	Topical (Cattle, camel)		
Sapotaceae	*Mimusops kummel* Bruce A. DC.	Dyspepsia	R	Oral	1.00	YA 038
Selaginellaceae	*Selaginella kraussiana* (Kunze) A.Braun*	Fire burn	WP	Topical	0.67	YA 067
		Anthrax	UP	Nasal, oral (cattle, goat, sheep)		
Solanaceae	*Capsicum annuum* L.	Arthritis	S	Topical insertion	1.00	YA 060
		Anthrax	S	Oral (Cattle)		
	*Capsicum frutescens* L.	Pasteurellosis	Fr	Nasal, oral (Camel)	0.50	YA 061
		Flue	Fr	Nasal, oral (Camel)		
	*Solanum incanum* L.	Schizophrenia	UP	Oral, inhaling	0.50	YA 100
		Blackleg	R	Oral, nasal, topical (Cattle)		
		CCPP	R	Oral, nasal (Goat)		
		Lung infection	R	Oral, nasal (Camel)		
	*Solanum marginatum* L. f.	Schizophrenia	L,R	Oral	0.29	YA 097
		Mumps	L	Oral		
		Meningitis	L	Oral		
		Head injury	L	Topical		
		Dyspepsia	L	Oral		
		Pastuerollosis	L	Oral (Cattle)		
	*Solanum somalense* Franchet.	Fire Burn	UP	Topical	0.67	YA 036
		Typhoid	UP	Nasal		
	*Withania somnifera* (L.) Dunal	Typhoid	R	Oral	0.57	YA 040
		Evil eye	R	Oral, inhaling		
		Swelling on skin	L	Oral, topical		
		ETPB	L	Oral		
Sterculiaceae	*Sterculia africana* (Lour.) Fiori*	Infant sickness	L	Oral	0.50	YA 090
		Swelling on skin	Leaf	Topical		
Tiliaceae	*Grewia erythraea* Schweinf.	Head wound	S,B	Topical	0.36	YA 062
		Flue	L, B	Inhaling		
		Typhoid	L	Oral		
		Broken bone	R	Topical		
		Dyspepsia	L,S,B	Oral, nasal		
		Arthritis	R	Topical insertion		
		Wart	B	Topical		
		Infant Sickness	L,R	Nasal, body wash		
		Leprosy	R	Topical		
	*Grewia villosa*	Broken bone	R	Tying, topical	0.40	YA 048
		Impotence	R	Oral, body wash		
		Jaundice	WP	Oral, nasal		
		Foot and mouth	S	Nasal, oral (Cattle, goat, sheep)		
Verbenaceae	*Premna oligotricha* Baker*	Retained placenta	L	Oral	1.00	YA 023
	*Priva curtisiae* Kobuski*	Typhoid, Mitch	L	Oral, nasal	1.00	YA 099
Vitaceae	*Cissus quadrangularis* L.	Leprosy	YB	Oral, topical	0.33	YA 044
		ETPB, lung infection	YB	Oral, topical		
		Swelling on neck, chest	YP	Oral, nasal (Cattle)		
	*Cissus rotundifolia* (Forssk.) Vahl	Devil Disease	RL	Inhaling	0.50	YA 080
	*Cyphostemma burgeri* Vollesen*	Snake Bite	R	Oral	0.50	YA 007
		Hand swelling	L	Topical		
Zygophyllaceae	*Fagonia paulayana* Wagner & Vierh.*	Infant sickness	UP	Oral, Nasal	1.00	YA 031
	*Fagonia schweinfurthii* Hadidi	Tonsillitis	R	Oral	0.50	YA 010
		Jaundice	WP	Oral, body wash		
		Infant sickness	L	Oral, body wash		
		Lung infection	R	Oral, body wash		
		Mastitis and contagious agalactia	WP	Nasal, body wash (Cow, goat, camel)		

*B* bark, *C* climber, *F* flower, *Fr* fruit, *L* leaf, *La* latex, *R* root, *S* stem, *Br* branch, *UP* upper part, *WP* whole plant, *YP* young plant

*new reports in Afar region

### Ethnoveterinary medicinal plants

Thirty-nine of the plant species were used to treat human as well as livestock diseases, but *Capparis decidua* (Forssk.) Edgew., *Capsicum frutescens* L., and *Commicarpus squarrosus* (Heimerl) Standl. were only used in the treatment of livestock. The plants were distributed into 21 Family and 42 genera. Fabaceae was represented by six species followed by Capparidaceae with four species, Boraginaceae, Euphorbiaceae, Solanaceae with three species each, and Aloaceae, Amaranthaceae, Apocynaceae, Balanitaceae, Cucurbitaceae, Nyctaginaceae with two species each and the rest with one species each (Table 1; Additional file 2).

### Habit and parts of medicinal plants

**Fig. 3:**
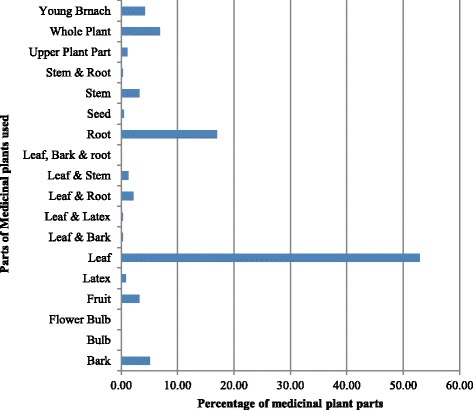
Parts of plants used for the preparation of remedies

The majority of medicinal plants were shrubs (44%), followed by herbs (28%), trees (21%), and climbers (7%). The leaf (52.94%) was used in the majority of the remedy preparations followed by root (16.99%), whole plant (6.86%), bark (5.07%), young branch (4.25%), fruit (3.27%) and stem (3.27%) (Fig. 2). The remedies were prepared from fresh (95%), dry (4%) and either fresh or dry (1%) parts. Few remedy preparation from seed (67%), whole plant (19%), fruit (15%), and bark (13%) were from dried parts whereas the majority of the remedies were prepared from fresh plant parts (Fig. 3).

### Preparation, dosage, and mode of treatment

The majority of the treatments were prepared from a single plant (80%) and some from a mixture of multiple plants (20%). The health conditions treated with a mixture of plants were coughing blood through mouth and nostrils; knee sickness and swelling; breast swelling and infection; infant illness and fever; sleepwalking; epilepsy; devil illness; sudden illness, fever, dysentery, and vomiting; witchcraft disease; bovine brucellosis; and pasteurellosis. Forty-eight plants were used in multiple plant preparation. The most popular plants in the preparations were *A. fruticosa* (40%), *A. tortilis* (25%), *B. rotundifolia* (25%), *Indigofera oblongifolia* Forsk. (17%), *C. farinosa* (15%), *Celosia polystachia* (Forssk.) C.C. Towns. (13%), *Becium filamentosum* (Forssk.) Chiov. (10%) and *C. rotundifolia* (10%) (Table 1; Aditional Files 1 and 2).

**Fig. 4:**
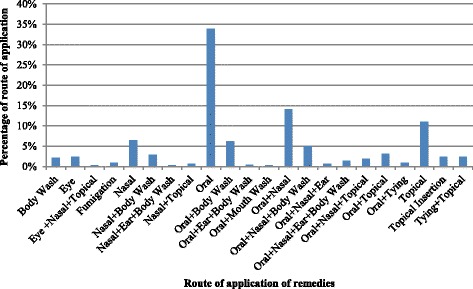
Routes of application of treatments

The primary methods of preparation of remedies were crushing and pounding. The crushing and pounding were done using two stones one flat shaped and the other oval or spherical shaped to fit into the hands. The diluents were water, the blood of a black goat, and milk of goat or camel. The oral (68%) was the major route of administration of treatments followed by topical (16%), nasal (10%), eye (3%) and body wash (2%) (Fig. 4). The filtrate was applied orally, through nostril, and as an ear and eye drop. The residue was used for body washing. Camel’s milk was given as an antidote for remedies that upset or cause irritation of the stomach and honey was added to those preparations with a bitter taste. Treatments applied topically were charred, ground and the powder was mixed with butter to make a paste. The parts of medicinal plants that were inserted into a cut made in the knee, breast, or swollen body parts were mixed with salt, pepper, or butter. Fresh plants were chewed, and the juice was swallowed before a meal for a day. The remedies for some diseases were boiled in the evening, and the filtrate was taken before breakfast in the morning. Some diseases were treated with a combination of routes in both human and livestock treatments (Table 1; Additional files 1 & 2).

The dosages taken orally were measured using a small coffee cup (~5 ml), glass (~20 ml), and tin can (~50 ml) for humans and large tin can (~3lt) for animals. The coffee cup was used to provide remedies to a child and tin cans for adults based on the symptoms and physical condition of the patients. The medication applied as eyedrops, eardrops and through nostrils was five to ten drops while in the treatment of animals thin bamboo stem about ten centimeters long was used. The application of remedies for flu, sleepwalking, and devil illness was inhaling the smoke, fume, and steam the patient is covered by overcoat/blanket or sniffing of crushed or powdered fresh plant parts wrapped with locally weaved close. Sniffing was not administered to children under age of five (Additional files 1 and 2). The majority of the treatments were given only once in the morning as a single dose or three times in a day or for two to seven days. However, some treatments were given only once for all animals as a vaccine to prevent and stop the transmission of the contagious disease such as CBPP, anthrax, blackleg, and bovine pasteurellosis (Additional file 2).

### Fidelity value and informants consensus factor

The medicinal plants reported by few informants (one to two) or that were used only as a treatment for an ailment had FL value of 1.00. *Dorstenia barnimiana* Schweinf. was used as a treatment for schizophrenia, and *Priva curtisiae* Kobuski as a treatment for typhoid/typhus, headache, and fever and had FL value of 1.00 (Table 1). On the other hand, medicinal plants that were used to treat a variety of ailments had lower FL values. *Indigofera articulata* (0.25), *C. farinosa* (0.22), *C. rotundifolia* (0.19), and *A. fruticosa* (0.15) were used to treat diseases ranging from human to livestock and were reported by more than 20 informants.

The informant consensus factor was calculated by pulling to gather the human and animal ailments into ten categories. The values obtained were in agreement with the top ten causes of morbidity and mortality in the Yalo Woreda. Malaria and febrile diseases had the highest (ICF = 0.69) value followed by internal and liver infection (0.67), and gastrointestinal disease and internal parasite (0.62) (Table 2).

**Table 2:** Informant consensus value of category of diseases of public health and livestock importance in Yalow Woreda

Category	Species	(%) All Species	Use citations	(%) All use citations	ICF
Acute febrile illness and malaria	32	31%	102	18%	0.69
Internal and liver infection	55	54%	166	29%	0.67
Gastrointestinal disease, internal parasite	50	49%	129	23%	0.62
Swellings and cancer	45	44%	102	18%	0.56
Flue, headache	25	25%	54	10%	0.55
Eye, ear nose, and mouth	35	34%	63	11%	0.45
Respiratory and lung infection	26	25%	41	7%	0.38
External injury and external parasite	28	27%	44	8%	0.37
Skin and subcutaneous tissue infection	30	29%	43	8%	0.31
Snake and scorpion bite	20	20%	28	5%	0.30
Devil disease, schizophrenia, epilepsy	25	25%	34	6%	0.27
Birth related and urinary tract infection.	18	18%	23	4%	0.23
Impotence	6	6%	7	1%	0.17

### Preference ranking

The preference ranking of medicinal plants reported by 15 and more informants and used as a remedy for multiple diseases is shown in Table 3. The medicinal plants were ranked based on their healing potential of a disease. *Balanites rotundifolia* and *A. fruticosa* were used as remedies for diseases such as breast cancer, dyspepsia, epilepsy, ETPB, eye sickness, herpes zoster, infant disease, jaundice, lung infection, and malaria. *Cadaba glandulosa* Forssk. and *Celosia polystachia* (Forssk.) C.C. Townsend had the lowest two ranks and used as a treatment for stomach ache, bloody dysentery, fever and sudden illness. (Additional files 1 and 2).

**Table 3:** Preference ranking of medicinal plants predominantly cited as remedies for humans and animal in Yalo Woreda

Plants used in treatment of multiple diseases	R1	R2	R3	R4	R5	R6	R7	R8	Total	Rank
*Acacia mellifera*	6	8	6	7	7	8	7	8	57	9
*Acacia oerfota*	7	7	8	6	8	7	8	7	58	8
*Acalypha fruticosa*	9	9	8	9	9	8	9	8	69	2
*Acalypha indica*	8	8	9	8	8	9	8	9	67	3
*Balanites aegyptiaca*	8	7	8	9	8	8	7	8	63	5
*Balanites rotundifolia*	9	8	9	9	8	9	9	9	70	1
*Cadaba farinosa*	7	7	8	9	8	8	8	9	64	4
*Cadaba glandulosa*	7	6	8	7	6	6	7	8	55	10
*Cadaba rotundifolia*	8	7	8	8	8	7	8	7	61	7
*Celosia polystachia*	6	7	7	8	7	6	6	7	54	11
*Indigofera oblongifolia*	7	6	7	8	9	7	9	9	62	6

### Edible plants and duration of gathering

Informants reported 16 edible plants and 56% of these plant species were trees and 44% were shrubs. Fourteen of the plants were nutraceutical; *Rosa abyssinica* R. Br. and *Ximenia americana* L. were used only as edible plants. All edible fruits were eaten raw (Table 4). The duration of gathering and consumption were dependent on the availability of edible parts and seasons. *Boscia coriacea* Pax.*, Carissa spinarum* L., *Dobera glabra* (Forssk.) Poir., *Mimusops kummel* A. DC.*, R. abyssinica, Tamarindus indica* L., and *Z. spina-christi* were gathered in Gilal as food supplements. *Balanites aegyptiaca, B. rotundifolia,* and *Cordia sinensis* Lam. were collected in Hagay as an alternative food. *Grewia bicolor* Juss., *Grewia erythraea* Schweinf., *Rhus natalensis* Krauss, *Ruellia patula* Jacq.*,* and *X. americana* were gathered in Sugum-Karma and used as dietary supplements *Balanites aegyptiaca, B. rotundifolia, C.sinensis*, and *D. glabra* were sold in open markets and had economic importance.

**Table 4:** Edible plants consumed by Afar people in Yalow Woreda

Voucher Number	Family	Scientific Name (Citations)	Habit	Local plant name	Mode of consumption
YA 021	Acanthaceae	*Ruellia patula* Jacq.	Shrub	Boboyta	The outer cover is eaten fresh. It is collected and consumed by herd boys, and it is not stored and has a mild test.
YA 012	Anacardiaceae	*Rhus natalensis* Krauss	Tree	Dewa/ Sofa	Whole fruit, after trashing using both hands, is eaten fresh. It is sweet and consumed by all age groups. It collected and mostly consumed by herd boys
YA 092	Apocynaceae	*Carissa spinarum* L.	Shrub	Titita	It has sweet to sour test, collected and consumed by all age groups
YA 095	Balanitaceae	*Balanites aegyptiaca* (van Tieghem) Blatter	Tree	Udayto	The outer cover is eaten fresh, and the inside is eaten boiled and can be stored. It is collected by housewives, herd boys, and men and consumed by all age groups. It has a mild test and stored for a drought period.
YA 008	Balanitaceae	*Balanites rotundifolia* (Van Tiegn.) Blatter	Shrub Small tree	Alayto	The outer part is eaten fresh. The internal part is bitter, hence, soaked in water overnight and followed by two to three times washing, boiled and consumed by all age groups. Housewives and herd boys collect and stored for drought period as a food source. It is also consumed fresh by diabetic patients.
YA 072	Boraginaceae	*Cordia sinensis* Lam.	Tree	Madera/ Ledo	It is collected by boys and housewives. It is sweet as honey and has more fat. It is preferably given to children under five years and women that have given birth. It is eaten before breakfast and dinner.
YA 016	Capparidaceae	*Boscia coriacea* Pax.	Tree	Homura/ Aytneba	It too sour and only one crushed fruit is taken for smoothening taste by older individuals that take milk as food for a longer period. It is used as medicine to avoid dyspepsia.
YA 063	Fabaceae	*Tamarindus indica* L.	Tree	Hura	It has a mild taste between sour, and bitter. It is collected by herd boys and housewives and eaten fresh. The whole fruit is chewed, and the seeds are spat.
YA 058	Olacaceae	*Ximenia americana* L.	Tree	Helelea	It is sweet, succulent fruit collected by herd boys and consumed by all age groups.
YA 069	Rhamnaceae	*Ziziphus spina-christi* (L.) Desf.	Tree	Kusra	It collected by herd boys and housewives and eaten fresh by all age groups.
YA 011	Rosaceae	*Rosa abyssinica* Lindley	Shrub	Atim	It is sweet and collected and consumed by all age groups. The whole fruit is eaten fresh, and only seeds are spat.
YA 042	Salvadoraceae	*Dobera glabra* (Forssk.) Poir.	Shrub	Gasera/ Mudua/	The outer part is eaten fresh and inner part is taken as food boiled mixed with Alayto during drought season. It collected by housewives and herd boys, stored for drought season, and consumed by all age groups.
YA 038	Sapotaceae	*Mimusops kummel* A. DC.	Tree	Yelow Eta	Unripe fruit is collected by boys and buried in the soil until it ripens and becomes red. The whole fruit is consumed by all age groups. Unripe fruit is not edible and assumed to be poisonous.
YA 057	Tiliaceae	*Grewia bicolor* Juss.	Tree	Hebele	It is collected by herd boys, and whole fruit is consumed after the hairy outer part is cleaned. It is sweet and eaten by all age groups
YA 062	Tiliaceae	*Grewia erythraea* Schweinf.	Shrub	Hidayto	It is sweet fruit collected by herd boys and consumed by all age groups and is not stored
YA 048	Tiliaceae	*Grewia villosa* Willd.	Shrub	Habeleyta	It is collected by herd boys and consumed fresh by all age groups.

## Discussion

### Traditional knowledge and medicinal plants of human and veterinary importance

The number and diversity of medicinal plants reported by informants show the rich traditional knowledge in Yalo Woreda and the number of medicinal plants reported is more than the studies conducted in Afar and neighboring regions [10, 17, 22, 30–34]. The transference of traditional knowledge in the study area is from male parent to their sons since females have low status in the society and do not inherit property on the same basis to the male [4, 6]. The study conducted in Namibia indicated similar practice [35]; the females learn by routine observation whereas the boys are taught by their parents besides regular observation [22, 36–39]. The vertical transference of TK from father to son is a common phenomenon in Ethiopia, Africa, and Asia [17, 22, 35, 40–48]. However, studies conducted on knowledge of medicinal plants in Ankober district, Samburu district, Kenya and in South America show no difference in the average number of plants reported either by female or male respondents [47, 49, 50]. The division of labor in the society has determined the TK difference between female and male. The female informants in Yalo woreda reported only three plants with veterinary importance since the males are the sole livestock herders in the region and responsible for the animals’ wellbeing [37–39, 44]. The ethnoveterinary knowledge in pastoral society is acquired from their parents during grazing that indicates females’ knowledge of ethnoveterinary medicinal plants is less than men, which is similar to results reported by other studies on important ethnoveterinary plant [44, 51–54].

The number of medicinal plants reported in the study area increased with age, and the older informants reported more medicinal plants than the younger individual [36, 50, 52, 55]. Abera [56] has shown in the study conducted, in Ghimbi District, on Oromo people that the young generation is losing the interest in using medicinal plants because of changes induced by development and abandons of rural life. This phenomenon leads to the disappearance of associated traditional knowledge and interrupts transference of knowledge to next generations [16, 35, 47, 57–61]. Similarly, the study conducted, in the semi-arid region of Brazil, has shown that age and income difference had implications on knowledge of informants and correlated to some plants that have an effect on the traditional knowledge of younger generation [40, 50]. The transference of knowledge in the Afar family is similar to most of the pastoral areas where youngsters learn knowledge of medicinal plants from the elders during grazing. A child in Maasai pastoralists’ society in Eastern Africa has to learn and identify grasses and plants with medicinal importance in their area during grazing. The boys are taught in the field and at home, though girls are taught by their mothers and grandmothers only at home [62].

The religious establishments in Mesgid and Rekrek Kebeles and other cultural aspects had no association with traditional knowledge of medicinal plants in Yalo Wereda [63]. Though, Abu-Rabia [64] reported that knowledge of plants of nomadic pastoralists in Middle East countries originated from the sayings of Prophet Muhammad’s on health and illness: ‘*The Medicine of the Prophet*”. Also, the study conducted around Debre Libanos monastery has shown that the knowledge of the people varies based on the distance from the Monastery since religion is one of the aspects of variation in TK among the different societies in Ethiopia [45, 63, 65–68].

The medicinal plants reported by the people in Yalo Woreda are also reported by ethnobotanical studies conducted in semi-arid and arid regions in Ethiopia. Most of the medicinal plants are drought resistant, and many of them are a member of *Acacia-Commiphora* Woodland [1, 10, 17, 21, 22, 41, 46, 68]. The medicinal plants were collected from natural vegetation since the majority (95%) of the population is pastoralists, and the rest are urban dwellers without a home garden. In Ethiopia, the majority of the traditional medicines are prepared from plants collected from the wild [9, 53, 69–74]. The culture of conservation of medicinal plants in the home garden is not practiced in many regions of Ethiopia. Conservation of the medicinal plants is a requirement since the recurrent drought imposed by climatic changes has an impact on the natural vegetation. The other threat to the vegetation of the study area is the spread of invasive species such as *P. juliflora* that are replacing the plants with cultural values and changing vegetation to monotype bushes and forests. The people in Djibouti and Borena pastoralists areas reported a similar scenario where encroachment of invasive plants has resulted in a loss of valuable plants and degradation of the rangeland that indicates the necessity of further additional ethnobotanical studies in Afar region [1, 9, 15–18, 32, 57, 60, 75–78].

The medicinal plants reported in Yalo Woreda are also reported by other studies conducted in Afar and other areas where the Afar people are inhabitants. Bahru et al. [10] reported fourteen plant species and Beche et al. [34] reported ten plant species in the studies conducted around the Awash National Park. Meragiaw [23] reported twelve plant species in a study carried out in Aba’ala Woreda; Seifu et al. [22] reported nine plant species in a study conducted in Chifra District; and Belayneh et al. [41] reported 14 plant species in a study carried out in pastoral and agro-pastoral communities in Erer Valley of Babile Woreda. The survey conducted in the region of Randa, Djibouti reported 46 plant species [17]. The studies commonly reported *Acacia mellifera* (M. Vahl) Benth.*,* .*A. oerfota,* .*A. tortilis, B. aegyptiaca, Balanites rotundifolia* (van Tiegn.) Blatter*, Cadaba farinosa* Forssk.*, Cadaba rotundifolia* Forssk*, Cissus quadrangularis* L.*, Indigofera articulata* Gouan*, Olea europaea L. subsp. cuspidata* (Wall.ex G. Don) Cif.*, Solanum incanum* L.*, Withania somnifera* (L.) Dunal*, Ziziphus spina-christi* (L.) Desf. The similarity in the naming of plants by the individuals in these study areas indicates similarity in their cultural and traditional practices. Furthermore, the use of the plants in broad geographic regions adds value to the therapeutic potential of the medicinal plants in Yalo Woreda [12, 17, 23, 34, 43, 69]. However, 29 medicinal plants are new reports by Yalo Woreda informants compared to other studies conducted in Afar region, which are used as a treatment for different health conditions in Ethiopia and elsewhere. [10, 14, 16, 32, 35, 41, 47, 48, 50, 56, 60, 62, 66, 69, 71, 76, 78–89]. Nevertheless, some of the plants are not documented in the reviewed ethnobotanical studies in Ethiopia. *Citrullus lanatus* (Thunb.) Matsum. & Nakai*, Cyphostemma burgeri* Vollesen, *Dorstenia barnimiana* Schweinf. that are used as a treatment for various diseases in Yalo Woreda are edible plants in other parts of Ethiopia [81, 90–92]. *Fagonia paulayana* Wagner & Vierh. used as a remedy for infant sickness in Yalo Woreda has a similar purpose to other *Fagonia* spp. reported in Pakistan [93, 94]. *Plicosepalus robustus* Wiens & Polhill is a parasitic plant that grows on *Cadaba farinosa* Forssk., and used as a treatment for an infection on skin and mouth, and tooth decay has a similar effect as *Plicosepalus curviflorus* Tiegh. and *Plicosepalus acaciae* Zucc. [95–97] with high activity against bacterial infections, and *Plicosepalus nummulariifolius* (Franch.)Wiens& Polhill. used as a treatment to gastritis in Djibouti [17]. *Priva curtisiae* Kobuski used as a treatment for typhoid, headache, and fever in Yalo Woreda has similar medicinal uses to species within the same genus; *Priva cordifolia* Druce, *Priva flabelliformis* (Mold.) R. Fernand and *Priva lappulacea* (L.) Pers. used as a treatment for different health conditions in Uganda and Martinique [98, 99]. *Selaginella kraussiana* (Kunze) A.Braun is used to treat sickness related to burning and wound; studies conducted on another species of Selaginella reported as a treatment to wound, anticancer and antimalarial in different parts of the world [100–102].

Fabaceae are major plant species reported as remedies to treat livestock diseases similar to other studies in the country [17,22,34.41,44,58,69,85], but in the study conducted in Ada’ar District, Afar regional state, Asclepiadaceae and Capparidaceae are dominant plant species reported as a treatment to livestock [9]. The ethnoveterinary plants reported in the current study such as *A. oerfota*, *Acalypha fruticosa* Forssk., *B. aegyptiacus*, *Calotropis procera* (Ait.) Ait.f., *C. frutescens*, *C. quadrangularis* and *S. incanum*. Are utilized by other societies as a treatment for varies type of animal health conditions [9, 13, 17, 20, 22, 25, 35, 41, 44, 51, 55, 71, 77, 79, 81]. Giday and Teklehaymanot [9] reported seventeen plant species in Ada’ar District, Afar Regional State; Gradé [58] reported seventeen plant species in pastoral Karamoja, Northern Uganda; Dharani [103] reported 10 plant species in East Africa and Sori [104] reported ten plant species in Borana Pastoralists, Southern Ethiopia [9, 13, 17, 20, 22, 25, 35, 41, 44, 51, 55, 69, 71, 79, 81]. The number of ethnoveterinary plants reported in Yalo woreda is more than many studies conducted in Ethiopia [30, 53, 55, 60, 70, 72–74, 87, 104–109] even though studies undertaken in some societies reported a higher number of ethnoveterinary plants [9, 43, 77, 104, 110, 111]. It indicates the rich knowledge of ethnoveterinary important plants in the study area since the people are highly dependent on the animals for their living [43, 58, 60, 69, 70, 104]. The Afar people seasonally migrates in search of grazing and water for their animals and utilize plants from the natural vegetation to treat animal’s illness through trial and error that profoundly contributed to the knowledge of individual herders. The plants employed for treating a disease are diverse, which is the outcome of experience gained by the various informants during grazing. The individual herder is responsible for the well-being of the livestock and uses plants found in the grazing area in addition to the knowledge passed from their parents to manage animal health conditions [9, 54, 58, 112].

Most of the medicinal plants used for the preparation of remedies were shrubs and trees that are available throughout the year. The shrubs and trees are dominantly used for the preparation of medications in most sub-arid and arid regions since the plants survive and are available in dry seasons. Trees and shrubs are used in the preparation of medications in pastoralists area such as in Borena pastoralists, 56% of remedies are prepared from trees and shrubs, and in Ada’ar District, Afar Reginal state 67.3% of ethnoveterinary plants are shrubs [9, 34, 41, 43, 48, 68, 72, 113, 120, 123]. In Yalow Woreda, the majority of remedies are prepared from fresh leaves; similar to the reports by most of the ethnobotanical studies conducted in Ethiopia and elsewhere [9,34,35 43 53,60,71,74,76] though some societies in Ethiopia prefer to use root [46, 47, 69, 72, 89, 114] and Karamoja pastoralist uses bark for the preparation of remedies [36, 57, 58, 69, 78]. The uses of the leaf would better protect the plants than roots unless the people considers in using lateral roots than the taproots, which enable the plants to draw water from depth. On the other hand, excessive defoliation could also endanger the regeneration and survival of the plants in semi-arid and arid areas [17, 60]. Fresh plant parts and leaf are predominantly used in traditional treatments in Ethiopia. The use of fresh parts lessens the depletion of ingredients that affects the healing potential of the remedies through drying and storage. The people, when using fresh plants, they assume that ingredients in the plant are not lost through drying and curing potential is more than dried once [35, 41, 46, 76, 77, 115].

The practice of single plant for remedy preparation is common than a combination treatment in many studies conducted in Ethiopia and elsewhere [9, 30, 58] Nevertheless, in some regions such as in Gindeberet district, Western Ethiopia, 94% of the preparations are made from a mixture of multiple plant species [41, 49, 54, 58, 71, 82, 113]. The number of plants in multiple plant treatment ranged from two to ten and administered to disease where single plant preparations effectiveness is low. The people in the area perceived that use of multiple plants in preparation of ethnomedicine adds up the curing potential and confer synergetic effects [58]. On the other hand, the use of multiple plants to treat a disease is an indication of the prevalence and severity of illness in the region [41, 56, 58, 113]. The most popular plants used in multiple plant preparations in Yalo Woreda and other societies are *A. fruticosa* [53, 104], *A. tortilis* [20, 54, 64, 81, 115], *B. aegyptiaca* [19–21, 53, 62, 71, 104], *B. rotundifolia* [104, 116], *Indigofera oblongifolia* Forsk. [53, 104], *C. farinosa* [13, 53, 116], *Celosia polystachia* (Forssk.) C.C. Towns.), *Becium filamentosum* (Forssk.) Chiov. [41, 43] and *C. rotundifolia* [19, 53, 116].

The primary methods of preparation of remedies, crushing and pounding and diluents such as water and milk are similarly reported by other studies in Ethiopia [58, 60, 69]. The oral is the major route of administration of treatments in Yalo Woreda and a primary route of administration reported by many studies in Ethiopia and elsewhere [51–53, 56, 62, 77]. The application of remedies such as eye, nasal and ear drops, body washing, and insertion into a cut made in the knee, breast, or swollen body parts are dominantly used by other studies [9, 17, 43, 44, 54, 58, 69, 78, 82, 117]. The knowledge about health conditions of both humans and animals as in many studies in Ethiopia and Africa determines the types of treatment and the dosage. The measurement of a dose is related to the effectiveness of the remedy and levels of poisoning that has been determined through long years of trial and error [48, 54, 71]. The knowledge of standard practices considered in the management of remedies in traditional human healthcare systems is important factors to determine dosage, route, and frequency of applications [69, 78]*.* The treatment of animals is related to the manner of feeding and physical movement and, in most case, treatment is discontinued as the animal feeding, and physical status is improved, or sign of illness disappears. The remedies are given until the animal fully recovers, or its physical conditions are improved [51, 54].

The informants in the study area had displayed high consensus values on the plants used as a remedy that indicated the popularity and therapeutic value of the plants in the society and the prevalence of the disease in the area [46, 47]. The category of diseases with high ICF values are acute febrile illness and malaria; internal, liver and gastrointestinal infection, and internal parasite. The Yalo Woreda is malarious, and the people used 37 plant species to treat the illness, and 57 plant species as a treatment for liver and gastrointestinal infections that are developed over long years of trial and error similar to other studies conducted in the country [36, 41, 72, 77, 88]. The ethnobotanical study conducted in pastoral area Samburu district, Kenya has similar result that malaria and GIT are treated with 15 to 20 medicinal plants [49]. The majority of the plants with fidelity value of 1.00 was reported by few informants (one to two) and was used only as a treatment for an ailment. *Aizoon canariensis* L. is used to treat devil disease and madness, On the other hand, medicinal plants that were used to treat a variety of ailments had lower FL values. The values, in such cases, may not indicate the disagreement among the informants [51, 55] rather it means that these plants are more favored by the local people in the treatment of a variety of diseases such as diphtheria, typhoid, herpes zoster, scorpion bite, devil illness, ETPB, lung infection, dysentery, breast cancer, and angina [36, 41, 51]. The medicinal plants reported by 15 and more informants and used as a remedy for multiple diseases were ranked based on their healing potential of a disease. *Balanites rotundifolia* and *A. fruticosa* are used as remedies for diseases such as breast cancer, dyspepsia, epilepsy, ETPB, eye sickness, herpes zoster, infant disease, jaundice, lung infection, and malaria [17, 40, 57, 64, 68, 69, 81]. These plants have lower FL values indicating the popularity of the plants as a treatment for a variety of diseases. Therefore, preference ranking does not necessarily indicate healing potential of plants but also their popularity and may be abundance in the study area since their fruits are edible. Other societies also report the medicinal plants as a remedy for multiple diseases such as throat infection, stomachache/diarrhea; sexual incompetence of male; body infection; skin wound, snake bite, madness, typhus, eye problem, anthrax; rabies [10, 13, 22, 37, 64, 76, 82].

### Wild edible plants of Yalo Woreda

The wild edible plant parts are all eaten fresh, and duration of gathering and consumption are dependent on the availability of edible parts and seasons [34, 41, 46, 78]. The seasons are determined by the extent of rain and drought in the region. Sugum is the period of little rainfall (March to April), and Karma (June to September) is the main rain season. Gilal (October to February) and Hagay (May to June) are the dry seasons. Hagay extends to Karma and Gilal to Sugum depending on the length of rain and drought period [10–12, 80]. Borena, Kara, and Kwego pastoralist and people in semiarid areas in Ethiopia also consume the edible plants reported such as *B. aegyptiaca*, *B. rotundifolia*, *Dobera glabra* (Forssk.) Poir., *Carissa spinarum* L., *C.sinensis*, *G. bicolor, G. villosa, T.amarindus indica* L., *X americana* and *Z. spina-christi* [12, 13, 71, 78, 80, 118]. The people in semi-arid and arid areas rely more on edible plants compared to people that inhabit humid regions and highlands. These plants serve as food security during the dry season in semi-arid and arid areas where the recurrent drought have a dominant effect on animal’s productivity and could be developed to food crops to alleviate food shortage [78, 81, 119, 120]. *Balanites aegyptiaca* and *B. rotundifolia* and *D. glabra* are ‘famine food’ that are collected and stored for years in dry condition to be used in drought period to avoid starvation [12, 78, 80, 121]. These wild edible plants have been consumed by pastoralist people living in semi-arid and arid regions to alleviate food insecurity such as in the 1971 and 72 starvation in Ethiopia that severely affected Afar’s livestock. Similarly, Feyssa et al. [12] indicated that people in semi-arid and arid regions of Oromia state in Ethiopia, survived by eating fruits of *D. glabra* at the time of severe hunger. The wild edible fruits identified in Yalo Woreda with wider geographical distribution and altitude ranges are consumed by many societies in Ethiopia as reserve food to fill gaps between farming and harvest times where most poor farmers exhaust their crops. Unlike other parts of Ethiopia leaves, roots, and stems are not used as food in the Yalo Woreda [10–14, 20, 34, 46, 60, 62, 68, 78–81, 116, 119, 120, 122, 123]. *Balanites aegyptiaca, B. rotundifolia, C.sinensis*, and *D. glabra,* are sold in open markets and had economic importance [41]. Adults in eastern Sudan consume *Balanites aegyptiaca, Z. spina-christi, and T. indica* and have economic significance, and also have high marketability in other parts of Ethiopia [8, 34, 46, 62, 79, 123, 124].

## Conclusion

The people in Yalo Woreda possess a wealth of traditional knowledge on the treatment of both human and livestock health conditions, and edible plants. Fabaceae are the dominant plant species used in Ethnomedicine preparations. The people use a variety of measurements to quantify dosage and route of application in the administration of remedies. The edible plants are used as food security and generate the pastoralist economic. All the plants with medicinal and economic importance are collected from the wide and conservation is not practiced in the area. Hence, conservation of the plants in the home garden and in the natural vegetation is a necessity against the recurrent drought and climatic changes that negatively affect the vegetation of the area, to protect the associated traditional knowledge from fast disappearing and ensure sustainable use of the plants in the traditional healthcare system. The integration of edible plants in the food sufficiency strategies in the area has to be considered since animal productivity is severely affected by encroaching invasive plants and recurrent drought. The holistic soil and water conservation policy that is being implemented in other parts of the country has to be employed in the region to save the natural vegetation that is also the repository for the medicinal and edible plants for future pharmacological and nutritional studies.

## Additional files

Additional file 1: Medicinal plants used for treatment of human illness, Yalow Woreda, 2016 (B = bark, C = climber, F = flower, Fr = fruit, L = leaf, La = latex, R = root, S = stem, Br = branch, UP = upper part WP = whole plant, YP = young plant). (DOCX 179 kb)

Additional file 2: Medicinal plants used to treat animal illness, Yalow Woreda, 2016. (B = bark, C = climber, F = flower, Fr = fruit, L = leaf, La = latex, R = root, S = stem, Br = branch, UP = upper part WP = whole plant, YP = young plant). (DOCX 66 kb)

## **Abbreviations**

CCPP: Contagious caprine pleuropneumonia
Orf: Contagious pustular dermatitis, contagious ecthyma
PPR: Peste des petits ruminants
SPSS: Statistical Package for the Social Sciences
