# Supplementary material for: An ethnobotanical survey of medicinal and edible plants of Yalo Woreda in Afar regional state, Ethiopia
Source: J Ethnobiol Ethnomed. 2017 Jul 5;13:40. doi: 10.1186/s13002-017-0166-7 (PMC5499056; doi:10.1186/s13002-017-0166-7)
Supplement: Supplementary file 1 — Medicinal plants used for treatment of human illness, Yalow Woreda, 2016 (B = bark, C = climber, F = flower, Fr = fruit, L = leaf, La = latex, R = root, S = stem, Br = branch, UP = upper part WP = whole plant, YP = young plant). (DOCX 179 kb) [file 13002_2017_166_MOESM1_ESM.docx]

**Additional File 1 Medicinal plants used for treatment of human illness, Yalow Woreda, 2016 (B = bark, C= climber, F = flower, Fr = fruit, L = leaf, La = latex, R = root, S = stem, Br = branch, UP = upper part WP = whole plant, YP = young plant)**

| **Voucher Number** | **Local name of Disease** | **Disease** | **Scientific Name** | **Family** | **Habit** | **Local Name of Plant** | **Parts** | **Condition** | **Method of Preparation and application** |
| --- | --- | --- | --- | --- | --- | --- | --- | --- | --- |
| YA 072 | Ginyat | Knee sickness, swelling, | *Cordia sinensis* Lam. | Boraginaceae | Tree | Madera | Whole Plant | Fresh | All mixed, charred and ground and mixed with butter, salt and paper and is applied in cut made on knee, remains tied for five days |
| YA 045 |  |  | *Acacia oerfota* (Forssk.) Schweinf. | Fabaceae | Tree | Garomto | Stem | Fresh |  |
| YA 003 |  |  | *Cadaba rotundifolia* Forssk | Capparidaceae | Shrub | Adengelli | Stem | Fresh |  |
| YA 033 | Asso | Malaria | *Becium filamentosum* (Forssk.) Chiov. | Lamiaceae | Herb | Dob Dob | Leaf | Fresh | Crushed, mixed in water, filtrate is taken orally in morning for two days. |
| YA 073 | Gosom | Flue, headache | *Kanahia laniflora* (Forssk.) R.Br. | Asclepiadaceae | Shrub | Mali Karori | Root | Dry | Powdered and sniffed through nostrils in morning and at bed time for two days; not for children less than five |
| YA 007 | Baro Ali | Snake Bite | *Cyphostemma burgeri* Vollesen | Vitaceae | Climber | Ale Ili | Root | Fresh | Crushed and soaked in water and the filtrate is taken orally for one week. only Camel milk is taken |
| YA 098 | Alit | Infant sickness, thinness, fever, appetite lose | *Aloe trichosantha* Berger | Aloaceae | Shrub | Urina | Leaf & Stem | Fresh | Crushed and soaked in water; filtrate is taken orally for three days and residue is used to wash body of infant |
| YA 069 | Dulaya | Retained placenta | *Ziziphus spina-christi* (L.) Desf. | Rhamnaceae | Tree | Kusra | leaf | Fresh | Crushed and soaked in water; filtrate is taken orally three times; morning, mid-day and evening until expelled |
| YA 019 | Asso | Malaria, chill | *Acalypha indica* L. | Euphorbiaceae | Herb | Baroberberie | Leaf | Fresh | Crushed and mixed with water, 1 glass filtrate is taken orally before breakfast for two days |
| YA 033 | Geno | Coughing blood through mouth and nostrils | *Becium filamentosum* (Forssk.) Chiov. | Lamiaceae | Herb | Dob Dob | Leaf & Stem | Fresh | Mixed crushed leaf is soaked in water; 1 glass filtrate is taken orally two times per day; morning and evening. If not cured it is will be replaced with Dunelita |
| YA 034 |  |  | *Cadaba glandulosa* Forssk. | Capparidaceae | Shrub | Dunelita | Leaf & Stem | Fresh |  |
| YA 039 | Gero | Bloody dysentery, Amoeba | *Acacia seyal* Del. | Fabaceae | Tree | Galiela | Root | Fresh | Powdered and soaked in water and two tea cup filtrate is taken orally for one day |
| YA 015 | Andero | Thing, fever, green blood veins | *Indigofera articulata* Gouan | Fabaceae | Shrub | Ayrowegit | Root | Fresh | Crushed root is soaked in water overnight, 1 tea cup filtrate is taken orally in the morning for three days |
| YA 005 | Ulahama | Green - yellow eye, fever. Side pain | *Cymbopogon commutatus* (Steud.) Stapf | Poaceae | Herb | Afeka | Tuber | Dry/Fresh | Ground on stone, mixed with sheep’s rumen paste and used to wash body for one day |
| YA 077 | Kida | Breast swelling, no milk production | *Aerva javanica* (Burm.f) Schultes | Amaranthaceae | Herb | Olayto | Leaf | Fresh | Crushed, soaked in water for a night; the filtrate is applied through nostrils and the residue used to wash breast for a day |
| YA 066 |  |  | *Commicarpus helenae* (J.A. Schultes) Meikle | Nyctaginaceae | Herb | Kerebtu | Leaf | Fresh |  |
| YA 025 |  |  | *Citrullus lanatus* (Thunb.) Matsum. & Nakai | Cucurbitaceae | Climber | Dare Ebto | Leaf | Fresh |  |
| YA 087 | Undufeyta | Body infection, thinning, appetite loss | *Aristolochia bracteolata* Lam. | Aristolochiaceae | Herb | Suie Suie | Whole Plant | Dry | Crushed and dried in sun, charred, powdered, mixed with butter and applied on the body topically for three days |
| YA 008 | Asso | Malaria | *Balanites rotundifolia* (Van Tiegn.) Blatter | Balanitaceae | Shrub | Alayto | Leaf | Fresh | Crushed, soaked in water for overnight with some salt a cup of filtrate is taken orally before meal for thee day |
| YA 078 | Antiredo | Eye sickness | *Cadaba farinosa* Forssk. | Capparidaceae | Shrub | Ormayto | Leaf | Fresh | Crushed with water, boiled by inserting red-hot iron and the fume is inhaled covered by overcoat |
| YA 003 |  |  | *Cadaba rotundifolia* Forssk | Capparidaceae | Shrub | Adengeli | Leaf | Fresh |  |
| YA 086 |  |  | *Acalypha fruticosa* Forssk. | Euphorbiaceae | Shrub | Subahsila | Leaf | Fresh |  |
| YA 102 | Baro Ali | Snake Bite | *Olea europaea L. subsp. cuspidata* (Wall.ex G. Don) Cif. | Oleaceae | Tree | Weger | Stem | Fresh | Chewing and eating for one time |
| YA 050 | Hindo | Impotence | Seddera bagshawei Rendle | Convolvulaceae | Shrub | Bekilbetergei | Root | Dry | Crushed, dry in sun, powdered, mixed with butter and given through nostrils for three days |
| YA 088 | Habu | Madness | *Dorstenia barnimiana* Schweinf. | Moraceae | Herb | Suwawur | Whole Plant | Dry | Dried under shade, and put on red-hot charcoal and inhale the smoke coved for seven days |
| YA 008 | Asso | Malaria | *Balanites rotundifolia* (Van Tiegn.) Blatter | Balanitaceae | Shrub | Alayto | Leaf | Fresh | Chewing and swallowing juice before meal for seven days |
| YA 034 | Utuka/ Gano | headache, burning of hand and foot | *Cadaba glandulosa* Forssk. | Capparidaceae | Shrub | Dunelita | leaf | Fresh | Pounded and soaked in water and filtrate is taken orally and used for body washing |
| YA 039 | Alsam | Stomachache, bloating, dysentery | *Acacia seyal* Del. | Fabaceae | Tree | Gal Ela | Root | Fresh | Crushed mixed with water and taken orally or chewing for two days |
| YA 095 | Dedeweyta | sleepless with fever, restless | *Balanites aegyptiaca* (van Tieghem) Blatter | Balanitaceae | Tree | Udayto | Root | Fresh | Crushed in water, vigorously stirred until foam appeared; 1 glass filtrate is orally taken in morning and evening for one day |
| YA 040 | Genobiak | fever, bloody dysentery | *Withania somnifera* (L.) Duna | Solanaceae | Shrub | Gale ela | Root | Fresh | Crushed, soaked in water and 1 glass of filtrate is taken orally |
| YA 001 | Alit | Infant dysentery | *Ocimum urticifolium* Roth | Lamiaceae | Shrub | Abushefi | Leaf | Fresh | Pounded, soaked in water and 1 cup filtrate is given orally and nostril; for 2 or 3 days; the residue is for washing |
| YA 095 | Kida | Neck and breast cancer | *Balanites aegyptiaca* (van Tieghem) Blatter | Balanitaceae | Tree | Udayto | Leaf | Fresh | Pounded, soaked in water and one glass filtrate is given orally and nostril; the residue is for bathing for 2 or 3 days; |
| YA 001 | Alibiya | Infant disease; coughing, respiratory | *Ocimum urticifolium* Roth | Lamiaceae | Shrub | Abushefi | Leaf | Fresh | Pounded, soak in water for hours; 1 cup filtrate is given orally and nostril three times for one day and for body wash |
| YA 097 | Halib | Diabetes | *Solanum marginatum* Lf | Solanaceae | Shrub | Ungule | Leaf | Fresh | Crushed with water and boiled overnight and one glass filtrate is given orally three times for one day |
| YA 034 | Geno | stomachache and bloating, | *Cadaba glandulosa* Forssk. | Capparidaceae | Shrub | Dunelita | Leaf | Fresh | Crushed mixed with water and filtrate is taken orally three times per day for one days |
| YA 042 | Bekaie | Head Wound | *Dobera glabra* (Forssk.) Poir. | Salvadoraceae | Shrub | Gasera | Bark | Dry | Pounded, and powder is put on the wound for two days |
| YA 087 | Undufeyta | Infection on skin | *Aristolochia bracteolata* Lam. | Aristolochiaceae | Herb | Sue suie | Leaf | Fresh | Pounded and mixed with water and filtrate is taken orally early morning for three days and the residue is put on the wound |
| YA 042 | Bekaie | Fresh Head wound | *Dobera glabra* (Forssk.) Poir. | Salvadoraceae | Shrub | Gasera | Leaf | Dry | Dry under sun, ground and powder is topically applied on wound for two days |
| YA 075 | Gosom | Flue | *Acacia mellifera* (Vahl) Benth. | Fabaceae | Tree | Lakasi | Leaf | Fresh | Rapped with closed and sniffed until cured |
| YA 045 | Jiniate | Swelling of Knee, stiffness | *Acacia oerfota* (Forssk.) Schweinf. | Fabaceae | Tree | Ajo | Stem | Fresh | Mixed, charred and powdered, mixed with the fruits and ground and paste is made with blood of black sheep. Paste is applied in a cut made on swollen knee with *C. annuum* L. leaf for three days |
| YA 060 |  |  | *Capsicium annuum* L. | Solanaceae | Shrub | Hindi | Stem | Fresh |  |
| YA 003 |  |  | *Cadaba rotundifolia* Forssk | Capparidaceae | Shrub | Adengeli | Stem | Fresh |  |
| YA 072 |  |  | *Cordia sinensis* Lam. | Boraginaceae | Tree | Madera | Fruit | Fresh |  |
| YA 088 | Habule | Schizophrenia | *Dorstenia barnimiana* Schweinf. | Moraceae | Herb | Suwawur | Leaf | Fresh/dry | Crushed and put on hot red charcoal and inhales the smoke covered by blanket treatment continues until cured |
| YA 086 | Gano | Throat infection | *Acalypha fruticosa* Forssk. | Euphorbiaceae | Shrub | Subahsila | Leaf | Fresh | Crushed and soaked in water for few hours and the filtrate is given orally and nostril |
| YA 003 |  |  | *Cadaba rotundifolia* Forssk | Capparidaceae | Shrub | Adengeli | Leaf | Fresh |  |
| YA 095 | Aliberik | Flue | *Balanites aegyptiaca* (van Tieghem) Blatter | Balanitaceae | Tree | Udayto | Leaf | Fresh | Crushed and boiled in water and inhaling the fume covered by overcoat |
| YA 086 |  |  | *Acalypha fruticosa* Forssk. | Euphorbiaceae | Shrub | Subahsila | Leaf | Fresh |  |
| YA 003 |  |  | *Cadaba rotundifolia* Forssk | Capparidaceae | Shrub | Adengeli | Leaf | Fresh |  |
| YA 086 | Gubllo | Lung Infection | *Acalypha fruticosa* Forssk. | Euphorbiaceae | Shrub | Subahsila | Leaf | Fresh | Pounded with water and filtrate with butter is taken orally; boiled and steam is inhaled |
| YA 078 | Gubllo | Lung Infection | *Cadaba farinosa* Forssk. | Capparidaceae | Shrub | Ormayto | Leaf | Fresh | Crushed and covered with close and sniffing through nostrils |
| YA 073 | Gelhum Manga | Asthma | *Kanahia laniflora* (Forssk.) R.Br. | Asclepiadaceae | Shrub | Delhi Manga | Leaf | Fresh | Crushed, boiled and inhaling the fume coved with close |
| YA 086 | Gubllo | Lung Infection | *Acalypha fruticosa* Forssk. | Euphorbiaceae | Shrub | Subahsila | Leaf | Fresh | Crushed, boiled and inhaling the fume coved with close |
| YA 008 | Gosom | Coughing | *Balanites rotundifolia* (Van Tiegn.) Blatter | Balanitaceae | Shrub | Alayto | Leaf | Fresh | Chewed and swallowing the juice before meal |
| YA 078 | Utuka/ Gano | Mitch | *Cadaba farinosa* Forssk. | Capparidaceae | Shrub | Ormayto | Leaf | Fresh | Mixed, Crushed and boiled in water and inhaling the fume; also Crushed covered with close and sniffing through nostrils |
| YA 003 |  |  | *Cadaba rotundifolia* Forssk | Capparidaceae | Shrub | Adengeli | Leaf | Fresh |  |
| YA 086 |  |  | *Acalypha fruticosa* Forssk. | Euphorbiaceae | Shrub | Subahsila | Leaf | Fresh |  |
| YA 095 |  |  | *Balanites aegyptiaca* (van Tieghem) Blatter | Balanitaceae | Tree | Adayto | Leaf | Fresh |  |
| YA 095 | Gano | Blood Coughing , chest pain | *Balanites aegyptiaca* (van Tieghem) Blatter | Balanitaceae | Tree | Udayto/ Bedeno | Root & Bark | Fresh | Pounded and boiled in water and filtrate is taken orally |
| YA 019 | Asthma | Asthma | *Acalypha indica* L. | Euphorbiaceae | Herb | Baroberberie | Leaf | Fresh | Pounded with water and filtrate is taken orally |
| YA 095 | Gubllo | Coughing blood through mouth and nostrils | *Balanites aegyptiaca* (van Tieghem) Blatter | Balanitaceae | Tree | Uda (Molao) | Bark | Dry | Pounded and soaked in water and 1 and 1/2 cup filtrate is taken orally |
| YA 019 | Mudunta | Chest Pain | *Acalypha indica* L. | Euphorbiaceae | Herb | Baroberberie | Leaf | Fresh | Pounded with water and filtrate is taken orally |
| YA 033 | Alsat | Vomiting | *Becium filamentosum* (Forssk.) Chiov. | Lamiaceae | Herb | Dob Dob | Leaf | Fresh | Mixed Crushed and soaked in water and filtrate is given two times |
| YA 066 | Alsat | Vomiting | *Commicarpus helenae* (J.A. Schultes) Meikle | Nyctaginaceae | Herb | Sati | Leaf | Fresh | Mixed Crushed and soaked in water and filtrate is given two times |
| YA 043 | Geno | Sun sudden illness, fever, dark blood vessel, | *Calotropis procera* (Ait.) Ait.f. | Apocynaceae | Shrub | Gela ato | Flower-bud | Dry | Dried and powdered; mixed with water and filtrate is applied through nostrils three times for a day. |
| YA 001 | Begero | Stomachache | *Ocimum urticifolium* Roth | Lamiaceae | Shrub | Safie | Leaf | Fresh | Pounded and mixed with water and 1 glass of filtrate is applied orally. |
| YA 075 | Gara Harerie | Fire-burn | *Acacia mellifera* (Vahl) Benth. | Fabaceae | Tree | Merka ato | Stem | Fresh | Saliva is spitted on burned part before meal and stem charred, powdered mixed with skimmed goat milk is applied topically on burned part |
| YA 076 | Unduf Feyta | Infection on skin and mouth | *Plicosepalus robustus* Wiens & Polhil | Loranthaceae | Shrub | Merka ato/Atokele | Whole Plant | Fresh | Crushed, mixed with water and filtrate is taken orally and applied through nostril for 3 days. Residue is for body washing |
| YA 062 | Amoska | head wound | *Grewia erythraea* Schweinf. | Tiliaceae | Shrub | Hudayto | Stem-bark | Fresh | Mixed, charred, powdered and mixed with skimmed milk and applied topically on the wound until cure |
| YA 075 |  |  | *Acacia mellifera* (Vahl) Benth. | Fabaceae | Tree | Merka ato | stem | Fresh |  |
| YA 062 | Geno | Stomachache | *Grewia erythraea* Schweinf. | Tiliaceae | Shrub | Hudayto | Stem bark | Fresh | Pounded mixed with water the filtrate is taken orally two times per day until cured |
| YA 045 | Iyala | Children Mitch, malaria | *Acacia oerfota* (Forssk.) Schweinf. | Fabaceae | Tree | Garomto | Leaf/root | Fresh | Mixed Crushed and soaked in water and filtrate is given orally and through nostrils for three days. The residue is used to wash the body |
| YA 001 |  |  | *Ocimum urticifolium* Roth | Lamiaceae | Shrub | Abushafi | Leaf | Fresh |  |
| YA 055 |  |  | *Seddera hirsute* Dammer ex Hall. f. | Convolvulaceae | Shrub | Harmela | Leaf | Fresh |  |
| YA 035 | Kena (sinaredwek) | Thorn injury | *Acacia tortilis* (Forssk.) Schweinf. | Fabaceae | Tree | Eabeto | Leaf | Fresh | Pounded and applied on the wound until cure |
| YA 082 | Solot | Swelling after circumcise | *Senna alexandrina* Mill. | Fabaceae | Shrub | Seno | Leaf latex | Fresh | latex of Crushed leaf is applied on circumcised area |
| YA 082 | Begofoy | Stomach bloating | *Senna alexandrina* Mill. | Fabaceae | Shrub | Seno | Leaf | Fresh | Boiled three times overnight in water and leaf is eaten before breakfast |
| YA 008 | Asso | Malaria | *Balanites rotundifolia* (Van Tiegn.) Blatter | Balanitaceae | Shrub | Alayto | Leaf | Fresh | Chewing and swallowing the juice before breakfast for seven days |
| YA 008 | Asso | Malaria | *Balanites rotundifolia* (Van Tiegn.) Blatter | Balanitaceae | Shrub | Alayto | Leaf | Fresh | Pounded and boiled in water at the evening and 1 glass filtrate is taken before breakfast for three days |
| YA 098 | Intidayla | Eye sickness | *Aloe trichosantha* Berger | Aloaceae | Shrub | Unrena | Latex | Fresh | Drops of latex exudates under the thorn is applied on eye in the morning and the evening for two days |
| YA 079 | Urara | Dysentery | *Tapinanthus globiferus* (A. Rich.) Tieghem | Loranthaceae | Shrub | Ormayto-Atokele | Leaf | Fresh | Crushed leaf is boiled in water or milk for 2 hours at night and filtrate is taken two times per day in morning and evening before meal. |
| YA 079 | Kidu | Impotence | *Tapinanthus globiferus* (A. Rich.) Tieghem | Loranthaceae | Shrub | Ormayto-Atokele | Leaf | Fresh | Crushed leaf is boiled in water or milk for 2 hours at night and filtrate is taken two times per day in morning and evening before meal. |
| YA 079 | Halika | Indigestion | *Tapinanthus globiferus* (A. Rich.) Tieghem | Loranthaceae | Shrub | Ormayto-Atokele | Leaf | Fresh | Crushed leaf is boiled in water or milk for 2 hours at night and filtrate is taken two times per day in morning and evening before meal. |
| YA 029 | Indaroyta | Green-yellow eye color | Oncocalyx schimperi (A. Rich.) M. Gilbert | Loranthaceae | Shrub | Denunelita (Atokele) | Leaf | Fresh | Crushed leaf is boiled in water or milk for 2 hours at night and filtrate is taken two times per day in morning and evening before meal. |
| YA 029 | Anderoyta | Intestinal parasites, Ascaris, Amoeba | Oncocalyx schimperi (A. Rich.) M. Gilbert | Loranthaceae | Shrub | Denunelita (Atokele) | Leaf | Fresh | Crushed leaf is boiled in water or milk for 2 hours at night and filtrate is taken two times per day in morning and evening before meal. |
| YA 055 | Baro Ali | Snake bite | *Seddera hirsute* Dammer ex Hall. f. | Convolvulaceae | Shrub | Harmela | Stem | Fresh | Chewing and juice is swallowed, saliva after reading Kuran continued for three/seven days |
| YA 035 | Urbiya | Infant sickness, fever, | *Acacia tortilis* (Forssk.) Schweinf. | Fabaceae | Tree | Ebeto (kura) 1 | Young leaf | Fresh | Young leaf Pounded with water and applied topically on the whole body; S. alexandrina leaf is Pounded in water and the filtrate is given through nostrils; F. paulayana upper part,' leaf, stem, thorn is Pounded and filtrate is given orally and through nostrils three times per day until cured |
| YA 082 |  |  | *Senna alexandrina* Mill. | Fabaceae | Shrub | Seno 2 | leaf | Fresh |  |
| YA 031 |  |  | *Fagonia paulayana* Wagner & Vierh. | Zygophyllaceae | Shrub | Dibolie 3 | Upper part' leaf, stem, thorn | Fresh |  |
| YA 101 | Iyala | Infant sickness, weak, dizzy and unconscious | *Orthosiphon pallidus* Royle ex Benth. | Lamiaceae | Herb | Weder kehena | leaf | Fresh | Mixed and Pounded together soaked in water and filtrate is taken orally, through nostrils, ear and topically applies on hand and feet and remaining used for body washing for seven days |
| YA 008 |  |  | *Balanites rotundifolia* (Van Tiegn.) Blatter | Balanitaceae | Shrub | Alayto | upper part | Fresh |  |
| YA 013 |  |  | *Indigofera oblongifolia* Forssk. | Fabaceae | Shrub | Ayrowegit (Male) | upper part | Fresh |  |
| YA 086 |  |  | *Acalypha fruticosa* Forssk. | Euphorbiaceae | Shrub | Subahsila | leaf | Fresh |  |
| YA 045 |  |  | *Acacia oerfota* (Forssk.) Schweinf. | Fabaceae | Tree | Garomto | leaf | Fresh |  |
| YA 045 | Dahowaso | Unable to urinate, venereal | *Acacia oerfota* (Forssk.) Schweinf. | Fabaceae | Tree | Garomto | root bark | Fresh | Pounded and dissolved in water and filtrate is taken orally for two days. the soil on the root is applied topically on the genital organ of men |
| YA 013 | Genobiak | Bloody dysentery, fever | *Indigofera oblongifolia* Forssk. | Fabaceae | Shrub | Ayrowegit (Male) | leaf and stem | Fresh | Pounded together mixed in water and filtrate is taken orally and no water for two days |
| YA 008 | Asso | Malaria | *Balanites rotundifolia* (Van Tiegn.) Blatter | Balanitaceae | Shrub | Alayto | Leaf | Fresh | Pounded and macerated in water overnight and filtrate taken orally before breakfast |
| YA 103 | Kida | Breast swelling and infection | *Celosia polystachia* (Forssk.) C.C. Townsend | Amaranthaceae | Herb | Werabikela | leaf | Fresh | Mixed and Pounded together soaked in water and filtrate is taken orally, through nostrils, and topically applied on breast for three days and remaining used for body washing |
| YA 086 |  |  | *Acalypha fruticosa* Forssk. | Euphorbiaceae | Shrub | Subahsila | leaf | Fresh |  |
| YA 034 |  |  | *Cadaba glandulosa* Forssk. | Capparidaceae | Shrub | Dunelita | leaf | Fresh |  |
| YA 045 |  |  | *Acacia oerfota* (Forssk.) Schweinf. | Fabaceae | Tree | Garomto | leaf | Fresh |  |
| YA 035 |  |  | *Acacia tortilis* (Forssk.) Schweinf. | Fabaceae | Tree | Eabeto | leaf | Fresh |  |
| YA 083 |  |  | *Senna italica* Mill. | Fabaceae | Herb | Senu | leaf | Fresh |  |
| YA 001 | Iyala | Amora sickness, fever, dysentery, and vomiting | *Ocimum urticifolium* Roth | Lamiaceae | Shrub | Abushafi | Whole Plant | Fresh | Pounded and macerated and filtrate is taken orally for four days and on forth day remaining mixture is used for body washing |
| YA 086 | Tumewea | Swelling | *Acalypha fruticosa* Forssk. | Euphorbiaceae | Shrub | Subahsila | Leaf | Fresh | Pounded and mixed with water and filtrate is taken orally three time per day before meal and on fourth day used for body washing; every day new preparation is made for application |
| YA 021 |  |  | *Ruellia patula* Jacq. | Acanthaceae | Shrub | Boboyta | Leaf | Fresh |  |
| YA 050 |  |  | *Seddera bagshawei* Rendle | Convolvulaceae | Shrub | Halemagayra | Whole Plant | Fresh |  |
| YA 028 |  |  | *Cucumis prophetarum* L. | Cucurbitaceae | Climber | Denkakebisei | Whole Plant | Fresh |  |
| YA 041 | Gemedi delayi | Retained placenta | *Acacia nilotica* (L.) Willd. Ex Del. | Fabaceae | Tree | Gansel | Leaf | Fresh | Mixed Pounded and macerated in water and filtrate is taken orally until expelled |
| YA 023 |  |  | *Premna oligotricha* Baker | Lamiaceae | Shrub | Bota | Leaf | Fresh |  |
| YA 001 | Geno | Stomach-ach, fever, thirsty | *Ocimum urticifolium* Roth | Lamiaceae | Shrub | Abushafi | upper part | Fresh | Pounded and macerated in water for one hour and filtrate is taken orally before breakfast |
| YA 098 | Alidehenum | Snake bite | *Aloe trichosantha* Berger | Aloaceae | Shrub | Hurayto | Root | Fresh | Mixed and Pounded and macerated in water taken orally and through nostrils for three days and mixture is used to wash body. Every application is new preparation |
| YA 040 |  |  | *Withania somnifera* (L.) Dunal | Solanaceae | Shrub | Burahinhara | Leaf | Fresh |  |
| YA 086 | Lahidelea | Kola kusil, Wound, infection | *Acalypha fruticosa* Forssk. | Euphorbiaceae | Shrub | Subahsila | Leaf | Fresh | Mixed, Pounded, and mixed with water and whole filtrate is taken orally the whole day in place of water |
| YA 095 |  |  | *Balanites aegyptiaca* (van Tieghem) Blatter | Balanitaceae | Tree | Udayto | Leaf | Fresh |  |
| YA 055 | Liena | Fever | *Seddera hirsute* Dammer ex Hall. f. | Convolvulaceae | Shrub | Harmela | Whole Plant | Fresh | Pounded mixed with water and filtrate is taken orally whole day for one day |
| YA 033 | Ametaki | Sleep waking | *Becium filamentosum* (Forssk.) Chiov. | Lamiaceae | Herb | Dodew | Leaf | Fresh | Mixed and Pounded and soaked in water for hours and filtrate is taken orally for seven days and washes body with residue and C. pendulus leaf |
| YA 053 |  |  | *Cocculus pendulus* (J. R. & G. Forst) Diels | Menispermaceae | Climber | Haratawea hara | Leaf | Fresh |  |
| YA 020 |  |  | *Kleinia squarrosa* Cufod. | Asteraceae | shrub | Utewri | Leaf | Fresh |  |
| YA 004 |  |  | *Allium sativum* L. | Alliaceae | Herb | Bisel | Fruit | Fresh |  |
| YA 096 |  |  | *Nigella sativa* L. | Ranunculaceae | Herb | Udo Besel | Fruit | Fresh |  |
| YA 094 |  |  | *Ferula communis* L. | Apiaceae | Herb | Ubewused | Fruit | Fresh |  |
| YA 093 |  |  | *Parthenium hysterophorus* L. | Asteraceae | Herb | Ubder Kemun | Fruit | Fresh |  |
| YA 022 |  |  | *Justicia schimperiana* (Hochst. ex Nees) T. Anders. | Acanthaceae | Shrub | Boroseley | Fruit | Fresh |  |
| YA 084 |  |  | *Lepidium sativum* L. | Brassicaceae | Herb | Shinfaye | Fruit | Fresh |  |
| YA 071 |  |  | *Citrus lemon* (L.) Bunn.f. | Rutaceae | Tree | Lomin | Fruit | Fresh |  |
| YA 043 | Begu biyak / Gano | Stomachache, bloody dysentery | *Calotropis procera* (Ait.) Ait.f. | Apocynaceae | Shrub | Gela ato | Fruit | Fresh | Crushed, soaked in water and filtrate is taken morning and evening until cure |
| YA 022 | Urubiak | Infant sickness weak, dizzy and unconscious | *Justicia schimperiana* (Hochst. ex Nees) T. Anders. | Acanthaceae | Shrub | Gaboyta | Bark | Fresh | Bark mixed with water Pounded one cup filtrate is given per day |
| YA 003 | Igiltu | Placenta | *Cadaba rotundifolia* Forssk | Capparidaceae | Shrub | Adengelli | Root | Fresh | Mixed and Pounded with water and one cup filtrate is given for a day |
| YA 068 |  |  | *Ziziphus mauritiana* Lam. | Rhamnaceae | Tree | Kusra | Leaf | Fresh |  |
| YA 026 | Habatenum | Madness | *Citrullus colocynthis* (L.) Schrad. | Cucurbitaceae | Climber | Dearteba | Leaf | Fresh | Mixed, Pounded and with water and 1 glass of filtrate is applied orally for three days. |
| YA 097 |  |  | *Solanum marginatum* Lf | Solanaceae | Shrub | Ungulay | Leaf & Root | Fresh |  |
| YA 079 | Undufeyta | Mouth, tooth Infection, swelling | *Tapinanthus globiferus* (A. Rich.) Tieghem | Loranthaceae | Shrub | Atokele | ` | Fresh | Gum is rubbed with Atokele leaves until blood appear and mixed, Pounded, with water and the filtrate is applied though ear and taken orally and used to wash body for three days |
| YA 013 |  |  | *Indigofera oblongifolia* Forssk. | Fabaceae | Shrub | Ayrowegit (Male) | Root | Fresh |  |
| YA 069 |  |  | *Ziziphus spina-christi* (L.) Desf. | Rhamnaceae | Tree | Tethara | Root | Fresh |  |
| YA 103 | Gano | Epilepsy | *Celosia polystachia* (Forssk.) C.C. Townsend | Amaranthaceae | Herb | Werabikela | Leaf | Fresh | Mixed and Pounded with water; one glass filtrate is given per day for two days |
| YA 015 |  |  | *Indigofera articulata* Gouan | Fabaceae | Shrub | Ayrowegit (Female) | Leaf & Root | Fresh |  |
| YA 086 |  |  | *Acalypha fruticosa* Forssk. | Euphorbiaceae | Shrub | Subahsila | Leaf | Fresh |  |
| YA 075 | Bekeaenum | Broken head skin | *Acacia mellifera* (Vahl) Benth. | Fabaceae | Tree | Merka ato | Branch | Fresh | Charred and powdered mixed with butter and applied topically on the wound |
| YA 016 | Igiltu | Retained Placenta | *Boscia coriacea* Pax. | Capparidaceae | Tree | Aytinaeba | Root | Fresh | It is tied around waist and placenta is expelled |
| YA 066 | Lukufto | Elephantiasis | *Commicarpus helenae* (J.A. Schultes) Meikle | Nyctaginaceae | Herb | Seati | Leaf | Fresh | Crushed and mixed with butter and applied topically on swollen foot |
| YA 016 | Lahidelea | Infection and Wound on body (leprosy) | *Boscia coriacea* Pax. | Capparidaceae | Tree | Ayitnaeba | Leaf | Fresh | Dry on fire, Pounded and mixed with honey applied topically on the infection in morning for three days |
| YA 081 | Diglo | Broken bone | *Parkinsonia scioana* (Chiov.) Brenan | Fabaceae | Shrub | Sekuhto Alo | Bark | Fresh | Mixed, Pounded and mixed with goat blood and tied around broken bone |
| YA 048 |  |  | *Grewia villosa* Willd. | Tiliaceae | Shrub | Habeleyta | Root | Fresh |  |
| YA 041 | Bekea | Broken head | *Acacia nilotica* (L.) Willd. Ex Del. | Fabaceae | Tree | Gansol | Brach | Fresh | Mixed, Pounded and mixed with butter and boiled, and when cool applied on the wound |
| YA 003 |  |  | Cadaba rotundifolia Forssk | Capparidaceae | Shrub | Unguleayta | Leaf | Fresh |  |
| YA 077 | Sunini | Blooding nose | *Aerva javanica* (Burm.f) Schultes | Amaranthaceae | Herb | Olayto | Root | Fresh | Crushed and liquid is added through nostril |
| YA 081 | Alsat | Stomachache, bloody dysentery | *Parkinsonia scioana* (Chiov.) Brenan | Fabaceae | Shrub | Sekuhto Alo | Bark | Fresh | Mixed with water, Pounded and one glass filtrate is taken orally for one day |
| YA 077 | Alsat | Vomiting | *Aerva javanica* (Burm.f) Schultes | Amaranthaceae | Herb | Olayto | Root | Fresh | Mixed with water, Pounded and one glass filtrate is taken orally for one day/ until cure |
| YA 040 | Butamara | Evil eye | *Withania somnifera* (L.) Dunal | Solanaceae | Shrub | HalaHara | Root | Fresh | Root is added on hot charcoal and patient inhale the smoke covered |
| YA 014 | Barogie | Hand swelling | *Trianthema portulacastrum* L. | Aizoaceae | Herb | Ayrolasa | Leaf | Fresh | Mixed Crushed and mixed with butter and topically applied on swollen hand |
| YA 007 |  |  | *Cyphostemma burgeri* Vollesen | Vitaceae | Climber | Urina | Leaf | Fresh |  |
| YA 008 | Asla | Stomach cleaning, | *Balanites rotundifolia* (Van Tiegn.) Blatter | Balanitaceae | Shrub | Alayto | Leaf | Fresh | Mixed with salt and Pounded and one glass filtrate is taken |
| YA 103 | Asla | Stomachache, bloody dysentery | *Celosia polystachia* (Forssk.) C.C. Townsend | Amaranthaceae | Herb | Werabikela | Leaf | Fresh | Pounded, mixed with water and filtrate is taken orally repeatedly for six hours |
| YA 066 | Lukufto | Elephantiasis | *Commicarpus helenae* (J.A. Schultes) Meikle | Nyctaginaceae | Herb | Seati | Leaf | Fresh | Pounded mixed with water and swollen foot is massaged with filtrate |
| YA 091 | Lahidelea | Spreading infection and wound on body | *Acacia senegal* (L.) Wild. | Fabaceae | Tree | Tiki Kelea | Leaf | Fresh | Pounded and mixed with water and applied on the wound and filtrate is added through nostril morning and evening for two days |
| YA 078 | Afasi | Swelling on body and infection | *Cadaba farinosa* Forssk. | Capparidaceae | Shrub | Galikihna | Leaf | Fresh | Pounded and applied topically on swollen part and filtrarilte is added through nostril |
| YA 052 | Butamara | Eye sickness | *Abutilon figarianum* Guill. & Perro | Malvaceae | Herb | Hamketo | Root | Fresh | Dried, Pounded and put on hot charcoal and fumigated by the smoke |
| YA 082 | Ali adehenum | Snake bite | *Senna alexandrina* Mill. | Fabaceae | Shrub | Seno | Leaf | Fresh | Pounded and spot is washed with filtrate and one glass is taken orally and 5 ml is added through the nostril morning and evening for three days |
| YA 095 | Tumea | Swelling on body | *Balanites aegyptiaca* (van Tieghem) Blatter | Balanitaceae | Tree | Udayto | Leaf | Fresh | Pounded and filtrate is given orally and through nostrils and applied on swollen part |
| YA 045 | Iyala | Baby sickness, dysentery, vomiting | *Acacia oerfota* (Forssk.) Schweinf. | Fabaceae | Tree | Garomto | Leaf | Fresh | Pounded with water and body is washed by filtrate and applied through nostril |
| YA 010 | Qurho | Tonsillitis | *Fagonia schweinfurthii* Hadidi | Zygophyllaceae | Shrub | Arengamo | Root | Fresh | Crushed mixed with water and filtrate is taken before breakfast for three days |
| YA 063 | Geno | Headache, blood through nose and mouth | *Tamarindus indica* L. | Fabaceae | Tree | Hura | Bark | Fresh | Mixed and Dried in sun, Pounded and the powder is mixed in water taken orally until cured or covered with closed and sniffed through nostrils |
| YA 095 |  |  | *Balanites aegyptiaca* (van Tieghem) Blatter | Balanitaceae | Tree | Udayto/ Bedeno | Bark and Root | Fresh |  |
| YA 015 | Geratokmenum | Fire burn | *Indigofera articulata* Gouan | Fabaceae | Shrub | Ayrowegit | Leaf | Fresh | Charred and ground applied on affected part |
| YA 013 | Baro | Scorpion bite | *Indigofera oblongifolia* Forssk. | Fabaceae | Shrub | Ayrowegit (Male) | Root | Fresh | Chewing and swallowing the juice and tying on the wound |
| YA 013 | Juni Utuka | Devil illness/ Mitch | *Indigofera oblongifolia* Forssk. | Fabaceae | Shrub | Ayrowegit (Male) | Leaf | Fresh | Mixed and Crushed with water and used for body washing until cure |
| YA 083 |  |  | *Senna italica* Mill. | Fabaceae | Herb | Senid | Leaf | Fresh |  |
| YA 082 |  |  | *Senna alexandrina* Mill. | Fabaceae | Shrub | Aliseno | Leaf | Fresh |  |
| YA 013 |  |  | *Indigofera oblongifolia* Forssk. | Fabaceae | Shrub | Ayrowegit (Male) | whole plant | Fresh |  |
| YA 094 |  |  | *Ferula communis* L. | Apiaceae | Herb | Harashif (Underkamun) | Leaf | Fresh |  |
| YA 043 | Agu dudubtie | Breast swelling | *Calotropis procera* (Ait.) Ait.f. | Apocynaceae | Shrub | Geleato | Leaf | Fresh | Mixture is Crushed and topically applied on the breast and sniffed through nostrils |
| YA 086 |  |  | *Acalypha fruticosa* Forssk. | Euphorbiaceae | Shrub | Subahsila | Leaf | Fresh |  |
| YA 066 |  |  | *Commicarpus helenae* (J.A. Schultes) Meikle | Nyctaginaceae | Herb | Karbato | Leaf | Fresh |  |
| YA 001 | Alsimya | Stomachache Bloating, restlessness | *Ocimum urticifolium* Roth | Lamiaceae | Shrub | Abushafi | Leaf | Fresh | Dried in sun and ground and powder is mixed with water taken orally before breakfast |
| YA 010 | Arensilana | Dysentery, Green-yellow eye, fever | *Fagonia schweinfurthii* Hadidi | Zygophyllaceae | Shrub | Urinahala | Leaf, bark and root | Fresh | Pounded and macerated in water and filtrate is taken orally three times per day for seven days; the residues is used to wash body |
| YA 018 | Amogela | Madness, schizophrenic | *Ocimum spicatum* Defl. | Lamiaceae | Shrub | Balu | Root | Fresh | Ground and powder is taken orally in morning and inhales smoke at night covered |
| YA 100 |  |  | *Solanum incanum* L. | Solanaceae | Shrub | Wakrikoso | upper part | Fresh |  |
| YA 045 | Kida | Coughing | *Acacia oerfota* (Forssk.) Schweinf. | Fabaceae | Tree | Garomto | bark | Fresh | Crushed and mixed with water and filtrate is taken orally |
| YA 019 | Baro Hali | Snake bite | *Acalypha indica* L. | Euphorbiaceae | Herb | Baroberberie | whole plant | Fresh | Crushed and mixed water and filtrate is taken orally three times per day and residue is sniffed and used for body washing |
| YA 054 | Baro Hali | Snake bite | *Thymus schimperi* Ronniger | Lamiaceae | Herb | Harmela | whole plant | Fresh | Crushed and mixed water and filtrate is taken orally three times per day and wash body with residue |
| YA 082 | Begurubo/ hurara | Dysentery | *Senna alexandrina* Mill. | Fabaceae | Shrub | Senoyta | whole plant | Fresh | Dried in sun Crushed and boiled in water until mixture turned black and next morning 1 cup filtrate is taken orally before breakfast and 6 hours latter milk or honey is taken as antidote for 2 days |
| YA 008 | Begurubo/ hurara | Dysentery | *Balanites rotundifolia* (Van Tiegn.) Blatter | Balanitaceae | Shrub | Alayto | leaf | Fresh | Crushed and macerated in water overnight and filtrate is taken with small salt in the morning before meal three or four time per day |
| YA 008 | Lahidelea | Swelling/mumps on body and face | *Balanites rotundifolia* (Van Tiegn.) Blatter | Balanitaceae | Shrub | Alayto | Root | Fresh | Pounded and macerated in water and filtrate is taken orally and through nostril per day for seven days; the residue is used to wash body and applied on swollen part |
| YA 052 | Geratokmenum | Fire burn | *Abutilon figarianum* Guill. & Perro | Malvaceae | Herb | Hamokto | Leaf | Fresh | Dried and ground mixed water and applied topically on burned part and filtrate is taken in morning at evening before meal for 3 days |
| YA 075 | Intydele | Eye injury | *Acacia mellifera* (Vahl) Benth. | Fabaceae | Tree | Merka ato | Leaf | Fresh | Crushed and mixed with water and filtrate is applied on eye |
| YA 077 | Alsam | Vomiting and dysentery | *Aerva javanica* (Burm.f) Schultes | Amaranthaceae | Herb | Olayto | Root | Fresh | Crushed and mixed with water and filtrate is taken orally two times for one day before meal |
| YA 013 | Geno | Vomiting blood through mouth and nose | *Indigofera oblongifolia* Forssk. | Fabaceae | Shrub | Ayrowegit (Male) | Leaf and root | Fresh | Crushed and mixed with water and filtrate is taken morning and evening orally for 3 days |
| YA 046 | Tumewea | Swelling infection | *Barleria homoiotrichia* C. B. Clarke | Acanthaceae | Shrub | Geselto | Fruit | Dry | Pounded, and powder mixed with water and paste is applied on swelling until burst |
| YA 077 | Dele | Wound | *Aerva javanica* (Burm.f) Schultes | Amaranthaceae | Herb | Olayto | Leaf | Fresh | Crushed and applied in the wound and remains for three days |
| YA 078 | Dele | Head Injury/ cut | *Cadaba farinosa* Forssk. | Capparidaceae | Shrub | Galikihna/ Qelqasha | Leaf | Fresh | Pounded leaf mixed with butter and malted with hot-red stone and applied in the wound every three days until cure |
| YA 066 | Geno | Fever, Mitch, stomachache, headache | *Commicarpus helenae* (J.A. Schultes) Meikle | Nyctaginaceae | Herb | Seati | Whole plant | Fresh | Crushed mixed with water and one glass of filtrate is taken orally and applied through nostril morning and evening for one day |
| YA 004 | Jini | Devil Disease | *Allium sativum* L. | Alliaceae | Herb | Adi Besila | Fruit | Fresh | Mixed, Pounded, and boiled and two glasses are taken orally until cured for week; *T*. *shimperi* is crushed with water and body is washed with filtrate; root is chewed and juice is swallowed; *C. rotundifolia* root and leaves is Crushed and put on hot charcoal and patient inhale the smoke covered. |
| YA 084 |  |  | *Lepidium sativum* L. | Brassicaceae | Herb | Shenfai | Fruit | Fresh |  |
| YA 096 |  |  | *Nigella sativa* L. | Ranunculaceae | Herb | Tetkemun (tikur Azmud) | Seed | Dry |  |
| YA 054 |  |  | *Thymus schimperi* Ronniger | Lamiaceae | Herb | Kono Gabulea | Seed | Fresh |  |
| YA 054 |  |  | *Thymus schimperi* Ronniger | Lamiaceae | Herb | Kono Gabulea | Root | Fresh |  |
| YA 080 |  |  | *Cissus rotundifolia* (Forssk.) Vahl | Vitaceae | Climber | Saero Saero | Root & Leaf | Fresh |  |
| YA 024 | Buta | Evil Eye | *Pergularia tomentosa* L. | Asclepiadaceae | Shrub | Buta Gibid/ Devil tree | Root | Fresh | Crushed and soaked in water overnight and washed his body with filtrate two times for 1 day |
| YA 048 | Yefreno | Impotence | *Grewia villosa* Willd. | Tiliaceae | Shrub | Abelayto | Root | Fresh | Chewing and swallowing the juice and mixed with water and filtrate is used for washing body three times for one day |
| YA 098 | Urina | Infant sickness, yellowish green dysentery | *Aloe trichosantha* Berger | Aloaceae | Shrub | Urina | Leaf | Fresh | Crushed with water and body is washed with filtrate and applied through nostril until cure about a week |
| YA 045 | Iyala | Bird sickness, thinning, dysentery | *Acacia oerfota* (Forssk.) Schweinf. | Fabaceae | Tree | Garomto | Root | Fresh | Crushed with water and one glass is taken orally and used to wash body for seven days |
| YA 103 | Geno | Sudden illness, Fever, Mitch | *Celosia polystachia* (Forssk.) C.C. Townsend | Amaranthaceae | Herb | Werabikela | Whole plant | Fresh | Pounded mixed with water and one glass filtrate is taken three times orally, applied through nostril and used for washing body |
| YA 090 | Iyala | Bird sickness, thinning, dysentery | *Sterculia africana* (Lour.) Fiori | Sterculiaceae | Tree | Tereri/ Anqua | Leaf | Fresh | Crushed mixed with water and one glass of filtrate is taken orally for seven days |
| YA 091 | Antidenabu | eye injury | *Acacia senegal* (L.) Wild. | Fabaceae | Tree | Tikil Beyta | Bark | Fresh | Crushed with water and filtrate is applied on eye for five days |
| YA 077 | Alsa | Stomachache, bloating, | *Aerva javanica* (Burm.f) Schultes | Amaranthaceae | Herb | Olayto | Root | Fresh | Crushed with water and one glass filtrate is taken orally three times per day for three days |
| YA 013 | Iyetiya | Infant sickness, dysentery, vomiting | *Indigofera oblongifolia* Forssk. | Fabaceae | Shrub | Ayrowegit (Male) | Leaf | Fresh | Crushed with water and one glass filtrate is taken orally, body is washed with filtrate and applied through nostril for three days |
| YA 008 | Begifaye | Infant stomachache bloating | *Balanites rotundifolia* (Van Tiegn.) Blatter | Balanitaceae | Shrub | Alayto | Root | Fresh | Crushed with water and one glass filtrate is given orally for one day |
| YA 077 | Alsa | Stomachache, bloating, dysentery | *Aerva javanica* (Burm.f) Schultes | Amaranthaceae | Herb | Olayto | Root | Fresh | Chewing and swallowing the juice for one day |
| YA 019 | Asso | Malaria | *Acalypha indica* L. | Euphorbiaceae | Herb | Baroberberie | Leaf | Fresh | Crushed with water and 1 lt filtrate is taken orally until vomit green color matter |
| YA 003 | Denabu | Eye sickness, redness | *Cadaba rotundifolia* Forssk | Capparidaceae | Shrub | Dena behara | Leaf | Fresh | Crushed covered with cloth and filtrate is added as an eye drops for three days |
| YA 034 | Barohali | snake bite | *Cadaba glandulosa* Forssk. | Capparidaceae | Shrub | Se ula | Leaf and root | Fresh | Crushed with water and one glass filtrate is taken orally morning and evening and wash wound; root is Crushed and fluid is applied through nostril for one day |
| YA 033 | Dahwas | Obstruction to urinate, Venereal diseases (syphilis) | *Becium filamentosum* (Forssk.) Chiov. | Lamiaceae | Shrub | Dedeb | Fruit | Fresh | Crushed with water and filtrate is applied through nostril |
| YA 033 | Delay selo | Obstruction to birth labour | *Becium filamentosum* (Forssk.) Chiov. | Lamiaceae | Shrub | Halmanto | Branch | Fresh | Crushed with water and poured topically on the head of laboring woman and the infant comes out |
| YA 053 | Uriwekili | Retained Placenta | *Cocculus pendulus* (J. R. & G. Forst) Diels | Menispermaceae | Climber | Hayuka | Leaf | Fresh | Crushed with water one coffee cup filtrate is taken orally and body massage and placenta is expelled |
| YA 045 | Ametaki | Devil Disease | *Acacia oerfota* (Forssk.) Schweinf. | Fabaceae | Tree | Garomto | Root and leaf | Fresh | Mixed, Crushed with water and one glass filtrate is taken orally until cured for week and put in hot charcoal and patient inhales smoke |
| YA 076 | Undufeyta | mouth and upper part infection | *Plicosepalus robustus* Wiens & Polhil | Loranthaceae | Shrub | Merka ato/ Atokele | Leaf | Fresh | Crushed with water and applied topically on wound, one glass is taken orally and applied through nostril for seven days |
| YA 098 | Hemaki | Infant sickness, body wound, green dysentery, eye sickness | *Aloe trichosantha* Berger | Aloaceae | Shrub | Urina | Leaf | Fresh | Crushed with water and filtrate is taken orally and through nostril and wash body with residue three times per day for seven days |
| YA 064 | Iyala | Bird's sickness, infant sickness | *Euphorbia triaculeata* Forssk. | Euphorbiaceae | Shrub | Mad alto | Leaf | Fresh | Crushed with water and applied through nostrils and wash body with residue for seven days |
| YA 021 | Utuka | Fever, Mitch | *Ruellia patula* Jacq. | Acanthaceae | Shrub | Boboyta | Leaf | Fresh | Crushed with water and filtrate is applied through nostril three times for seven days |
| YA 083 | Duduba | Swelling around neck, | *Senna italica* Mill. | Fabaceae | Herb | Senoyta | Root | Fresh | Crushed with water and one coffee cup filtrate is given orally and applied through nostril for three days |
| YA 047 | Mudunta | Chest Pain | *Hibiscus vitifolius* L. | Malvaceae | Shrub | Gorbi | Root | Fresh | Cut to seven equal small size and tied around waist |
| YA 044 | Beteta | Hand figure swelling/ infection, Leprosy | *Cissus quadrangularis* L. | Vitaceae | Climber | Gemele | Leaf | Fresh | Crushed with hand and juice is applied on infected figure until the swollen part bursts and cured |
| YA 078 | Dulina hara | Head injury or cut | *Cadaba farinosa* Forssk. | Capparidaceae | Shrub | Gali Kehina | Leaf | Fresh | Crushed and boiled in water; when cold the wound is washed everyday until cure |
| YA 053 | Dulina hara | Feet injury/wound | *Cocculus pendulus* (J. R. & G. Forst) Diels | Menispermaceae | Climber | Hayuka | Stem | Fresh | Charred, powdered and mixed with butter and inserted into the wound every three days until cure |
| YA 075 | Dulina hara | Head injury, wound | *Acacia mellifera* (Vahl) Benth. | Fabaceae | Tree | Merka ato | Stem | Fresh | Charred, powdered and inserted in the wound every three days until cure |
| YA 075 | Medaat | Pelvic pain | *Acacia mellifera* (Vahl) Benth. | Fabaceae | Tree | Merka ato | Leaf | Dry | dried, ground and powder mixed with butter and heated by inserting hot stone into mixture and massage side of chest and back for 3 days |
| YA 084 | Iyala | Child disease, blood dysentery | *Lepidium sativum* L. | Brassicaceae | Herb | Shinfaye | Fruit | Dry | Mixed, dried, ground and powder mixed with water and taken orally and through nostril early morning for 7 days |
| YA 086 |  |  | *Acalypha fruticosa* Forssk. | Euphorbiaceae | Shrub | Subahsila | Leaf | Fresh |  |
| YA 008 |  |  | *Balanites rotundifolia* (Van Tiegn.) Blatter | Balanitaceae | Shrub | Alayto | Leaf | Fresh |  |
| YA 098 | Asso | Malaria | *Aloe trichosantha* Berger | Aloaceae | Shrub | Urae | Leaf | Fresh | Pounded mixed with water and macerated overnight 1 glass filtrate is taken orally before breakfast for 3 / 7 days |
| YA 034 | Geno | Fever, green eye | *Cadaba glandulosa* Forssk. | Capparidaceae | Shrub | Dunelita/ sulia | Leaf | Fresh | Pounded with water; macerated overnight and 1 glass filtrate is taken orally before breakfast for 3 days and wash body with residue |
| YA 103 | Geno | Fever, green eye | *Celosia polystachia* (Forssk.) C.C. Townsend | Amaranthaceae | Herb | Werabikela | Leaf | Fresh | Pounded with water and 1 glass filtrate is taken orally in morning and bed time for 3 days |
| YA 086 |  |  | *Acalypha fruticosa* Forssk. | Euphorbiaceae | Shrub | Subahsila | Leaf | Fresh |  |
| YA 019 |  |  | *Acalypha indica* L. | Euphorbiaceae | Herb | Baroberberie | Leaf | Fresh |  |
| YA 078 | Gosom | Flue | *Cadaba farinosa* Forssk. | Capparidaceae | Shrub | Ormayto | Leaf | Fresh | Pounded and boiled with water; inhale fume covered with blanket |
| YA 062 |  |  | *Grewia erythraea* Schweinf. | Tiliaceae | Shrub | Adayto | Leaf | Fresh |  |
| YA 003 |  |  | *Cadaba rotundifolia* Forssk | Capparidaceae | Shrub | Adengeli | Leaf | Fresh |  |
| YA 033 | Mayfelea | Eye Infection, blinds eye | *Becium filamentosum* (Forssk.) Chiov. | Lamiaceae | Shrub | Waido/ Bebto | Thorne and Leaf | Fresh | Rubbed with thorn and Crushed leaf is applied on kusil for three days |
| YA 016 | Geno | Thinning, fever, coughing blood through nose and mouth | *Boscia coriacea* Pax. | Capparidaceae | Tree | Aytinaeba | Leaf | Fresh | Mixed, Pounded and mixed with water and filtrate is taken orally in morning and evening for two days |
| YA 086 |  |  | *Acalypha fruticosa* Forssk. | Euphorbiaceae | Shrub | Subahsila | Leaf | Fresh |  |
| YA 008 | Utuka/Gosom | Flue, cough | *Balanites rotundifolia* (Van Tiegn.) Blatter | Balanitaceae | Shrub | Alayto | Leaf | Fresh | Mixed, Crushed and dried and put on red-hot charcoal and inhaling smoke covered with close |
| YA 016 |  |  | *Boscia coriacea* Pax. | Capparidaceae | Tree | Horma | Leaf | Fresh |  |
| YA 003 |  |  | *Cadaba rotundifolia* Forssk | Capparidaceae | Shrub | Adengeli | Leaf | Fresh |  |
| YA 086 |  |  | *Acalypha fruticosa* Forssk. | Euphorbiaceae | Shrub | Subahsila | Leaf | Fresh |  |
| YA 049 | Antibukiya | White on eye | *Heliotropium longiflorum* (ADC. in DC.) Jaub. & Spach. | Boraginaceae | Herb | Hadgento | Bark | Dry | Dried bark is ground and sieved and mixed with water and paste is applied on eye or powder with goat milk is applied on the eye morning and evening for 2 days |
| YA 015 | Alsam | Food poisoning | *Indigofera articulata* Gouan | Fabaceae | Shrub | Ayrowegit | Root | Fresh | Pounded and mixed with water and filtrate is taken orally in morning and at evening for two days in place of water |
| YA 067 | Harero | Fire burn | *Selaginella kraussiana* (Kunze) A.Braun | Selaginellaceae | Herb | Kuraniba | Whole plant | Fresh | Charred, powdered and mixed with butter and applied topically on the body |
| YA 028 | Dahowaso | bladder blocking | *Cucumis prophetarum* L. | Cucurbitaceae | Climber | Denkekebis | Whole plant | Fresh | Pounded, mixed with water and filtrate is taken in morning and evening for three days |
| YA 048 | Anderoyta | Jaundice | *Grewia villosa* Willd. | Tiliaceae | Shrub | Habeleyta | Root | Fresh | Root is Crushed and mixed with water; if affected when a patient look at it turns green; then whole plant is Pounded and dried, mixed with butter is given through nostril and orally in the morning for three days |
| YA 048 |  |  | *Grewia villosa* Willd. | Tiliaceae | Shrub | Habeleyta | Whole plant | Dry |  |
| YA 087 |  |  | *Aristolochia bracteolata* Lam. | Aristolochiaceae | Herb | Suie Suie | Whole plant | Dry |  |
| YA 087 | Delila | Kolakusil | *Aristolochia bracteolata* Lam. | Aristolochiaceae | Herb | Suie Suie | Whole plant | Dry | Pounded and powder is applied on the wound for 4 days |
| YA 064 | Ayroboda | Unable to urinate, Venereal | *Euphorbia triaculeata* Forssk. | Euphorbiaceae | Shrub | Ingdato | Stem and root | Fresh | Pounded and mixed with water and filtrate is taken orally once |
| YA 008 | Asso | Malaria | *Balanites rotundifolia* (Van Tiegn.) Blatter | Balanitaceae | Shrub | Alayto | Leaf | Fresh | Mixed, Pounded and mixed in water/milk and filtrate is orally taken in the morning and evening for 3 days |
| YA 001 |  |  | *Ocimum urticifolium* Roth | Lamiaceae | Shrub | Abushafi | Whole plant | Fresh |  |
| YA 019 |  |  | *Acalypha indica* L. | Euphorbiaceae | Herb | Baroberberie | Whole plant | Fresh |  |
| YA 013 | Alsa | Dysentery | *Indigofera oblongifolia* Forssk. | Fabaceae | Shrub | Ayrowegit (Male) | Leaf | Fresh | Mixed , Crushed and mixed water and filtrate is given orally in the morning and evening for 4 days |
| YA 095 |  |  | *Balanites aegyptiaca* (van Tieghem) Blatter | Balanitaceae | Tree | Udayto | Leaf and bark | Fresh |  |
| YA 055 | Kida | Dry Cough, Asthma | *Seddera hirsute* Dammer ex Hall. f. | Convolvulaceae | Shrub | Harmela | Whole plant | Fresh | Crushed and taken with honey |
| YA 055 | Kidu | Impotence | *Seddera hirsute* Dammer ex Hall. f. | Convolvulaceae | Shrub | Harmela | Whole plant | Fresh | Crushed and boiled in goat milk and filtrate is taken orally |
| YA 097 | Kida | Swelling of glands, neck | *Solanum marginatum* Lf | Solanaceae | Shrub | Angulea | Leaf | Fresh | Crushed with water and 1 glass filtrate is taken in the morning and evening for three days; residue is used for washing body |
| YA 086 |  |  | *Acalypha fruticosa* Forssk. | Euphorbiaceae | Shrub | Subahsila | Leaf | Fresh |  |
| YA 083 |  |  | *Senna italica* Mill. | Fabaceae | Herb | Senoyta | Leaf | Fresh |  |
| YA 034 |  |  | *Cadaba glandulosa* Forssk. | Capparidaceae | Shrub | Dunelita | Leaf | Fresh |  |
| YA 075 | Gosom | Flue | *Acacia mellifera* (Vahl) Benth. | Fabaceae | Tree | Merka ato | Bark | Fresh | Internal part of bark is chewed and juice is swallowed |
| YA 106 | Kandela | internal parasites | *Ocimum basilicum* L. | Lamiaceae | Herb | Yagali | Leaf | Fresh | Crushed and mixed with water 1 glass is taken orally once |
| YA 054 | Kandela | Amoeba Tapeworm ascaris | *Thymus schimperi* Ronniger | Lamiaceae | Herb | Harmela Kuba | Root | Fresh | Crushed, boiled and filtrate mixed with honey is taken orally |
| YA 082 | Urufea | Gastritis mouth, tongue and gum inflammation | *Senna alexandrina* Mill. | Fabaceae | Shrub | Aliseno | Leaf | Fresh | Crushed and filtrate is taken orally in the morning and evening for 3 days |
| YA 040 | Buta Merihara | Evil eye | *Withania somnifera* (L.) Dunal | Solanaceae | Shrub | Buta Meihara | Root | Fresh | Crushed sniffed and mixed with water filtrate is taken orally for three days |
| YA 008 | Dewao | Child fifth year Teeth | *Balanites rotundifolia* (Van Tiegn.) Blatter | Balanitaceae | Shrub | Alayto | Stem | Fresh | Burned stem is used to torch gum |
| YA 034 | Geno | Dysentery, Green-yellow eye, fever | *Cadaba glandulosa* Forssk. | Capparidaceae | Shrub | Dunelita /sule/ | Leaf | Fresh | Crushed and mixed in water and filtrate is taken orally for three days |
| YA 069 | Ali | snake bite | *Ziziphus spina-christi* (L.) Desf. | Rhamnaceae | Tree | Gali Ali | Leaf and root | Fresh | Pounded and mixed with water and filtrate is taken in place of water for one week |
| YA 086 | Kida | Breast swelling, infection | *Acalypha fruticosa* Forssk. | Euphorbiaceae | Shrub | Subahsila | Leaf | Fresh | Pounded and mixed with water and filtrate is taken orally once |
| YA 013 |  |  | *Indigofera oblongifolia* Forssk. | Fabaceae | Shrub | Ayrowegit (Male) | Leaf | Fresh |  |
| YA 035 |  |  | *Acacia tortilis* (Forssk.) Schweinf. | Fabaceae | Tree | Eabeto | Leaf | Fresh |  |
| YA 065 |  |  | *Bourreria orbicularis* (Hutch. & E.A. Bruce) Thulin | Boraginaceae | Shrub | Inguleyta | Leaf | Fresh |  |
| YA 104 |  |  | *Silene macrosolen* A. Rich. | Caryophyllaceae | Herb | Werasibila | Leaf | Fresh |  |
| YA 068 |  |  | *Ziziphus mauritiana* Lam. | Rhamnaceae | Tree | Kusra | Leaf | Fresh |  |
| YA 078 |  |  | *Cadaba farinosa* Forssk. | Capparidaceae | Shrub | Ormayto | Leaf | Fresh |  |
| YA 008 |  |  | *Balanites rotundifolia* (Van Tiegn.) Blatter | Balanitaceae | Shrub | Alayto | Leaf | Fresh |  |
| YA 013 | Geno | Fever, tiredness, | *Indigofera oblongifolia* Forssk. | Fabaceae | Shrub | Ayrowegit (Male) | Leaf | Fresh | Mixed, Crushed with water and one glass filtrate is given orally in morning and evening for one day |
| YA 034 |  |  | *Cadaba glandulosa* Forssk. | Capparidaceae | Shrub | Dunelita | Leaf | Fresh |  |
| YA 008 |  |  | *Balanites rotundifolia* (Van Tiegn.) Blatter | Balanitaceae | Shrub | Alayto | Leaf | Fresh |  |
| YA 062 |  |  | *Grewia erythraea* Schweinf. | Tiliaceae | Shrub | Adayto | Leaf | Fresh |  |
| YA 097 |  |  | *Solanum marginatum* Lf | Solanaceae | Shrub | Ungule | Leaf | Fresh |  |
| YA 086 |  |  | *Acalypha fruticosa* Forssk. | Euphorbiaceae | Shrub | Subahsila | Leaf | Fresh |  |
| YA 064 | Hadad | Stomach bloating | *Euphorbia triaculeata* Forssk. | Euphorbiaceae | Shrub | Ingda ato | Latex | Fresh | Mixed with goat milk or water one glass is given orally for one day |
| YA 081 | Diglo | Bone breakage | *Parkinsonia scioana* (Chiov.) Brenan | Fabaceae | Shrub | Sekuhto | Root | Fresh | Mixed, Crushed and mixed with sheep blood tied on broken bone area and removed after two days |
| YA 062 |  |  | *Grewia erythraea* Schweinf. | Tiliaceae | Shrub | Hudayto | Root | Fresh |  |
| YA 086 | Ganow | Bone breakage | *Acalypha fruticosa* Forssk. | Euphorbiaceae | Shrub | Subahsila | Leaf | Fresh | Crushed and applied on the area of broken bone as treatment for fever |
| YA 008 | Geno | Headache, vomiting, dysentery | *Balanites rotundifolia* (Van Tiegn.) Blatter | Balanitaceae | Shrub | Alayto | Leaf | Fresh | Mixed, Crushed with water and one glass filtrate is given orally and through nostril until cure |
| YA 078 |  |  | *Cadaba farinosa* Forssk. | Capparidaceae | Shrub | Ormayto | Leaf | Fresh |  |
| YA 086 |  |  | *Acalypha fruticosa* Forssk. | Euphorbiaceae | Shrub | Subahsila | Leaf | Fresh |  |
| YA 019 |  |  | *Acalypha indica* L. | Euphorbiaceae | Herb | Baroberberie | Leaf | Fresh |  |
| YA 033 |  |  | *Becium filamentosum* (Forssk.) Chiov. | Lamiaceae | Shrub | Dew dew | Leaf | Fresh |  |
| YA 090 | Aweita | Swelling on skin | *Sterculia africana* (Lour.) Fiori | Sterculiaceae | Tree | Tetehara | Leaf | Fresh | Crushed and applied on the area of swollen skin for two day until burst |
| YA 066 | Utuka | headache on one side of head | *Commicarpus helenae* (J.A. Schultes) Meikle | Nyctaginaceae | Herb | Kerebto | Root | Fresh | Crushed mixed with water and juice is applied through nostril morning and evening for one day |
| YA 034 | Alsa | Stomach bloating, vomiting, fever | *Cadaba glandulosa* Forssk. | Capparidaceae | Shrub | Dunelita | Leaf | Fresh | Crushed with water and one glass filtrate is given three times for one day |
| YA 009 | Bekaie | Head injury | *Heliotropium cinerascens* DC. & A. DC. | Boraginaceae | Herb | Amaeda | Leaf | Fresh | Mixed, Crushed and mixed with goat butter and topically applied on injured part for three days |
| YA 089 |  |  | *Aizoon canariensis* L. | Aizoaceae | Herb | Taasu | Leaf | Fresh |  |
| YA 097 |  |  | *Solanum marginatum* Lf | Solanaceae | Shrub | Ungule | Leaf | Fresh |  |
| YA 040 | Awi ata | Swelling on skin | *Withania somnifera* (L.) Dunal | Solanaceae | Shrub | Burahin Hadda | Leaf | Fresh | Crushed and applied on swollen part until burst and continues for two days |
| YA 039 | Anderoyta | yellow Eye and skin | *Acacia seyal* Del. | Fabaceae | Tree | Galielea | Leaf | Fresh | Crushed with water and one glass filtrate is given once per week for one month |
| YA 012 | Ulahama | swelling on body | *Rhus natalensis* Krauss | Anacardiaceae | Tree | Atimi | Leaf | Fresh | Crushed with water and two glasses filtrate is given orally every two days four times |
| YA 008 | Kida | Swelling and wound on breast and neck | *Balanites rotundifolia* (Van Tiegn.) Blatter | Balanitaceae | Shrub | Alayto | Leaf | Fresh | Mixed, Crushed with water and one glass filtrate is given for patien if not cured treatment is repeated again |
| YA 086 |  |  | *Acalypha fruticosa* Forssk. | Euphorbiaceae | Shrub | Subahsila | Leaf | Fresh |  |
| YA 040 |  |  | *Withania somnifera* (L.) Dunal | Solanaceae | Shrub | Galikihna | Leaf | Fresh |  |
| YA 103 |  |  | *Celosia polystachia* (Forssk.) C.C. Townsend | Amaranthaceae | Herb | Werabikela | Leaf | Fresh |  |
| YA 066 |  |  | *Commicarpus helenae* (J.A. Schultes) Meikle | Nyctaginaceae | Herb | Kerebto | Leaf | Fresh |  |
| YA 075 |  |  | *Acacia mellifera* (Vahl) Benth. | Fabaceae | Tree | Galielea | Leaf | Fresh |  |
| YA 008 | Asso | Malaria | *Balanites rotundifolia* (Van Tiegn.) Blatter | Balanitaceae | Shrub | Alayto | Leaf | Fresh | Mixed, Pounded and mixed with water and 1 glass filtrate is taken orally for seven days |
| YA 019 |  |  | *Acalypha indica* L. | Euphorbiaceae | Herb | Baroberberie | Leaf | Fresh |  |
| YA 099 | Utuka | Fever, headache | *Priva curtisiae* Kobuski | Verbenaceae | Herb | Utuka Hara | Leaf | Fresh | Pounded and the juice is applied through nostrils |
| YA 008 | Utuka | Fever, headache | *Balanites rotundifolia* (Van Tiegn.) Blatter | Balanitaceae | Shrub | Derbulea | Leaf | Fresh | Crushed and juice is applied through nostrils |
| YA 008 | Utuka | Fever, headache | *Balanites rotundifolia* (Van Tiegn.) Blatter | Balanitaceae | Shrub | Alayto | Root | Fresh | Pounded and juice is applied through nostrils |
| YA 019 | Asso | Malaria | *Acalypha indica* L. | Euphorbiaceae | Herb | Baroberberie | Leaf | Fresh | Pounded and mixed with milk 1 cup is taken at night for three days |
| YA 008 |  |  | *Balanites rotundifolia* (Van Tiegn.) Blatter | Balanitaceae | Shrub | Alayto | Leaf | Fresh |  |
| YA 078 | Gosom | Flue | *Cadaba farinosa* Forssk. | Capparidaceae | Shrub | Ormayto | Leaf | Fresh | Crushed and sniffed through nostrils |
| YA 034 | Gano | Lung infection | *Cadaba glandulosa* Forssk. | Capparidaceae | Shrub | Dunelita | Leaf | Fresh | Pounded and mixed with water and filtrate is taken orally for three days |
| YA 008 | Urina Hara | Milk teeth dysentery | *Balanites rotundifolia* (Van Tiegn.) Blatter | Balanitaceae | Shrub | Gini meaydu | Leaf | Fresh | Crushed and soaked in water and filtrate is given orally and through nostrils three time per day for 3 days. the residue is used to wash breast of mother |
| YA 045 | Lahasito | infection, Rush on body, black skin | *Acacia oerfota* (Forssk.) Schweinf. | Fabaceae | Tree | Totogara /Timb Zaf, Mit Zaf/ | Leaf | Fresh | Leaves are roasted, powdered and mixed with butter applied topically for seven days |
| YA 019 | Asso | Malaria | *Acalypha indica* L. | Euphorbiaceae | Herb | Baroberberie | Leaf | Fresh | Crushed and soaked in water and filtrate is given orally |
| YA 064 | Hurar | Food poisoning, internal parasite | *Euphorbia triaculeata* Forssk. | Euphorbiaceae | Shrub | Ingdato/ qinchib/ | Leaf latex | Fresh | Crushed leaf Latex is mixed with goat milk taken early morning orally once; no food |
| YA 019 | Asso | Malaria | *Acalypha indica* L. | Euphorbiaceae | Herb | Baroberberie | Leaf | Fresh | Crushed filtrate is taken orally and milk is given as antidote |
| YA 069 | Mujunta | Chest and side pain | *Ziziphus spina-christi* (L.) Desf. | Rhamnaceae | Tree | Gale ale | Root | Fresh | Chewing and swallowing the juice in the morning before meal for 1 day |
| YA 082 | Geno | Eye -yellow, fever, restlessness | *Senna alexandrina* Mill. | Fabaceae | Shrub | Sole his | Bark | Fresh | Pounded and mixed with water and filtrate is given three times for 1 day |
| YA 028 | Intydinabite | Eye injury | *Cucumis prophetarum* L. | Cucurbitaceae | Climber | Daknbihara | upper part | Fresh | Crushed and mixed with water and filtrate is applied as eye-drop three times for one day |
| YA 103 | Geno | sudden illness, Fever, Mitch, stomachache, vomiting, dysentery | *Celosia polystachia* (Forssk.) C.C. Townsend | Amaranthaceae | Herb | Werabikela | leaf | Fresh | Crushed and macerated in water and filtrate is taken orally and through nostrils morning and evening for seven days |
| YA 086 |  |  | *Acalypha fruticosa* Forssk. | Euphorbiaceae | Shrub | Subahsila | leaf | Fresh |  |
| YA 043 |  |  | *Calotropis procera* (Ait.) Ait.f. | Apocynaceae | Shrub | Gela ato | leaf | Fresh |  |
| YA 078 |  |  | *Cadaba farinosa* Forssk. | Capparidaceae | Shrub | Galikihna | leaf | Fresh |  |
| YA 034 |  |  | *Cadaba glandulosa* Forssk. | Capparidaceae | Shrub | Dunelita | leaf | Fresh |  |
| YA 006 |  |  | *Plumbago zylanica* L. | Plumbaginaceae | Herb | Aftitosh | leaf | Fresh |  |
| YA 083 | Alsam | Vomiting and dysentery | *Senna italica* Mill. | Fabaceae | Herb | Senoyta | leaf | Fresh | Crushed and macerated in water and filtrate is taken orally and through nostrils morning and evening for four days |
| YA 062 |  |  | *Grewia erythraea* Schweinf. | Tiliaceae | Shrub | Hidayto | leaf | Fresh |  |
| YA 078 | Afaado | Heart disease, breathing problem, dysentery | *Cadaba farinosa* Forssk. | Capparidaceae | Shrub | Galikihna/andera | Leaf | Fresh | Crushed and macerated in water and taken orally morning and evening for 2 days |
| YA 019 | Geno | Blood cough | *Acalypha indica* L. | Euphorbiaceae | Herb | Baroberberie | Leaf | Fresh | Crushed and mixed in water and filtrate is given orally, morning for 3 days |
| YA 103 |  |  | *Celosia polystachia* (Forssk.) C.C. Townsend | Amaranthaceae | Herb | Werabikela | Young part | Fresh |  |
| YA 034 |  |  | *Cadaba glandulosa* Forssk. | Capparidaceae | Shrub | Demkin har/ Dunelita/ | Leaf | Fresh |  |
| YA 008 | Asso | Malaria | *Balanites rotundifolia* (Van Tiegn.) Blatter | Balanitaceae | Shrub | Alayto | Leaf | Fresh | Crushed and macerated over night and filtrate is taken orally in morning for seven days |
| YA 013 | Dinketa | Chest pain | *Indigofera oblongifolia* Forssk. | Fabaceae | Shrub | Ayrowegit (Male) | Root | Fresh | Mixed, Crushed and mixed with water and 1 glass filtrate is taken orally in the morning for three days |
| YA 073 |  |  | *Kanahia laniflora* (Forssk.) R.Br. | Asclepiadaceae | Shrub | Malikinori | Root | Fresh |  |
| YA 013 | Halib | Food Poisoning | *Indigofera oblongifolia* Forssk. | Fabaceae | Shrub | Ayrowegit (Male) | Root | Fresh | Crushed and mixed with water and filtrate is taken orally in the morning for 4 days |
| YA 099 | Utuka | Mitch | *Priva curtisiae* Kobuski | Verbenaceae | Herb | Utuka hara | Leaf | Fresh | Crushed leaf is inserted in the nostrils in the evening for 3 days |
| YA 042 | Kida | Breast swelling, infection, neck swelling & arm pits | *Dobera glabra* (Forssk.) Poir. | Salvadoraceae | Shrub | Mudu udo | Leaf | Fresh | Crushed mixed with water and filtrate is taken two times per day morning and evening for seven days. |
| YA 078 |  |  | *Cadaba farinosa* Forssk. | Capparidaceae | Shrub | Ormayto | Stem | Fresh |  |
| YA 009 | Likek | Leg joint sickness, unable to walk | *Heliotropium cinerascens* DC. & A. DC. | Boraginaceae | Herb | Hara/ Amada | Stem | Fresh | Stems are washed with salt water and are inserted with salt into the cut made at the bulged area of foot and joints, After three days it burst and heal |
| YA 008 |  |  | *Balanites rotundifolia* (Van Tiegn.) Blatter | Balanitaceae | Shrub | Alayto | Stem | Fresh |  |
| YA 075 | Denabu | Eye sickness | *Acacia mellifera* (Vahl) Benth. | Fabaceae | Tree | Merka ato | Leaf | Fresh | Crushed with water and filtrate is used as eye drop at bed time for three days |
| YA 059 | Ibibyak | Swelling of foot | *Lawsonia inermis* L. | Lythraceae | Tree | Hina/andera | Leaf | Fresh | Crushed and tied on swollen foot at night |
| YA 103 | Geno | Sudden illness, fever, dysentery, vomiting | *Celosia polystachia* (Forssk.) C.C. Townsend | Amaranthaceae | Herb | Werabikela | Leaf | Fresh | Crushed and filtrate is given orally and through nostril morning and evening for three days |
| YA 019 |  |  | *Acalypha indica* L. | Euphorbiaceae | Herb | Baroberberie | Whole plant | Fresh | If not cured whole plant is Crushed and filtrate is given orally and through nostril morning and evening for three days |
| YA 063 |  |  | *Tamarindus indica* L. | Fabaceae | Tree | Hura | Bark | Fresh | If not cured bark is Crushed and macerated for few hours in water and filtrate is give for two days orally about 50 ml |
| YA 103 | Tumewea | Swelling on skin and wound | *Celosia polystachia* (Forssk.) C.C. Townsend | Amaranthaceae | Herb | Werabikela | Whole plant | Fresh | Crushed with water and one glass filtrate is given orally and through nostril, Dunelita leaf Crushed with water and one coffee cup filtrate is given orally in morning and evening for seven days and body is washed with residue |
| YA 034 |  |  | *Cadaba glandulosa* Forssk. | Capparidaceae | Shrub | Dunelita | Leaf | Fresh |  |
| YA 106 | Tumewea | Swelling on skin and wound | *Ocimum basilicum* L. | Lamiaceae | Herb | Yemaru eta | whole plant | Fresh | Crushed with water and filtrate is applied topically on the swollen part for two days |
| YA 019 | Ladorea/Haryitya | Rush on body, fever, headache | *Acalypha indica* L. | Euphorbiaceae | Herb | Baroberberie | Leaf | Fresh | Crushed with water and one glass filtrate is given orally and through nostril for two day |
| YA 043 |  |  | *Calotropis procera* (Ait.) Ait.f. | Apocynaceae | Shrub | Gelato | Leaf | Fresh |  |
| YA 086 |  |  | *Acalypha fruticosa* Forssk. | Euphorbiaceae | Shrub | Subahsila | Leaf | Fresh |  |
| YA 095 |  |  | *Balanites aegyptiaca* (van Tieghem) Blatter | Balanitaceae | Tree | Udayto | Bark (Root) | Fresh |  |
| YA 014 | Baro Halibiak | Snake Bite | *Trianthema portulacastrum* L. | Aizoaceae | Herb | Aburea | whole plant | Fresh | Crushed with water and few drops are given through nostril for one day |
| YA 018 | Baro Halibiak | Snake Bite | *Ocimum spicatum* Defl. | Lamiaceae | Shrub | Balo Balo | Root | Fresh | Pounded, mixed camel milk and chewed and juice is swallowed once for one day |
| YA 069 | Baro Halibiak | Snake Bite | *Ziziphus spina-christi* (L.) Desf. | Rhamnaceae | Tree | Kusra | Young branch | Fresh | Crushed with water and one glass filtrate is given per day for two days and wash body with residue |
| YA 017 | Sununie | Nose blooding | *Trigonella foenum-graecum* L. | Fabaceae | Herb | Bado Dala | Root | Fresh | Crushed with water and drops of filtrate is applied through nostril morning, midday, and evening for one day |
| YA 015 |  |  | *Indigofera articulata* Gouan | Fabaceae | Shrub | Mahula | Leaf and root | Fresh |  |
| YA 041 | Lahaw | Birth labour | *Acacia nilotica* (L.) Willd. Ex Del. | Fabaceae | Tree | Gansel | Root | Fresh | Crushed with water and drops of filtrate is given through nostril |
| YA 075 |  |  | *Acacia mellifera* (Vahl) Benth. | Fabaceae | Tree | Marka | Young branch | Fresh | Crushed with water and filtrate is given orally and wash body with residue for one day |
| YA 028 | Tola Abenum | Witchcraft disease | *Cucumis prophetarum* L. | Cucurbitaceae | Herb | Abenino Beyis | leaf and Young Branch | Fresh | Crushed With Water and filtrate is given orally, drops are applied through nostril morning and evening for one day and body is washed with residue |
| YA 073 |  |  | *Kanahia laniflora* (Forssk.) R.Br. | Asclepiadaceae | Shrub | Mardesa | Root | Fresh |  |
| YA 095 |  |  | *Balanites aegyptiaca* (van Tieghem) Blatter | Balanitaceae | Tree | Udayto | Bark | Fresh | Crushed with water and one glass is given morning and evening for one day |
| YA 016 | Berohim Dehito | Snake Bite | *Boscia coriacea* Pax. | Capparidaceae | Tree | Aytinaeba | Leaf, Fruit, | Fresh | Leaf Crushed with water and boiled with sugar and one coffee cup is given and root is Crushed and applied on wound |
| YA 053 |  |  | *Cocculus pendulus* (J. R. & G. Forst) Diels | Menispermaceae | Climber | Hayuka | Root | Fresh | Ground mixed with camel milk and taken orally for seven days |
| YA 018 |  |  | *Ocimum spicatum* Defl. | Lamiaceae | Shrub | Balo Balo | Root | Fresh | Ground mixed with camel milk or butter and taken orally for one day |
| YA 073 | Amatak | Devil Disease, Madness | *Kanahia laniflora* (Forssk.) R.Br. | Asclepiadaceae | Shrub | Malikinori | Whole plant | Fresh | Ground mixed with water and one liter is given orally and wash body for seven days |
| YA 089 |  |  | *Aizoon canariensis* L. | Aizoaceae | Herb | Taeisoyta | Whole plant | Fresh | Ground mixed with water and patient drinks for the whole day for one day |
| YA 037 | Lahaw | Skin bleaching/Lemti/ | *Dichrostachys cinerea* (L.) Wight & Am. | Fabaceae | Shrub | Gabeyta | Root | Fresh | Ground mixed with water and boiled, cooled filtrate is taken for the whole day |
| YA 030 | Bagemi | Stomach blotting, internal disease, fever | *Xanthium strumarium* L. | Asteraceae | Herb | Derbulea | Whole plant | Fresh | Ground mixed with water and two tea glasses are taken orally and drops through nostril for one day |
| YA 013 | Iyala | Infant sickness, dysentery, fever, stomach blotting | *Indigofera oblongifolia* Forssk. | Fabaceae | Shrub | Ayrowegit (Male) | Leaf | Fresh | Ground, mixed with water and one coffee cup is given orally, drops through nostril and wash body for three days |
| YA 063 | Gano | blooding nose, fever | *Tamarindus indica* L. | Fabaceae | Tree | Hura | Bark | Fresh | Ground mixed with water and boiled, cooled one tea cup is given orally and few drops through nostril three times for one day |
| YA 041 | Lahaw | Birth labour | *Acacia nilotica* (L.) Willd. Ex Del. | Fabaceae | Tree | Gansol | Root | Fresh | Ground and mixed with water and drops of filtrate is given through nostril |
| YA 086 | Amatak | Devil Disease, Madness | *Acalypha fruticosa* Forssk. | Euphorbiaceae | Shrub | Subahsila | Leaf | Fresh | Ground and mixed with water and three glasses filtrate is given orally for three days |
| YA 004 | Sisa Hitu | Skin infection | *Allium sativum* L. | Alliaceae | Herb | Ado besel | bulb | Fresh | Ground and applied topically on affected area of skin |
| YA 010 | Urina | Infant sickness, dysentery, fever, stomach blotting | *Fagonia schweinfurthii* Hadidi | Zygophyllaceae | Shrub | Urina hara | Leaf | Fresh | Ground mixed with water and one glass is given orally and wash body for three days |
| YA 103 | Gano | fever, headache, Mitch | *Celosia polystachia* (Forssk.) C.C. Townsend | Amaranthaceae | Herb | Werabikela | Leaf | Fresh | Ground mixed with water and one glass is given orally morning and evening for one day |
| YA 013 | Iyala | Infant disease, restlessness | *Indigofera oblongifolia* Forssk. | Fabaceae | Shrub | Ayrowegit (Male) | Root | Fresh | Ground mixed with water and one glass is given orally for one day |
| YA 013 | Urina | Infant dysentery | *Indigofera oblongifolia* Forssk. | Fabaceae | Shrub | Ayrowegit (Male) | Leaf | Fresh | Ground mixed with water and one glass is given orally, drops through nostril and wash body once per day for three days |
| YA 103 | Gano | blooding nose, fever | *Celosia polystachia* (Forssk.) C.C. Townsend | Amaranthaceae | Herb | Werabikela | Bark | Fresh | Pounded mixed with water and two glasses are given orally, drops through nostril and wash body for two days |
| YA 095 | Geno | Anthrax | *Balanites aegyptiaca* (van Tieghem) Blatter | Balanitaceae | Tree | Udayto | Root | Fresh | Mixed and Pounded together with water and applied through nostril and one cup is given orally for 1 to 3 days |
| YA 078 |  |  | *Cadaba farinosa* Forssk. | Capparidaceae | Shrub | Ormayto | Root | Fresh |  |
| YA 097 |  |  | *Solanum marginatum* Lf | Solanaceae | Shrub | Ungule | Leaf | Fresh |  |
| YA 074 | Bagemi | Stomach bloating | *Acacia ehrenbergiana* Hayne | Fabaceae | Tree | Mekeni | Bark | Fresh/dry | Dried and Pounded and mixed with water; one cup of filtrate is given orally for seven days |
| YA 078 | Ginyat/Medeyat | swelling of knee or hip | *Cadaba farinosa* Forssk. | Capparidaceae | Shrub | Ormayto | Root | Fresh | Mixed and Pounded and inserted in the cut made on the knee and fasten only one time |
| YA 062 |  |  | *Grewia erythraea* Schweinf. | Tiliaceae | Shrub | Adayto | Root | Fresh |  |
| YA 014 | Utuka | Eye swell, teeth is uprooted | *Trianthema portulacastrum* L. | Aizoaceae | Herb | Egeru | Whole plant | Fresh | Pounded mixed with water and filtrate is applied through nostril three times per day |
| YA 051 | Gosom | Flue | *Polygala obtusissima* Chod. | Polygalacea | Shrub | Halmela | Whole plant | Fresh | Pounded and put on hot charcoal and smoke is inhaled covered by blanket at night for one day |
| YA 055 | Tethara | Sleep waking | *Seddera hirsute* Dammer ex Hall. f. | Convolvulaceae | Shrub | Dathara | Whole plant | Fresh | Pounded and put on hot charcoal and smoke is inhaled covered by blanket at night for three days |
| YA 062 | Silaytu | Swelling on eye, stomach, buttock, and feet/ kintarot | *Grewia erythraea* Schweinf. | Tiliaceae | Shrub | Hudayto | Bark | Fresh | Pounded and mixed with butter and tied on swelling (kintarot) for three days |
| YA 026 |  |  | *Citrullus colocynthis* (L.) Schrad. | Cucurbitaceae | Climber | Taarto/Daarto | Stem | Fresh |  |
| YA 086 | Lahi Dalea | Wound, swelling, pus, fever | *Acalypha fruticosa* Forssk. | Euphorbiaceae | Shrub | Subahsila | Leaf | Fresh | Mixed, Pounded with water and filtrate is applied through nostril and residue is applied on the wound/injury for three days |
| YA 053 |  |  | *Cocculus pendulus* (J. R. & G. Forst) Diels | Menispermaceae | Climber | Hayuka | Leaf | Fresh |  |
| YA 003 |  |  | *Cadaba rotundifolia* Forssk | Capparidaceae | Shrub | Dengel | Leaf | Fresh |  |
| YA 082 | Barogiei | Hand injury/ inflammation | *Senna alexandrina* Mill. | Fabaceae | Shrub | Aliseno | Leaf | Fresh | Pounded mixed with milk and applied topically on wound until cure and pus dries |
| YA 046 | Utuka/ Undufeyta | inflammation of mouth and teeth | *Barleria homoiotrichia* C. B. Clarke | Acanthaceae | Shrub | Geselto | Bark | Fresh | Mixed and Pounded together with water and applied through nostril and one cup is given orally for 1 to 3 days |
| YA 066 |  |  | *Commicarpus helenae* (J.A. Schultes) Meikle | Nyctaginaceae | Herb | Kerebto | Root | Fresh |  |
| YA 063 |  |  | *Tamarindus indica* L. | Fabaceae | Tree | Hura | Bark | Fresh |  |
| YA 028 | Denabu | Eye injury | *Cucumis prophetarum* L. | Cucurbitaceae | Climber | Denkekebis | Leaf | Fresh | Pounded and mixed with water, filtrate is used as eye drop |
| YA 105 | Bekema Mara/Mudema Mara | hand injury/ bullet injury | *Oncocalyx g1abratus* (Engl.) M. Gilbert | Loranthaceae | Shrub | Wgorto/ Atokele | Stem and leaf | Fresh | Pounded together and mixed with butter and applied topically on the injured part for weeks until the broken head heals |
| YA 078 | Ginyat/ Medeyat | Knee /hip swelling | *Cadaba farinosa* Forssk. | Capparidaceae | Shrub | Ormayto | Leaf | Fresh | Pounded and mixed with blood and tied on swelling for one day |
| YA 051 | Ulahama/undero | Stomach bloating | *Polygala obtusissima* Chod. | Polygalacea | Shrub | Halmela | Root | Fresh | Pounded and macerated in water for a night, filtrate is taken orally before meal for three day in the morning |
| YA 050 | Kudu Hara | Impotence | *Seddera bagshawei* Rendle | Convolvulaceae | Shrub | Halemagayra | Root | Fresh | Chewing root and swallow the juice |
| YA 102 | Abeshi | Snake Bite | *Olea europaea L. subsp. cuspidata* (Wall.ex G. Don) Cif. | Oleaceae | Tree | Wegorto | Root | Fresh | Root is pounded and applied on swollen below knee part until cure; leaf is Pounded, mixed with water and boiled, and filtrate is given orally before meal in morning for five days (poison swells stomach) |
| YA 003 |  |  | *Cadaba rotundifolia* Forssk | Capparidaceae | Shrub | Adengeli | Leaf | Fresh |  |
| YA 051 | Alsa/Halib | Food Poisoning | *Polygala obtusissima* Chod. | Polygalacea | Shrub | Halmela | Root | Fresh | Pounded and mixed water and 1 cup filtrate is taken three times per day for three days |
| YA 052 | Denabu | Eye injury | *Abutilon figarianum* Guill. & Perro | Malvaceae | Herb | Hamuka | Leaf | Fresh | Pounded and filtrate is applied as eye drop if not cure transferred to other healer |
| YA 027 | Dahowaso | Unable to urinate | *Acokanthera schimperi* (A. DC.) Schwein | Apocynaceae | Shrub | Betenea | Root | Fresh | Pounded with water and filtrate is taken orally for three days and the residue is applied on waste |
| YA 033 | Utuka | Headache, fever, | *Becium filamentosum* (Forssk.) Chiov. | Lamiaceae | Shrub | Masayali | Leaf | Fresh | Pounded with water and filtrate is applied through nostril |
| YA 052 | Gossen | Flue | *Abutilon figarianum* Guill. & Perro | Malvaceae | Herb | Hamketo | Leaf | Fresh | Chewed and covered with cloth and sniffed/inhaled though nostril |
| YA 034 | Geno | Coughing blood nose and mouth | *Cadaba glandulosa* Forssk. | Capparidaceae | Shrub | Dunelita/Sule | Root | Fresh | Pounded and with water, filtrate is taken orally in morning for three days and residue is used for washing body |
| YA 010 |  |  | *Fagonia schweinfurthii* Hadidi | Zygophyllaceae | Shrub | Huda/ Bedeno | Root | Fresh |  |
| YA 099 | Utuka | Typhoid | *Priva curtisiae* Kobuski | Verbenaceae | Herb | Utuka hara | Leaf | Fresh | Crushed with water and applied through nostrils three times per day for one day |
| YA 098 | Urena | Delayed placenta | *Aloe trichosantha* Berger | Aloaceae | Shrub | Unrena | Leaf and root | Fresh | Root is chewed and juice is swallowed; leaf is Crushed and mixed with water and up to 1 liter filtrate is taken orally |
| YA 024 | Ali Abbya | Snake Bite | *Pergularia tomentosa* L. | Asclepiadaceae | Shrub | Ali Abbya | Leaf | Fresh | Pounded with water and up to 1 lt of filtrate is given orally and drops are applied though nostrils and ears seven times the whole day |
| YA 013 | Halib | Stomachache and bloating | *Indigofera oblongifolia* Forssk. | Fabaceae | Shrub | Ayrowegit (Male) | Root | Fresh | Crushed with water and 1 glass filtrate is given until cured |
| YA 091 | Kudu | Impotence | *Acacia senegal* (L.) Wild. | Fabaceae | Tree | Tiki Bilea | Root | Fresh | Roots are chewed and swallowed and tied on arm |
| YA 078 | Kudu | Impotence | *Cadaba farinosa* Forssk. | Capparidaceae | Shrub | Ormayto | Root | Fresh | Roots are chewed and swallowed and tied on arm |
| YA 099 | Utuka | Headache, fever, | *Priva curtisiae* Kobuski | Verbenaceae | Herb | Utuka hara | Leaf | Fresh | Leaf and root are Pounded and mixed with water, drops filtrate applied through nostrils three times per day for one day |
| YA 008 |  |  | *Balanites rotundifolia* (Van Tiegn.) Blatter | Balanitaceae | Shrub | Alayto | Root | Fresh |  |
| YA 005 | Denabu | Eye Infection | *Cymbopogon commutatus* (Steud.) Stapf | Poaceae | Herb | Afriba | Leaf | Fresh | Crushed with water and tied with close and squeezed and drops are added on eye as eye drops in morning and at bedtime for one day |
| YA 095 | Geno | Chest pain, cough with blood | *Balanites aegyptiaca* (van Tieghem) Blatter | Balanitaceae | Tree | Udayto | Root | Fresh | Pounded with water and 1 glass is taken orally for one day |
| YA 078 |  |  | *Cadaba farinosa* Forssk. | Capparidaceae | Shrub | Ormayto | Root | Fresh |  |
| YA 086 | Utuka | Headache, fever, | *Acalypha fruticosa* Forssk. | Euphorbiaceae | Shrub | Subahsila | Leaf | Fresh | Mixed, Pounded and mixed with water and drops of filtrate are applied through nostrils for three days |
| YA 033 |  |  | *Becium filamentosum* (Forssk.) Chiov. | Lamiaceae | Shrub | Masay Meae | Leaf | Fresh |  |
| YA 104 | Ula Hamma | Infant disease, stomach bloating and green eye coloring | *Silene macrosolen* A. Rich. | Caryophyllaceae | Herb | Werabikela | Leaf | Fresh | Mixed and Pounded together with water and filtrate is applied through nostril for three days |
| YA 045 |  |  | *Acacia oerfota* (Forssk.) Schweinf. | Fabaceae | Tree | Garomto | Leaf and Bark | Fresh |  |
| YA 008 |  |  | *Balanites rotundifolia* (Van Tiegn.) Blatter | Balanitaceae | Shrub | Alayto | Root and Leaf | Fresh |  |
| YA 067 | Gira haro deli | Burnt wound | Selaginella kraussiana (Kunze) A.Braun | Selaginellaceae | Herb | Kuraniba | Whole plant | Fresh | Fried and dried and Pounded and the powder mixed with butter is applied topically on the burnt body part for six days |
| YA 099 | Utuka | Headache, fever, | *Priva curtisiae* Kobuski | Verbenaceae | Herb | Utuka hara | Leaf | Fresh | Pounded and mixed with water, filtrate is given though nostrils for three days |
| YA 027 | Denabu | Eye Infection | Acokanthera schimperi (A. DC.) Schwein | Apocynaceae | Tree | Denabu Hara | Leaf | Fresh | Crushed with water and filtrate is given as eye drop three times for one day |
| YA 076 | Undufeyta | Tooth decay and sickness | *Plicosepalus robustus* Wiens & Polhil | Loranthaceae | Shrub | Merka ato Atokele | Leaf | Fresh | Pounded and the powder mixed with butter is applied topically on teeth for six days |
| YA 074 | Bagemi | Stomach bloating, fever | *Acacia ehrenbergiana* Hayne | Fabaceae | Tree | Mekeni | Bark | Dry | Dried and Pounded and mixed with water 1 glass filtrate is taken orally once per day for seven days |
| YA 001 | Ayali | Infant sickness | *Ocimum urticifolium* Roth | Lamiaceae | Shrub | Ayali Hara | Leaf | Fresh | Pounded and mixed with water, filtrate is given though nostrils and body washing for three days. |
| YA 013 | Andero | Anemia, yellow eye color, | *Indigofera oblongifolia* Forssk. | Fabaceae | Shrub | Ayrowegit (Male) | Leaf | Fresh | Pounded and mixed with water and filtrate is added through nostril and ear for three days and washes body with the residue |
| YA 008 | Ayali | Wof beshita, Infant sickness | *Balanites rotundifolia* (Van Tiegn.) Blatter | Balanitaceae | Shrub | Alayto | Leaf and bark | Fresh | Mixed, pounded mixed with water and 1 liter filtrate is taken orally for seven days; Lekenebo root is crushed with water and filtrate is applied through nostril and remaining is used for body wash for three days |
| YA 086 |  |  | *Acalypha fruticosa* Forssk. | Euphorbiaceae | Shrub | Subahsila | Leaf | Fresh |  |
| YA 045 |  |  | *Acacia oerfota* (Forssk.) Schweinf. | Fabaceae | Tree | Garomto | Leaf and root | Fresh |  |
| YA 070 |  |  | *Indigofera spicata* Forssk. | Fabaceae | Herb | Lekenebo | Root | Fresh |  |
| YA 006 | Tumea | Rush on body, Ekeke | *Plumbago zylanica* L. | Plumbaginaceae | Herb | Aftitosh | Leaf | Fresh | Crushed and applied topically on the infected site, mixed with water and 2 cup of filtrate is taken two times per day for 7 days |
| YA 006 | Tumea | Rush on body, Ekeke | *Plumbago zylanica* L. | Plumbaginaceae | Herb | Aftitosh | Root | Dry | Powdered, mixed with butter and applied on infected part |
| YA 008 | Alsam | Food Poisoning | *Balanites rotundifolia* (Van Tiegn.) Blatter | Balanitaceae | Shrub | Alayto | Root | Fresh | Powdered, mixed with water and 1 glass filtrate is taken orally |
| YA 046 | Takimi Dallo | mouth sores, herpes virus, mumps | *Barleria homoiotrichia* C. B. Clarke | Acanthaceae | Shrub | Geselto | Bark | Fresh | Inner part of bark is pounded and boiled in water and 1 glass of filtrate is taken orally and residue is used body washing; no meet meal is taken |
| YA 087 | Dufduft | Herpes Virus | *Aristolochia bracteolata* Lam. | Aristolochiaceae | Herb | Sue Sue | Leaf | Fresh | Crushed mixed with water and 1 glass filtrate is taken orally three times for one day and residue is used for body wash |
| YA 008 | Itibiake | Eye injury/infection | *Balanites rotundifolia* (Van Tiegn.) Blatter | Balanitaceae | Shrub | Alayto | Root | Fresh | Crushed and soaked in water, filtrate is used as eye drop; until white on eye disappears |
| YA 097 | Geno | Stomachache, fever, dysentery | *Solanum marginatum* Lf | Solanaceae | Shrub | Ungule | Leaf | Fresh | Pounded mixed with water and 1 glass filtrate is taken orally four times per day for four days |
| YA 001 | Utuka | headache, rush on body, block nose, inflammation of gum | *Ocimum urticifolium* Roth | Lamiaceae | Shrub | Abushafi | Leaf | Fresh | Pounded mixed with water, and allowed to settle and 1 glass filtrate is applied through nostril two times per day for one day |
| YA 069 | Baro Ali | Snake Bite | *Ziziphus spina-christi* (L.) Desf. | Rhamnaceae | Tree | Kusra | Young stem | Fresh | Pounded and used for washing the injury site |
| YA 053 | Baro Ali | Snake Bite | *Cocculus pendulus* (J. R. & G. Forst) Diels | Menispermaceae | Climber | Hayuka | Leaf | Fresh | Crushed with water and 1 glass filtrate per day is taken orally for 7 days |
| YA 078 | Iyala | Infant sickness | *Cadaba farinosa* Forssk. | Capparidaceae | Shrub | Galikihna | Leaf | Fresh | Pounded with water and filtrate is applied through nostril once per day for two days; residue is used for body wash |
| YA 069 | Alsam | Food Poisoning | *Ziziphus spina-christi* (L.) Desf. | Rhamnaceae | Tree | Galiela | Stem and leaf | Fresh | Pounded with water and 1 glass filtrate is taken orally for one day |
| YA 019 | Asso | Malaria | *Acalypha indica* L. | Euphorbiaceae | Herb | Baroberberie | Leaf | Fresh | Crushed with water and 1 glass filtrate is taken orally for one day |
| YA 052 | Aweyto | Swelling on body/ Sores on body | *Abutilon figarianum* Guill. & Perro | Malvaceae | Herb | Hamuka | Latex | Fresh | Applied on the swollen part to make it burst and until cure |
| YA 019 | Geno | Stomachache, fever, dysentery | *Acalypha indica* L. | Euphorbiaceae | Herb | Baroberberie | Leaf | Fresh | Pounded mixed in water 1 glass filtrate is taken orally four times per day until cure |
| YA 087 | Daleali | Infant sickness, swelling on body (bgunge) | *Aristolochia bracteolata* Lam. | Aristolochiaceae | Herb | Sue Sue | Leaf | Fresh | Pounded with water 1 coffee cup filtrate is given orally and washing body for seven days |
| YA 008 | Anti Biak | Eye injury/infection | *Balanites rotundifolia* (Van Tiegn.) Blatter | Balanitaceae | Shrub | Alayto | Stem | Fresh | Pounded with water used as eye drops three times per day for three days |
| YA 019 | Asso | Malaria | *Acalypha indica* L. | Euphorbiaceae | Herb | Baroberberie | Leaf | Fresh | Crushed with water and 1 glass filtrate for one day |
| YA 008 | Geno | Stomachache, fever, bloody dysentery, vomiting | *Balanites rotundifolia* (Van Tiegn.) Blatter | Balanitaceae | Shrub | Alayto | Leaf | Fresh | Pounded and boiled in water, 1 glass cold filtrate is given orally in morning and evening for foyr days |
| YA 015 | Urufea | Vomiting, dysentery | *Indigofera articulata* Gouan | Fabaceae | Shrub | Ayrowegit | Leaf and stem | Fresh | Pounded with water and 1 glass filtrate is taken orally three times per day for three days |
| YA 062 | Gosom | Coughing, running nose, flue | *Grewia erythraea* Schweinf. | Tiliaceae | Shrub | Adayto | Bark | Fresh | Inner part of bark is chewed and juice is swallowed three times per day for two days |
| YA 038 | Leweli | Stomachache and bloating, | *Mimusops kummel* A. DC. | Sapotaceae | Tree | Gabu | Root | Fresh | Pounded and mixed with water and 1 glass filtrate is given orally in the morning for three days |
| YA 082 | Baro geie | Swelling on body | *Senna alexandrina* Mill. | Fabaceae | Shrub | Senu | Leaf | Fresh | Leaf and *T. foenum-graecum* seed is pounded and mixed with sheep milk and applied on the swollen part for three days. when bursts *T. foenum-graecum* powder mixed with butter is applied in the wound |
| YA 043 | Undufeyta | Teeth decay and sickness, tongue and mouth, throat, head infection | *Calotropis procera* (Ait.) Ait.f. | Apocynaceae | Shrub | Geselto | Bark | Fresh | Pounded with water and 1 glass filtrate is taken early morning for two days; dry root is powdered and mixed with *T. foenum-graecum* seed powder and milk applied on the infected area or wound for two days |
| YA 013 | Geno | Stomachache, fever, blood comes out through mouth and nose, dysentery | *Indigofera oblongifolia* Forssk. | Fabaceae | Shrub | Ayrowegit (Male) | Root | Fresh | Pounded and mixed with water and 1 glass filtrate is given orally in the morning for two days |
| YA 015 | Baro Hara | Snake Bite | *Indigofera articulata* Gouan | Fabaceae | Shrub | Ayrowegit | Root | Fresh | Pounded and boiled; 1 glass filtrate is given for three days |
| YA 087 | Baro Hara | Snake Bite | *Aristolochia bracteolata* Lam. | Aristolochiaceae | Herb | Sue Sue | Root | Fresh | Pounded and boiled; 1 glass filtrate is given for two days |
| YA 051 | Besengli | Chest pain, cough | *Polygala obtusissima* Chod. | Polygalacea | Shrub | Halmela | Root | Fresh | Chewing and swallowing juice and infants; crushed and mixed with water filtrate is taken orally for two days |
| YA 087 | Undufeyta | Infection on skin | *Aristolochia bracteolata* Lam. | Aristolochiaceae | Herb | Sue Sue | Leaf and root | Fresh | Pounded with water and 1 glass filtrate is taken early morning for two days; dry root powder with water and filtrate is applied as nasal drops and used for washing body two times per day for three days |
| YA 019 | Asso | Malaria | *Acalypha indica* L. | Euphorbiaceae | Herb | Baroberberie | Upper part | Fresh | Pounded and mixed with Milk and 1 glass is taken orally twice per day for three days |
| YA 036 | Giret hararo | Fire Burned body | *Solanum somalense* Franchet. | Solanaceae | Shrub | Felina meae | Upper part | Dry | Fried and dried; ground, powder mixed with butter and applied topically on the burned body part for three days |
| YA 028 | Dahowaso | Unable to urinate | *Cucumis prophetarum* L. | Cucurbitaceae | Climber | Denkakebis | Young branch | Fresh | Crushed with water and 1 glass filtrate is given orally early morning for three days |
| YA 019 | Gano | Headache, fever | *Acalypha indica* L. | Euphorbiaceae | Herb | Baroberberie | Leaf | Fresh | Leaf pounded with water and filtrate is applied as nasal drop for three days and used for washing body; |
| YA 086 | Gano | Headache, fever | *Acalypha fruticosa* Forssk. | Euphorbiaceae | Shrub | Subahsila | Root | Fresh | if not cured root is Crushed and filtrate is applied as nasal drops for three days |
| YA 046 | Dareta | Swelling on skin | *Barleria homoiotrichia* C. B. Clarke | Acanthaceae | Shrub | Geselto | Bark | Fresh | Crushed with water and boiled 1 glass filtrate is given orally for days until cure |
| YA 032 | Baro Ali | Snake Bite | *Moringa oleifera* Lam. | Moringaceae | Tree | Dinkil Iliasi/ Lakasi | Root bark | Fresh | chewed and juice is swallowed and applied on the wound for seven days |
| YA 036 | Giret hararo | Burned body part | *Solanum somalense* Franchet. | Solanaceae | Shrub | Felina meae | Young branch | Dry | Dried with fire and powder mixed with butter is applied on burned body part for three days |
| YA 028 | Dahowaso | Unable to urinate | *Cucumis prophetarum* L. | Cucurbitaceae | Climber | Denkakebis | Young branch and Fruit | Fresh | Mixed and pounded with water and 1 glass filtrate is given orally for two days |
| YA 075 | Girad Edradenu | Bullet injury | *Acacia mellifera* (Vahl) Benth. | Fabaceae | Tree | Marka ato | Bark, Stem | Fresh | Bark is tied on the wound and stem is dried on fire and powder with butter is tied on the wound for six days |
| YA 008 |  |  | *Balanites rotundifolia* (Van Tiegn.) Blatter | Balanitaceae | Shrub | Aragadis | Bark, Stem | Fresh |  |
| YA 006 | Dareta | Swelling on body/ Sores on body | *Plumbago zylanica* L. | Plumbaginaceae | Herb | Hindili | Lear | Fresh | Leaf is Pounded with water and applied topically on the infected part, and 1 glass filtrate is given orally for 3 days |
| YA 008 | Alsam | vomiting, dysentery | *Balanites rotundifolia* (Van Tiegn.) Blatter | Balanitaceae | Shrub | Alayto | Root | Fresh | Ground and mixed with water and 2 glass filtrate is given orally before breakfast for three days and washes body with residue |
| YA 062 | Iyala | Infant Sickness | *Grewia erythraea* Schweinf. | Tiliaceae | Shrub | Adayto | Leaf and Root | Fresh | Pounded mixed water and filtrate is applied through nostril for one day and body is washed with mixture for seven days |
| YA 008 | Undufeyta | head, body, and mouth infection, herpes | *Balanites rotundifolia* (Van Tiegn.) Blatter | Balanitaceae | Shrub | Ayrowegit | Leaf | Fresh | Crushed mixed with water and few drops are given through nostril; wash mouth and with residue for washing only head |
| YA 017 | Ulhama | Infant Sickness | *Trigonella foenum-graecum* L. | Fabaceae | Herb | Abaka(Abish) | Seed | Dry | Powder mixed with cattle dung is wash body of infant three times per day |
| YA 027 | Denabu | Eye infection/injury | *Acokanthera schimperi* (A. DC.) Schwein | Apocynaceae | Shrub | Karora | Latex | Fresh | Mixed with water applied as an eye drop two times per day for three days |
| YA 053 | Lahitya | Breast infection | *Cocculus pendulus* (J. R. & G. Forst) Diels | Menispermaceae | Climber | Hayuka | Root | Fresh | Crushed and topically applied on beast until swelling bursts and cure |
| YA 012 | Baro Hali | Snake bite | *Rhus natalensis* Krauss | Anacardiaceae | Tree | Duwa | Root | Fresh | Crushed and topically tied on swollen part and chew root and swallow juice |
| YA 050 | Duduba | Snake bite | *Seddera bagshawei* Rendle | Convolvulaceae | Shrub | Buran Hira | Leaf | Fresh | Crushed and tied on the wound for three days |
| YA 095 | Geno | Headache, blockage of nose | *Balanites aegyptiaca* (van Tieghem) Blatter | Balanitaceae | Tree | Udayto | Root Bark | Fresh | Pounded mixed with water and filtrate applied through nostrils and goat butter is added for one day |
| YA 036 | Utuka, | Typhoid, typhus, Mitch, headache, fever | *Solanum somalense* Franchet. | Solanaceae | Shrub | Filina eli | Leaf and Stem | Fresh | Put on hot charcoal and smoke is inhaled at night for three days |
| YA 066 | Undufeyta | head, mouth and ear infection and sores | *Commicarpus helenae* (J.A. Schultes) Meikle | Nyctaginaceae | Herb | Seati | Root | Fresh | Mixed, crushed with water and filtrate is applied through nostrils and paste is applied on the infected body parts |
|  |  |  |  | Nyctaginaceae | Herb | Seati | Leaf | Fresh |  |
| YA 066 | Aweeta | Hand and Foot Swelling | *Commicarpus helenae* (J.A. Schultes) Meikle | Nyctaginaceae | Herb | Seati | Leaf | Fresh | Leaf is fasted on swollen part and if not burst will be referred to other healer |
| YA 087 | Antibiak | Eye infection/injury | *Aristolochia bracteolata* Lam. | Aristolochiaceae | Herb | Siude | Leaf | Fresh | Crushed with water and filtrate is applied as eye drops three times per day for three days |
| YA 021 | Degar Liena | Fever | *Ruellia patula* Jacq. | Acanthaceae | Shrub | Meriyesi | Whole plant | Fresh | Crushed with water and filtrate is taken orally for the whole day and used for washing body; If not cured stem bark crushed with water and filtrate is given orally for the whole day |
| YA 062 |  |  | *Grewia erythraea* Schweinf. | Tiliaceae | Shrub | Desayto | Bark | Fresh |  |
| YA 015 | Tuma wuha, Duduba | Swelling on body | *Indigofera articulata* Gouan | Fabaceae | Shrub | Ayrowegit | Leaf | Fresh | Crushed and applied on the infected body parts and 1 glass filtrate is given orally for one day |
| YA 040 | Sikato | Swelling on skin | *Withania somnifera* (L.) Dunal | Solanaceae | Shrub | Buran Hinhara | \Leaf | Fresh | Crushed and applied on infected part of body; Abish seed fried and powdered and mixed with butter is applied on the wound; and put on fire and smoke is used as used as treatment |
| YA 002 | Bekea | Head wound | *Sericocomopsis pallida* (S. Moore) Schinz | Amaranthaceae | Shrub | Ademagari | Leaf | Dry | Dried in sun and powder is applied on the wound daily until cure |
| YA 062 | Bado Gide | Leprosy | *Grewia erythraea* Schweinf. | Tiliaceae | Shrub | Hidayto | Root | Fresh | Pounded and mixed with sheep milk and applied on the infected part until cure |
| YA 077 | Dahowaso | Unable to urinate | *Aerva javanica* (Burm.f) Schultes | Amaranthaceae | Herb | Olayto | Root | Fresh | Crushed with water and filtrate is given orally for whole day and residue is used to wash body |
| YA 002 | Bekaie | Head wound | *Sericocomopsis pallida* (S. Moore) Schinz | Amaranthaceae | Shrub | Admamegari | Leaf | Fresh | Crushed and mixed with butter and applied on wound until cure |
| YA 062 | Utuka, | Headache, fever | *Grewia erythraea* Schweinf. | Tiliaceae | Shrub | Adayto | Root | Fresh | Crushed and mixed water and used for washing body for seven days |
| YA 095 | Geno | Mitch | *Balanites aegyptiaca* (van Tieghem) Blatter | Balanitaceae | Tree | Udayto | Root | Fresh | Pounded and mixed with water and given orally in place of water for the three days and washes body |
| YA 040 | Wayib | sores on skin | *Withania somnifera* (L.) Dunal | Solanaceae | Shrub | Burahin hara | Leaf | Fresh | Crushed with water and washed body and filtrate is applied drops through nostrils for two days |
| YA 008 | Utuka, | Mitch | *Balanites rotundifolia* (Van Tiegn.) Blatter | Balanitaceae | Shrub | Alayto | Bark | Fresh | Pounded and mixed with water and filtrate is applied through nostrils for three days |
| YA 020 | Itibiake | Eye infection/injury | *Kleinia squarrosa* Cufod. | Asteraceae | Shrub | Beyleli | Leaf | Fresh | Pounded and covered with cloth and juice is used as eye drops for two days or until cure |
| YA 046 | Mesangellea | Chest pain/unable to breath and coughing, fever | *Barleria homoiotrichia* C. B. Clarke | Acanthaceae | Shrub | Boboa | Root | Fresh | Pounded and mixed with water and 1 lt filtrate is taken orally for three days |
| YA 009 | Alabala | Sores on skin/Leprosy | *Heliotropium cinerascens* DC. & A. DC. | Boraginaceae | Herb | Amada | Leaf | Fresh | Pounded and mixed with butter and applied on the infected body part for three days |
| YA 008 | Bekaie | Head wound/Injury | *Balanites rotundifolia* (Van Tiegn.) Blatter | Balanitaceae | Shrub | Alayto | Leaf | Fresh | Pounded and applied on the wound until cure |
