# Supplementary material for: An ethnobotanical survey of medicinal and edible plants of Yalo Woreda in Afar regional state, Ethiopia
Source: J Ethnobiol Ethnomed. 2017 Jul 5;13:40. doi: 10.1186/s13002-017-0166-7 (PMC5499056; doi:10.1186/s13002-017-0166-7)
Supplement: Supplementary file 2 — Medicinal plants used to treat animal illness, Yalow Woreda, 2016. (B = bark, C = climber, F = flower, Fr = fruit, L = leaf, La = latex, R = root, S = stem, Br = branch, UP = upper part WP = whole plant, YP = young plant). (DOCX 66 kb) [file 13002_2017_166_MOESM2_ESM.docx]

**Additional File 2 Medicinal plants used to treat animal illness, Yalow Woreda, 2016. (B = bark, C= climber, F = flower, Fr = fruit, L = leaf, La = latex, R = root, S = stem, Br = branch, UP = upper part WP = whole plant, YP = young plant)**

| **Voucher Number** | **Local name Disease** | **Disease** | **Scientific Name** | **Family** | **Habit** | **Local Name Plant** | **Part** | **Condition** | **Method of Preparation and application** | **Animal** |
| --- | --- | --- | --- | --- | --- | --- | --- | --- | --- | --- |
| YA 075 | Delie | External infection | *Acacia mellifera* (Vahl) Benth. | Fabaceae | T | Marka ato | S | Fresh | Charred, powdered mixed with skimmed goat milk applied topically on the infected body part | Camel |
| YA 076 | Denabu | PPR | *Acacia mellifera* (Vahl) Benth. | Fabaceae | T | Marka ato | L | Fresh | Chewed and spitted on the eye in morning for 3 days | Cattle |
| YA 045 | Adroyta | Sheep and Goat pox | *Acacia oerfota* (Forssk.) Schweinf. | Fabaceae | T | Geromto | L | Fresh | Crushing and mixed with boiled milk and given orally in the morning | Goat |
| YA 045 | Kida | Mastitis and contagious agalactia | *Acacia oerfota* (Forssk.) Schweinf. | Fabaceae | T | Geromto | L | Fresh | Crushed, soaked in water for a night; the filtrate is applied through nostrils and the residue used to wash breast of animal for a day | Cow, goat, Camel |
| YA 086 | Ladorea | Blackleg | *Acalypha fruticosa* Forssk. | Euphorbiaceae | S | Subahsila | L | Fresh | Crushed with water and one tin can filtrate is given orally and through nostril for one day | Camel, cattle, goat |
| YA 087 | Kida | Bovine pasteurellosis | *Acalypha fruticosa* Forssk. | Euphorbiaceae | S | Subahsila | L | Fresh | Mixed and pounded together with water and applied through nostril and one tin can is given orally for 1 to 3 days | Cattle |
| YA 088 | Geno | Anthrax | *Acalypha fruticosa* Forssk. | Euphorbiaceae | S | Subahsila | L | Fresh | Mixed and pounded together with water and applied through nostril and one tin can is given orally for 1 to 3 days | Camel, Cattle, goat |
| YA 089 | Gubllo | CCPP | *Acalypha fruticosa* Forssk. | Euphorbiaceae | S | Subahsila | L | Fresh | Pounded with water and filtrate is given orally and through nostril | Camel, Cattle, goat |
| YA 090 | Diglo | Bone breakage/ fractures | *Acalypha fruticosa* Forssk. | Euphorbiaceae | S | Subahsila | L | Fresh | Mixed, crushed and mixed with sheep blood tied on broken bone area and removed after two days | Cattle, goat |
| YA 019 | Baro Ali | Snake Bite | *Acalypha indica* L. | Euphorbiaceae | H | Baroberberie | L | Fresh | Crushed, soaked in water and 2 glass for goat and 2 lt filtrate is given for camel and cow orally and through nostril | Goat, Cow, camel, |
| YA 020 | Kida | Mastitis and contagious agalactia | *Acalypha indica* L. | Euphorbiaceae | H | Baroberberie | L | Fresh | Crushed and mixed in water and 3 lt is given orally, and applied through nostril and ear residue is used to rub the breast morning and evening for 2 days | Cow, camel, goat |
| YA 027 | Iti Biak | PPR | *Acokanthera schimperi* (A. DC.) Schwein | Apocynaceae | T | Denabu Hara | L | Fresh | Crushed, soaked in water and 2 glass for goat and 2 lt of filtrate for camel and cow is given orally and through nostril | Goat, Cattle Camel |
| YA 077 | Kida | Mastitis and contagious agalactia | *Aerva javanica* (Burm.f) Schultes | Amaranthaceae | H | Olayto | L | Fresh | Pounded and soaked in water and filtrate is given orally, through nostrils, and topically applies on breast for three days and remaining used for body washing | Goat, cow |
| YA 077 | Utka | PPR | *Aerva javanica* (Burm.f) Schultes | Amaranthaceae | H | Olayto | R | Fresh | Crushed and soaked in water overnight and filtrate mixed with goat skimmed milk is applied through nostrils | Goat |
| YA 098 | Likek | Black leg/ joint illness | *Aloe trichosantha* Berger | Aloaceae | S | Urina | R | Fresh | Three roots, pepper and salt are inserted in cut made on the body and kept for 3 day | Cow, oxen |
| YA 098 | Kida | Brucellosis | *Aloe trichosantha* Berger | Aloaceae | S | Urina | L, S | Fresh | Crushed and mixed in water and 3 lt is given orally, and applied through nostril and ear residue is used to rub the breast in morning and evening for 2 days | Camel, cow, goat |
| YA 095 |  |  | *Balanites aegyptiaca* (van Tieghem) Blatter | Balanitaceae | T | Udayto | L | Fresh | Pounded and mixed with water and filtrate is given orally once | Cow, goat |
| YA 095 | Geno | Blackleg | *Balanites aegyptiaca* (van Tieghem) Blatter | Balanitaceae | T | Udayto | R | Fresh | Pounded and mixed with water and filtrate is taken orally once | Cow, goat |
| YA 095 | Geremole | Trypanosomiasis | *Balanites aegyptiaca* (van Tieghem) Blatter | Balanitaceae | T | Udayto | R | Fresh | Pounded and mixed with water and filtrate is given orally once | Cow, goat |
| YA 095 | Indahi | CCPP | *Balanites aegyptiaca* (van Tieghem) Blatter | Balanitaceae | T | Udayto | R | Fresh | Pounded and mixed with water and filtrate is given orally once | Cow, goat |
| YA 008 | Likek | Blackleg | *Balanites rotundifolia* (Van Tiegn.) Blatter | Balanitaceae | S | Alayto | R | Fresh | Crushed and soaked in water overnight and filtrate mixed with goat skimmed milk is applied through nostrils | Goat |
| YA 078 | Kida | Mastitis and contagious agalactia | *Cadaba farinosa* Forssk. | Capparidaceae | S | Ormayto | L | Fresh | Crushed and mixed in water and 3 lt is given orally, and applied through nostril and ear and residue is used to rub the breast in morning and evening for 2 days | Cow, goat |
| YA 078 | Geno | Anthrax | *Cadaba farinosa* Forssk. | Capparidaceae | S | Ormayto | YP | Fresh | Crushed and mixed with water and all cow and oxen are given 4 lt orally as vaccine | Oxen |
| YA 003 | Dulaya | Retained Placenta | *Cadaba rotundifolia* Forssk | Capparidaceae | S | Adengelli | R | Fresh | Crushed with water and 1 tin can is given orally and applied through nostril for three days | Camel, Cattle |
| YA 003 | Lahidelea | External parasite | *Cadaba rotundifolia* Forssk | Capparidaceae | S | Adengelli | R | Fresh | Crushed and applied topical on body for three days | Cow, goat, sheep |
| YA 003 | Delita | Brucellosis | *Cadaba rotundifolia* Forssk | Capparidaceae | S | Adengelli | WP | Fresh | Pounded, soaked in water and 2 lt is given orally | Goat, camel, cow |
| YA 003 | Dulaya | ORF | *Cadaba rotundifolia* Forssk | Capparidaceae | S | Adengelli | UP | Fresh | Pounded, soaked in water and 2 lt is given orally | Goat, sheep |
| YA 003 | Delie | CCPP | *Cadaba rotundifolia* Forssk | Capparidaceae | S | Adengelli | WP | Fresh | Pounded, soaked in water and 2 lt is given orally | Goat |
| YA 003 | Begofoy | Bloating | *Cadaba rotundifolia* Forssk | Capparidaceae | S | Adengelli | R | Fresh | Pounded, soaked in water and 2 lt is given orally | Cattle |
| YA 043 | Likek | Black leg | *Calotropis procera* (Ait.) Ait.f. | Apocynaceae | S | Gelato | YP | Fresh | Crushed and macerated in water and given orally and through nostril morning for one day for all cattle as vaccine | Cattle |
| YA 043 | Geno | Anthrax | *Calotropis procera* (Ait.) Ait.f. | Apocynaceae | S | Gelato | S, L | Fresh | Crushed and mixed with water and all cow and oxen are given 4 lt orally as vaccine | Cattle |
| YA 043 | Kida | Mastitis and contagious agalactia | *Calotropis procera* (Ait.) Ait.f. | Apocynaceae | S | Gelato | L | Fresh | Crushed mixed with water and 1 lt filtrate is given two times per day in morning and evening for seven days. | Camel |
| YA 043 | Kida | Mastitis and contagious agalactia | *Calotropis procera* (Ait.) Ait.f. | Apocynaceae | S | Gelato | L | Fresh | Pound, soaked in water and 1 lt filtrate is given orally and through nostril; the remaining is used for body washing | Cow |
| YA 085 | Geno | Anthrax | *Capparis decidua* (Forssk.) Edgew. | Capparidaceae | S | Sonkordo | S | Fresh | Crushed and mixed in water 3 lt filtrate is given for all cattle orally once as vaccine | Cattle |
| YA 061 | Gali Biak | Pastuerollosis | *Capsicum frutescens* L., | Solanaceae | S | Hindi | Fr | Dry | Powdered and soak in water and 1 tin can is given through nostrils and orally for one day | Camel |
| YA 103 | Likek | Blackleg | *Celosia polystachia* (Forssk.) C.C. Townsend | Amaranthaceae | H | Werabikela | R | Fresh | Crushed with water and 1 tin filtrate is given orally and through nostril for one day | Cattle |
| YA 103 | Kida | Mastitis and contagious agalactia | *Celosia polystachia* (Forssk.) C.C. Townsend | Amaranthaceae | H | Werabikela | L | Fresh | Crushed and mixed in water and 3 lt is given orally, and applied through nostril and ear residue is used to rub the breast morning and evening for 2 days | Camel, cow, goat |
| YA 044 | Geno | Swelling on neck, chest, lung infection, | *Cissus quadrangularis* L. | Vitaceae | C | Gemele | YP | Fresh | Crushed and macerated in water and 1 tin can is given orally and through nostril in morning for one day for all cattle as vaccine | Cattle |
| YA 025 | Kida | Mastitis and contagious agalactia | *Citrullus lanatus* (Thunb.) Matsum. & Nakai | Cucurbitaceae | C | Dare Ebto | L | Fresh | Crushed, soaked in water for a night; the filtrate is applied through nostrils and the residue used to wash breast for a day | Goat, camel, cow |
| YA 056 | Silaytu | Trypanosomiasis | *Commicarpus squarrosus* (Heimerl) Standl. | Nyctaginaceae | S | Hasahada | S | Dry | Powdered, mixed with butter given through nostrils, ear and applied topically on body once a day for three days | Camel |
| YA 072 | Dele | Topical wound infection | *Cordia sinensis* Lam. | Boraginaceae | T | Madera | B | Fresh | Crushed and applied in the wound for three days | Cow, goat, Camel |
| YA 028 | Lahidelea | Skin infection | *Cucumis prophetarum* L. | Cucurbitaceae | C | Denkakebis | L | Dry | Crushed with water and 1 tin is given orally and applied through nostril for three days | Cattle |
| YA 037 | Sangiti | Foot and mouth | *Dichrostachys cinerea* (L.) Wight & Am. | Fabaceae | S | Gaboyta | B | Fresh | Crushed mixed with diseased cow's gut paste and inserted in the cut made topically | Cattle, goat, sheep |
| YA 042 | Geno | Anthrax | *Dobera glabra* (Forssk.) Poir. | Salvadoraceae | S | Gasera | L | Fresh | Crushed and mixed with water 3 lt is given for all cattle orally once | Cow, oxen |
| YA 042 | Begofoy | Bloating | *Dobera glabra* (Forssk.) Poir. | Salvadoraceae | S | Gasera | R | Fresh | Pounded, soaked in water and 2 lt is given orally | Cattle |
| YA 042 | Tumea | Blackleg | *Dobera glabra* (Forssk.) Poir. | Salvadoraceae | S | Gasera | R,L | Fresh | Pounded, soaked in water and 2 lt is given orally | Cattle |
| YA 042 | Halawas | Skin infection | *Dobera glabra* (Forssk.) Poir. | Salvadoraceae | S | Gasera | L | Fresh | Crushed and applied on the infection for three days | Cattle, camel |
| YA 064 | Kalit | Goat dysentery | *Euphorbia triaculeata* Forssk. | Euphorbiaceae | S | Ingdato | L | Fresh | Crushed and filtrate is given through nostrils and orally | Goat |
| YA 064 | Korbahi | Orf | *Euphorbia triaculeata* Forssk. | Euphorbiaceae | S | Ingdato | L | Fresh | Crushed with wooden pestle and mortar soaked over night and given early morning orally or through nostrils | Goat |
| YA 048 | Sangiti | Foot and mouth | *Grewia villosa* Willd. | Tiliaceae | S | Habeleyta | S | Dry | Pounded and mixed with water and I tin can filtrate is give orally and through nostrils once | Cattle, goat, sheep |
| YA 009 | Lahidelea | Skin infection | *Heliotropium cinerascens* DC. & A. DC. | Boraginaceae | H | Amaeda | L,R | Fresh | Crushed and applied in the wound for three days | Cow, goat, Camel |
| YA 013 | Sangiti | Foot and mouth | *Indigofera oblongifolia* Forssk. | Fabaceae | S | Ayrowegit (Male) | GP | Fresh | Cut is made and a diseased cow's gut paste is inserted in the cut | Cattle |
| YA 022 | Dulaya | Retained placenta | *Justicia schimperiana* (Hochst. ex Nees) T. Anders. | Acanthaceae | S | Boroseley | R | Fresh | Crushed and macerated in water for 1 hour and 3 lt filtrate is given orally | Camel |
| YA 012 | Delie | Topical wound, External body infection | *Rhus natalensis* Krauss | Anacardiaceae | T | Atimi | L | Fresh | Charred, powdered mixed with skimmed goat milk and applied topically on the infected body part | Camel, Cattle |
| YA 067 | Geno | Anthrax | *Selaginella kraussiana* (Kunze) A.Braun | Selaginellaceae | H | Kuraniba | UP | Fresh | Mixed and pounded together with water and applied through nostril and one tin can is given orally for 1 to 3 days | cattle, goat, sheep |
| YA 083 | Dulaya | Retained placenta | *Senna italica* Mill. | Fabaceae | H | Senu | S | Fresh | Powdered soaked with water 3 lt filtrate is given orally once | Camel |
| YA 100 | Likek | Blackleg | *Solanum incanum* L. | Solanaceae | S | Wakrikoso | R | Fresh | Pounded and filtrate is given orally and through nostrils and applied on swollen part | Cattle |
| YA 100 | Mudunta | CCPP | *Solanum incanum* L. | Solanaceae | S | Wakrikoso | R | Fresh | Pounded with water and filtrate is given orally and nasal | Goat |
| YA 100 | Mudunta | Pneumonia (CBPP) | *Solanum incanum* L. | Solanaceae | S | Wakrikoso | R | Fresh | Pounded with water and filtrate is given orally and nasal | Camel |
| YA 095 | Kida | Breast swelling, infection | *Balanites aegyptiaca* (van Tieghem) Blatter | Balanitaceae | T | Udayto | L | Fresh | All mixed and pounded mixed with water and one tin can filtrate is given orally once | Cow, goat |
| YA 086 |  |  | *Acalypha fruticosa* Forssk. | Euphorbiaceae | S | Subahsila | L | Fresh |  |  |
| YA 015 |  |  | *Indigofera articulata* Gouan | Fabaceae | S | Ayrowegit | L | Fresh |  |  |
| YA 035 |  |  | *Acacia tortilis* (Forssk.) Schweinf. | Fabaceae | T | Eabeto | L | Fresh |  |  |
| YA 065 |  |  | *Bourreria orbicularis* (Hutch. & E.A. Bruce) Thulin | Boraginaceae | S | Inguleyta | L | Fresh |  |  |
| YA 104 |  |  | *Silene macrosolen* A. Rich. | Caryophyllaceae | H | Werasibila | L | Fresh |  |  |
| YA 068 |  |  | *Ziziphus mauritiana* Lam. | Rhamnaceae | T | Kusra | L | Fresh |  |  |
| YA 078 |  |  | *Cadaba farinosa* Forssk. | Capparidaceae | S | Ormayto | L | Fresh |  |  |
| YA 008 |  |  | *Balanites rotundifolia* (Van Tiegn.) Blatter | Balanitaceae | S | Alayto | L | Fresh |  |  |
| YA 013 | Geno | Bovine pastuerollosis | *Indigofera oblongifolia* Forssk. | Fabaceae | S | Ayrowegit (Male) | L | Fresh | All mixed, crushed with water and one tin can filtrate is given orally in morning and evening for one day | Cattle |
| YA 034 |  |  | *Cadaba glandulosa* Forssk. | Capparidaceae | S | Dunelita | L | Fresh |  |  |
| YA 008 |  |  | *Balanites rotundifolia* (Van Tiegn.) Blatter | Balanitaceae | S | Alayto | L | Fresh |  |  |
| YA 095 |  |  | *Balanites aegyptiaca* (van Tieghem) Blatter | Balanitaceae | T | Udayto | L | Fresh |  |  |
| YA 097 |  |  | *Solanum marginatum* Lf | Solanaceae | S | Ungule | L | Fresh |  |  |
| YA 086 |  |  | *Acalypha fruticosa* Forssk. | Euphorbiaceae | S | Subahsila | L | Fresh |  |  |
| YA 078 |  |  | *Cadaba farinosa* Forssk. | Capparidaceae | S | Ormayto | L | Fresh |  |  |
| YA 045 | Kida | Mastitis and contagious agalactia | *Acacia oerfota* (Forssk.) Schweinf. | Fabaceae | T | Geromto | L | Fresh | All mixed, crushed, and soaked in water for a night; the filtrate is applied through nostrils and the residue used to wash breast for a day | Cow, goat, Camel |
| YA 008 |  |  | *Balanites rotundifolia* (Van Tiegn.) Blatter | Balanitaceae | S | Alayto | L | Fresh |  |  |
| YA 066 |  |  | *Commicarpus helenae* (J.A. Schultes) Meikle | Nyctaginaceae | H | Kerebto | L | Fresh |  |  |
| YA 025 |  |  | *Citrullus lanatus* (Thunb.) Matsum. & Nakai | Cucurbitaceae | C | Dare Ebto | L | Fresh |  |  |
| YA 010 |  |  | *Fagonia schweinfurthii* Hadidi | Zygophyllaceae | S | Arengali | WP | Fresh |  |  |
| YA 019 | Baro Ali | Snake Bite | *Acalypha indica* L. | Euphorbiaceae | H | Baroberberie | L | Fresh | Crushed, soaked in water and 2 glass for goat and 2 lt of filtrate for camel and cow is given orally and through nostril | Cow, camel, goat |
| YA 008 | Utka | PPR | *Balanites rotundifolia* (Van Tiegn.) Blatter | Balanitaceae | S | Alayto | R | Fresh | Crushed and soaked in water overnight and filtrate mixed with goat skimmed milk is applied through nostrils | Goa, sheep |
| YA 086 | Geno | Bloody dysentery, stomach bloating | *Acalypha fruticosa* Forssk. | Euphorbiaceae | S | Subahsila | L | Fresh | All mixed and pounded with water, and mixed with 15 lt of water and given orally to cattle for one day | Cattle |
| YA 019 |  |  | *Acalypha indica* L. | Euphorbiaceae | H | Baroberberie | L | Fresh |  |  |
| YA 004 |  |  | *Allium sativum* L. | Aloaceae | H | Ado besel | Fr | Fresh |  |  |
| YA 033 |  |  | *Becium filamentosum* (Forssk.) Chiov. | Lamiaceae | H | Dob Dob | L | Fresh |  |  |
| YA 103 |  |  | *Celosia polystachia* (Forssk.) C.C. Townsend | Amaranthaceae | H | Werabikela | L | Fresh |  |  |
| YA 094 |  |  | *Ferula communis* L. | Apiaceae | H | Ubewused | Fr | Fresh |  |  |
| YA 013 |  |  | *Indigofera oblongifolia* Forssk. | Fabaceae | S | Ayrowegit (Male) | L | Fresh |  |  |
| YA 045 | Geno | Swelling on neck, chest, lung infection, | *Acacia oerfota* (Forssk.) Schweinf. | Fabaceae | T | Geromto | R | Fresh | All mixed crushed and macerated in water and given orally and through nostril in morning for one day for all cattle as vaccine | Cattle |
| YA 095 |  |  | *Balanites aegyptiaca* (van Tieghem) Blatter | Balanitaceae | T | Udayto | Ba | Fresh |  |  |
| YA 008 |  |  | *Balanites rotundifolia* (Van Tiegn.) Blatter | Balanitaceae | S | Alayto | YP | Fresh |  |  |
| YA 003 |  |  | *Cadaba rotundifolia* Forssk | Capparidaceae | S | Adengelli | L | Fresh |  |  |
| YA 065 |  |  | *Bourreria orbicularis* (Hutch. & E.A. Bruce) Thulin | Boraginaceae | S | Inguleyta | Fr | Fresh |  |  |
